# Human Tissues Contain CD141^hi^ Cross-Presenting Dendritic Cells with Functional Homology to Mouse CD103^+^ Nonlymphoid Dendritic Cells

**DOI:** 10.1016/j.immuni.2012.04.012

**Published:** 2012-07-27

**Authors:** Muzlifah Haniffa, Amanda Shin, Venetia Bigley, Naomi McGovern, Pearline Teo, Peter See, Pavandip Singh Wasan, Xiao-Nong Wang, Frano Malinarich, Benoit Malleret, Anis Larbi, Pearlie Tan, Helen Zhao, Michael Poidinger, Sarah Pagan, Sharon Cookson, Rachel Dickinson, Ian Dimmick, Ruth F. Jarrett, Laurent Renia, John Tam, Colin Song, John Connolly, Jerry K.Y. Chan, Adam Gehring, Antonio Bertoletti, Matthew Collin, Florent Ginhoux

**Affiliations:** 1 Institute of Cellular Medicine, Newcastle University, Newcastle upon Tyne NE2 4HH, UK; 2 Singapore Immunology Network, Agency for Science, Technology and Research (A^∗^STAR), 138648, Singapore; 3 Singapore General Hospital, 169608, Singapore; 4 University of Glasgow Centre for Virus Research, University of Glasgow, Glasgow G61 1QH, UK; 5 National University Hospital, 119074, Singapore; 6 Yong Loo Lin School of Medicine, National University of Singapore, 119077, Singapore; 7 KK Women's and Children's Hospital, 229989, Singapore; 8 Duke-NUS Graduate Medical School, 169857, Singapore; 9 Singapore Institute of Clinical Sciences, Agency for Science, Technology and Research (A^∗^STAR), 117609, Singapore

## Abstract

Dendritic cell (DC)-mediated cross-presentation of exogenous antigens acquired in the periphery is critical for the initiation of CD8^+^ T cell responses. Several DC subsets are described in human tissues but migratory cross-presenting DCs have not been isolated, despite their potential importance in immunity to pathogens, vaccines, and tumors and tolerance to self. Here, we identified a CD141^hi^ DC present in human interstitial dermis, liver, and lung that was distinct from the majority of CD1c^+^ and CD14^+^ tissue DCs and superior at cross-presenting soluble antigens. Cutaneous CD141^hi^ DCs were closely related to blood CD141^+^ DCs, and migratory counterparts were found among skin-draining lymph node DCs. Comparative transcriptomic analysis with mouse showed tissue DC subsets to be conserved between species and permitted close alignment of human and mouse DC subsets. These studies inform the rational design of targeted immunotherapies and facilitate translation of mouse functional DC biology to the human setting.

## Graphical abstract

**Graphical Abstract:**

**Figure:**
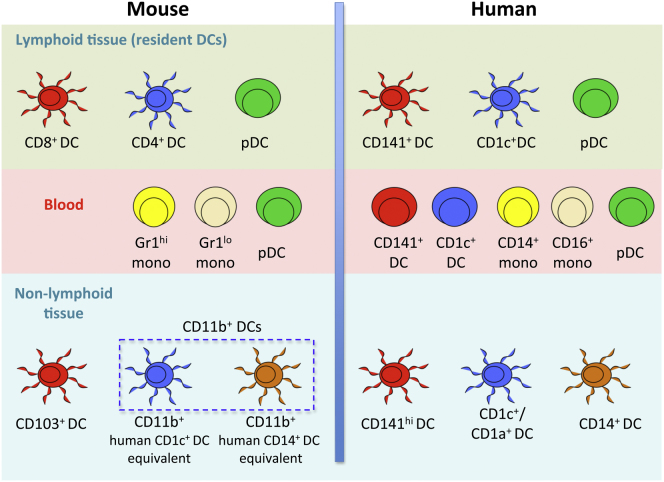

**Highlights:**

► Human tissues contain CD1c^+^ DCs, CD14^+^ DCs, and a CD141^hi^ cross-presenting DC subset ► CD141^hi^ DCs migrate to draining lymph nodes and probably arise from blood CD141^+^ DCs ► Human tissue CD141^hi^ DCs are homologous to mouse CD103^+^ or CD8^+^ DCs ► Human tissue CD1c^+^ DCs are homologous to mouse CD4^+^ DCs

## Introduction

Dendritic cells (DCs) are a heterogeneous population of rare leukocytes found in virtually all tissues, where they form a network of antigen-sensing and -presenting cells (Steinman, 2007). The main role of DCs is to induce specific immunity against invading pathogens while maintaining tolerance to self-antigens. DC-mediated cross-presentation of exogenous antigens to CD8^+^ T cells is critical for the priming and activation of cellular immunity to viruses, tumors, and vaccines and to promote tolerance through the deletion of self-reactive CD8^+^ T cells (Kurts et al., 2010).

By virtue of their location, migratory tissue DCs play a central role in the induction of immunity (Banchereau and Steinman, 1998; Allenspach et al., 2008). Cross-presenting migratory DCs are functionally and anatomically specialized to acquire exogenous antigen in the tissues and initiate CD8^+^ T cell responses (Helft et al., 2010; del Rio et al., 2010). Although cross-presenting capacity is also found in lymph node (LN)-resident DCs and may augment antigen presentation by migratory DCs (Shortman and Heath, 2010), the latter are obligatory for efficient T cell priming (Allenspach et al., 2008).

Studies in mice have identified two lineages of migratory tissue DCs defined by differential expression of CD103 and CD11b. CD103^+^ DCs cross-present antigen to CD8^+^ T cells more effectively than do CD11b^+^ DCs in a number of viral infection, tumor, and self-antigen models (Helft et al., 2010; del Rio et al., 2010). Although these DCs play a critical role in immunity, no population with this activity has ever been isolated from human nonlymphoid organs. Human DCs of the interstitial dermis are the best described tissue DCs and comprise two subsets delineated by expression of CD1a and CD14 (Lenz et al., 1993; Nestle et al., 1993; Klechevsky et al., 2008; Haniffa et al., 2009). The surface markers bear no relation to mouse DCs and neither human DC subset excels in cross-presentation, so a functional homolog of the mouse CD103^+^ DC remains elusive (Collin et al., 2011).

Several lines of evidence indicate that CD103^+^ cross-presenting tissue DCs and CD8^+^ lymphoid-resident DCs together form a distinct DC lineage. Studies of genetic factors affecting DC development suggest that both are dependent upon the function of *IRF8*, *Id2*, and *Batf3* (Aliberti et al., 2003; Edelson et al., 2010) and both arise from a common precursor, the pre-DC (Ginhoux et al., 2009; Liu et al., 2009). Additional phenotypic and functional properties are found in common between CD103^+^ and CD8^+^ DCs including uptake of apoptotic cells via the lectin CLEC9A (Caminschi et al., 2008; Huysamen et al., 2008; Sancho et al., 2009), responsiveness to TLR3 stimulation (Jelinek et al., 2011), and expression of the chemokine receptor XCR1 (Crozat et al., 2011). The lectins DEC205 and langerin are also expressed on DCs of this lineage in a number of different sites (Contreras et al., 2010; Ginhoux et al., 2009).

Recent progress was made in identifying potential homologs of mouse DC subsets by examining human blood DCs. In addition to plasmacytoid DCs (pDCs), human blood harbors two subsets of CD11c^+^ myeloid DCs: a major CD1c^+^ (BDCA-1) population and a discrete CD141^+^ (thrombomodulin or BDCA-3) subset (Dzionek et al., 2000; MacDonald et al., 2002; Ziegler-Heitbrock et al., 2010). Compared with CD1c^+^ DCs, blood CD141^+^ DCs exhibit specialized cross-presenting function and express a number of markers associated with mouse CD8^+^ DCs (Jongbloed et al., 2010; Bachem et al., 2010; Crozat et al., 2010a; Poulin et al., 2010) including NECL2 (cellular adhesion molecule-1; *CADM1*) (Galibert et al., 2005) and CLEC9A (Caminschi et al., 2008; Huysamen et al., 2008; Sancho et al., 2009). Cloning of XCL1 and subsequent mapping of its receptor to a restricted set of DCs led to the discovery of XCR1 as a signature chemokine axis of cross-presenting DCs (Dorner et al., 2009; Crozat et al., 2010a). Together with TLR3, these markers provide a concise functional profile of cross-presenting DCs that transcends species barriers.

Although the function of human blood myeloid DCs is not known and they have no counterparts in mouse blood, these observations gave us further impetus to look for migratory cross-presenting DCs in human nonlymphoid tissues and to discover how they relate to known human tissue and blood populations. Here we identify the human tissue cross-presenting DC as a discrete population of CD141^hi^CD11c^lo-int^ DCs in the HLA-DR^+^, lineage^−^, CD14^−^ compartment of skin, liver, and lung. Comparison of this cell with the major population of CD1c^+^ DCs, CD14^+^ DCs, and blood DCs allowed us to align mouse and human DC subsets across species. The characterization of CD141^hi^ DCs is likely to be fundamentally important in learning how to manipulate immune responses to tumors, viruses, and vaccines.

## Results

### Identification of CD141^hi^ DCs in Human Tissues

**Figure 1:**
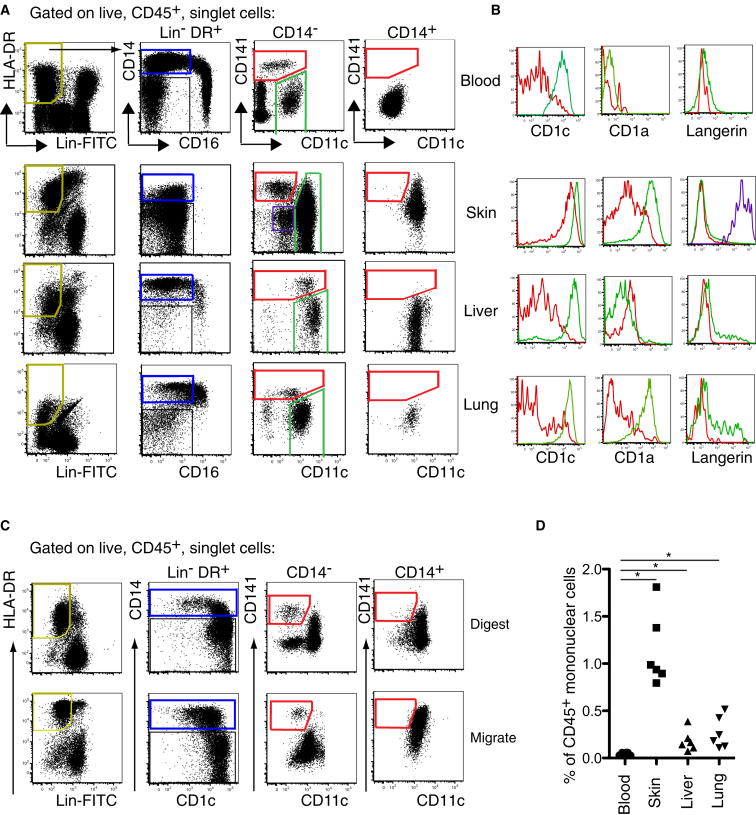
Identification of CD141^hi^ DCs in Human Tissues (A) Flow cytometry of peripheral blood, collagenase-treated whole skin, liver, and mechanically dispersed lung. Gating strategy used to identify three myeloid DC subsets within Lin^−^HLA-DR^+^ fraction (yellow gate) in tissues: (1) CD14^+^ DCs (blue gate), (2) CD14^−^CD11c^+^ DCs (green gate), and (3) CD14^−^CD11c^lo^CD141^hi^ DCs (red gate). Langerin^hi^ epidermal LCs (purple gate) are identifiable in the skin. Representative data from 20 blood, 18 skin, 12 liver, and 8 lung donors are shown. (B) Relative expression of CD1c, CD1a, and langerin by CD11c^+^ DCs (green) and CD141^hi^ DCs (red). Representative data from four blood, skin, liver, and lung donors are shown. (C) Identical gating strategy as (A) to correlate CD141^hi^ cells with established populations of skin DCs from digested dermis and spontaneously migrating DCs from skin explants cultured for 60 hr. Representative data from seven skin donors are shown. (D) Frequency of CD141^hi^ cells as a percent of CD45^+^ mononuclear cells in skin, liver, and lung relative to peripheral blood. Composite data from six blood, skin, liver, and lung donors are shown. ^∗^p < 0.05, Mann-Whitney U test. See also Figure S1.

We have previously described a strategy to identify CD1a^+^ DCs, CD14^+^ DCs, and macrophages in freshly digested dermis (Haniffa et al., 2009). We combined this with a conventional analysis of human blood DCs to compare phenotypically equivalent cells from blood and tissues in parallel (Figure 1). Lineage cocktail in FITC was used to exclude auto-fluorescent tissue macrophages and lineage-positive cells. Antibodies to CD14 and CD16 were put in separate channels to identify monocyte subsets, and CD11c, CD1c, and CD141 markers were added to map tissue DC subsets to the parameter space of blood DC analysis. Full gating strategy and percentage of DC populations in blood and tissues are shown in Figure S1 available online.

HLA-DR^+^lineage^−^ cells in all tissues comprise a CD14^−^ and CD14^+^ fraction (Figure 1A). Blood, and to a lesser extent lung and liver, also contain CD16^+^ monocytes or equivalent cells. The CD14^−^ fraction may be further separated by CD141 and CD11c expression. In blood, typical CD141^+^ DCs are a distinct population with lower CD11c expression. This population is mirrored in skin, liver, and lung as CD141^hi^ cells with low-to-intermediate CD11c expression. The CD14^+^ fraction contains CD11c^+^ cells with a variable CD141 expression but no CD141^hi^CD11c^lo-int^ cells. Double-negative cells on the plot of CD141 versus CD11c in blood correspond to CD123^+^ pDCs and CD34^+^ progenitor cells (Figure S1). CD141^lo^CD11c^lo^ cells on the plot of CD141 versus CD11c in skin preparations express high CD1a and langerin and are epidermal Langerhans cells (LCs) (Figures 1A and S1).

The expression of CD1c, CD1a, and langerin is defined in Figure 1B. CD141^hi^CD11c^lo-int^ cells have lower expression of CD1c and are further described as “CD141^hi^ DCs.” All CD141^lo^CD11c^hi^ cells express higher CD1c and are hereafter referred to as “CD1c^+^ DCs.” CD1a and langerin are not found on blood DCs but are variably expressed in the tissues on a small fraction of CD1c^+^ DCs. CD141^hi^ DCs do not express langerin and have similar HLA-DR expression levels to CD1c^+^ DCs (data not shown).

In order to verify this approach and to allow comparison with previous studies on DCs migrating from human skin, we compared freshly digested and migrated skin preparations by gating cells according to relative expression of CD14 and CD1c (Figure 1C). In both preparations, the CD14^−^ fractions include a distinct minor population of CD141^hi^CD11c^lo^ cells, corresponding to the cells identified previously. The CD14^+^ fractions contain CD11c^hi^ cells with variable to high CD141 expression but no cells in the CD141^hi^CD11c^lo^ gates. To clarify that CD141^hi^ DCs were tissue residents and not contaminating blood cells, we estimated their frequency relative to CD45^+^ mononuclear cells. CD141^hi^ DCs were enriched relative to CD141^+^ DCs in blood in all tissues, especially the skin (Figure 1D).

From these results, we conclude that CD141^hi^ DCs may be identified as a discrete population of HLA-DR^+^lineage^−^CD14^−^CD141^hi^CD11c^lo-int^ cells of skin, lung, and liver. Parallel phenotypic analysis suggests that they are potentially related to blood CD141^+^ DCs. Although CD14^+^ cells in all tissues express CD141, they correspond to previously identified CD14^+^ “interstitial-type DCs” of skin that do not have very potent allostimulatory or cross-presenting capacity (Klechevsky et al., 2008; Haniffa et al., 2009). Hereafter, we refer to these cells as “CD14^+^ DCs.”

### CD141^hi^ Tissue DCs Express Markers of Cross-Presenting DCs

**Figure 2:**
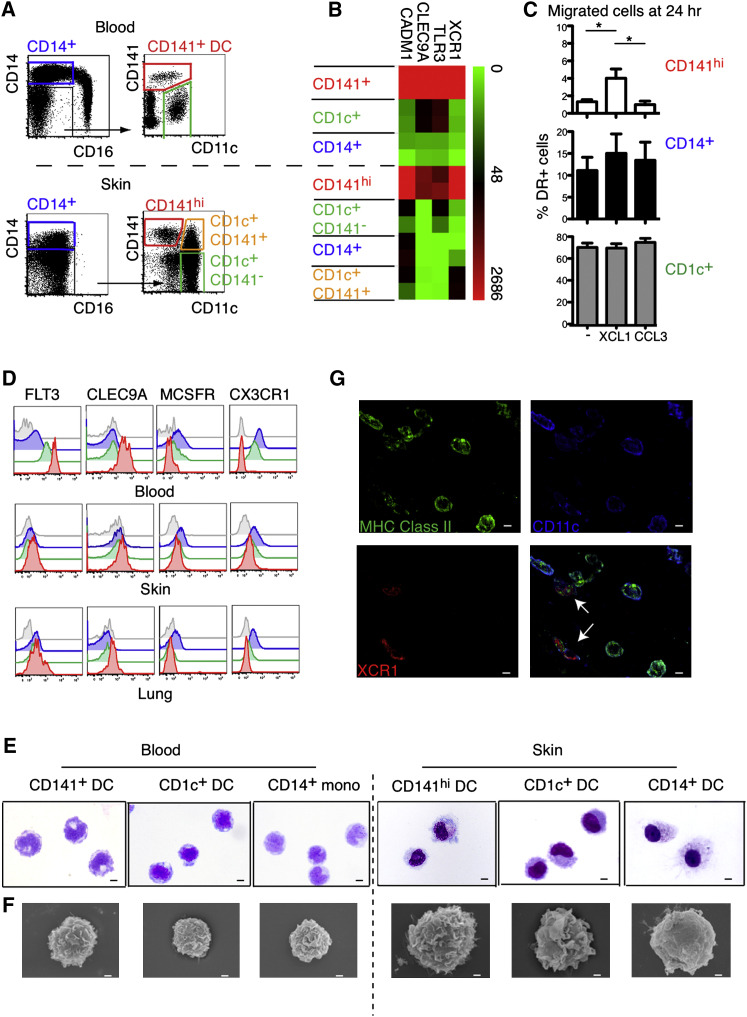
CD141^hi^ Tissue DCs Express Markers of Cross-Presenting DCs (A) Lin^−^HLA-DR^+^ cells from blood and skin were FACS purified according to gating shown in dot plots. (B) RNA from FACS-purified cells in (A) was analyzed for the expression of *XCR1, TLR3, CLEC9A*, and *CADM1* by qRT-PCR. Data shown are from two blood and skin donors. (C) DC migration from skin explants cultured ex vivo over 24 hr in medium alone (−) or with XCL1 or CCL3. Composite data from four skin donors are shown, mean ± SEM. ^∗^p < 0.05, Mann-Whitney U test. (D) Relative expression of FLT3, CLEC9A, MCSFR, and CX3CR1 by CD14^+^ DCs (blue), CD1c^+^ DCs (green), and CD141^hi^ DCs (red) compared to isotype (gray) from blood, skin, and lung. Representative data from three blood, skin, and lung donors are shown. (E and F) Morphology of FACS-sorted blood and skin CD141^+^, CD1c^+^, and CD14^+^ DCs and monocytes (E) visualized by GIEMSA staining of cytospin preparations (X100) and (F) by SEM. Scale bars represent 10 μm in (E) and 1 μm in (F). (G) Pseudo-color (×63) images of whole-mount skin immunostained for MHC class II (HLA-DR, HLA-DQ, and HLA-DP) (green), CD11c (blue), and XCR1 (red). White arrows in overlay image (right bottom) highlight the XCR1^+^CD11c^lo^HLA-DR^+^ cells corresponding to skin CD141^hi^ DCs. Scale bars represent 5 μm.

Seeking further evidence that tissue CD141^hi^ cells were tissue cross-presenting DCs, we characterized their expression of signature markers such as *XCR1, TLR3, CLEC9A*, and *CADM1*. Mindful that CD141 expression was also found on CD14^+^ DCs and some CD1c^+^ DCs, these fractions were included to ensure that they were not also enriched for potential cross-presenting DCs (Figure 2A).

As expected, *XCR1, TLR3, CLEC9A*, and *CADM1* were upregulated on blood CD141^+^ DCs compared with blood CD1c^+^ DCs and CD14^+^ monocytes (Figure 2B). A close correlation was seen with CD141^hi^ DCs whereas CD14^+^ DCs and both fractions of CD1c^+^ DCs had much lower expression of all markers. These data indicate that of the interstitial dermal subsets, CD141^hi^ DCs, but not other CD141^+^ cells, are potential cross-presenting DCs.

To corroborate the transcription profiles, we examined the response of dermal DC subsets to XCL1 in vitro (Figure 2C). XCL1 significantly and selectively increased the proportion of CD141^hi^ DCs migrating from explanted skin over 24 hr. CD141^hi^ DCs also showed the highest expression of FLT3 and CLEC9A in blood, skin, and lung, whereas CD14^+^ DCs expressed the most M-CSFR and CX3CR1, markers associated with the monocyte and macrophage lineages. CD1c^+^ DCs showed lower expression of FLT3 and CLEC9A and intermediate levels of M-CSFR and CX3CR1, compared with CD141^hi^ DCs (Figure 2D).

The morphology of sorted blood and skin DCs was examined by Giemsa staining of cytospin preparations (Figure 2E) and scanning electron microscopy (SEM) (Figure 2F). Both CD141^hi^ and CD1c^+^ DCs showed prominent membrane ruffling compared with CD14^+^ DCs, which were generally smoother. CD141^hi^ DCs had more numerous small lamellipodia than did CD1c^+^ DCs. With four-color immunofluorescence staining of whole-mount skin, it was possible to discern HLA-DR^+^XCR1^+^ cells with low CD11c expression, consistent with the phenotype of CD141^hi^ DCs, in the apical dermis (Figure 2G). Other HLA-DR^+^CD11c^hi^XCR1^−^ cells may be seen in the same field, most probably representing either CD1c^+^ DCs or CD14^+^ DCs. CD141 itself could not be used as a marker to visualize these cells directly because it is widely expressed on leukocytes and endothelial cells.

### Skin CD141^hi^ DCs Potentially Develop from Blood CD141^+^ DCs

**Figure 3:**
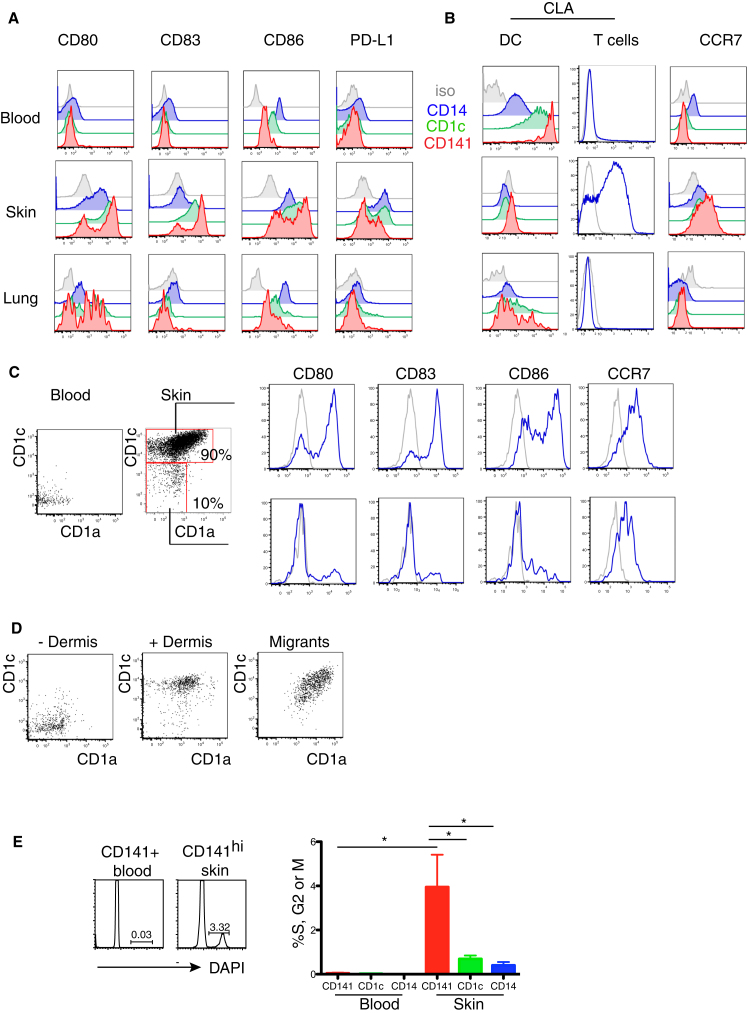
Skin CD141^hi^ DCs Potentially Develop from Blood CD141^+^ DCs (A and B) Relative expression of (A) CD80, CD83, CD86, and PD-L1 and of (B) CLA and CCR7 by blood and skin CD14^+^ DCs (blue), CD1c^+^ DCs (green), and CD141^hi^ DCs (red) compared to isotype (gray). Representative data from three blood, skin, and lung donors are shown. (C) Relative expression of CD80, CD83, CD86, and CCR7 (blue) to isotype control (gray) by CD1a^+^CD1c^+^ and CD1a^−^CD1c^−^ fractions of CD141^hi^ cells in the skin (shown with their relative percentages). Representative data from three skin donors are shown. (D) CD1c and CD1a expression by FACS-purified, Qtracker605-labeled blood CD141^+^ DCs cultured in medium (−Dermis) or with digesting dermis (+Dermis) and migrated CD141^hi^ cells from 60 hr skin explants cultured ex vivo (Migrants). Representative data from two blood and skin donors for “spiking” experiment and five donors for skin explant migration are shown. (E) DNA content of FACS-sorted blood and skin CD14^+^, CD1c^+^, and CD141^hi^ DCs. Right panel shows percent of DCs in S, G2, or M phase in blood and skin. Representative and composite data from four blood and five skin donors are shown, mean ± SEM. ^∗^p < 0.05, Mann-Whitney U test comparing skin CD141^hi^ DCs with all other subsets.

Comparison of skin CD141^hi^ DCs with blood CD141^+^ DCs suggested a potential developmental relationship between the blood and skin. All tissue DCs, especially CD141^hi^ DCs, expressed an activated phenotype compared with the blood (Figures 3A and 3B) and acquired high levels of CCR7 while downregulating the skin-homing molecule cutaneous lymphocyte antigen (CLA, also known as P-selectin glycoprotein ligand; PSGL-1) (Figures 3A and 3B).

Careful analysis of skin CD141^hi^ DCs shows two distinct populations of cells with differential expression of CD1a and CD1c (Figure 3C). A minor population of CD1a^−^CD1c^−^ cells is present. Although these do not express CLA and cannot be blood CD141^+^ DCs per se, they have a similar immature phenotype (Figure 3C). These data are consistent with the possibility that blood CD141^+^ DCs are the precursors of immature CD141^hi^ DCs, before acquiring CD1a, CD1c, activation antigens, and CCR7. To examine this possibility, we added sorted and labeled CD141^+^ blood DCs to a skin preparation and observed an upregulation of CD1a and CD1c in keeping with the tissue CD141^hi^ DC phenotype (Figure 3D). Furthermore, migrating CD141^hi^ DCs represented only the CD1a and CD1c mature fraction. This is at least consistent with a precursor-progeny relationship between blood CD141^+^ DCs and skin CD141^hi^ DCs. In mouse experiments, blood pre-DCs are not proliferating but go into cell cycle upon entry into the tissues (Liu et al., 2009). Reminiscent of this, we found that blood CD141^+^ DCs are not cycling but 4% of CD141^hi^ DCs are in S, G2, or M phase by DNA content analysis (Figure 3E).

### CD141^hi^ DCs Migrate to Skin-Draining Lymph Nodes

**Figure 4:**
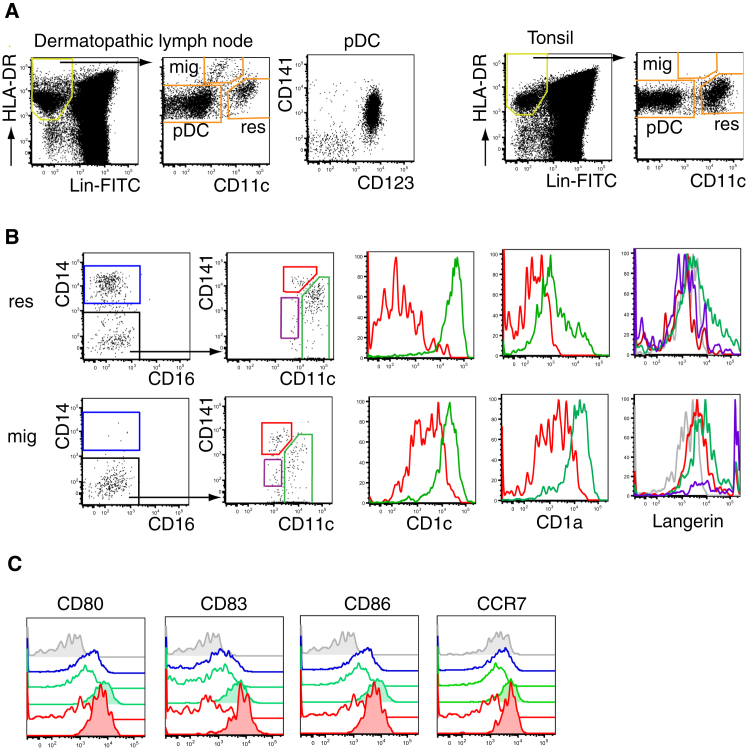
CD141^hi^ DCs Migrate to Skin-Draining Lymph Node (A) Identification of migratory (mig) and resident (res) DCs in mechanically dispersed dermatopathic LN (left) and tonsil (right). (B) Comparison of CD141^hi^ and CD1c^+^ DCs in migratory (mig) and resident (res) fractions. Relative expression of CD1c, CD1a, and langerin by CD1c^+^ DCs (green), CD141^hi^ DCs (red), and cells from epidermal LC gate (purple). (C) Relative expression of CD80, CD83, CD86, and CCR7 by migratory (tinted) and resident CD14^+^ DCs (blue), CD1c^+^ DCs (green), and CD141^hi^ DCs (red) compared to isotype (gray). Representative data from four dermatopathic LNs and four tonsils are shown.

CD141^hi^ DCs migrate spontaneously in vitro (Figures 1 and 2) and express CCR7 (Figure 3), suggesting that they may migrate to LNs in vivo. To test this further we compared dermatopathic LN, which contains a high content of migratory DCs, with tonsil, a lymphoid tissue lacking afferent lymphatics (Figure 4A). Dermatopathic LNs contained an additional CD11c^lo^HLA-DR^hi^ population. Based on the comparison of these two tissues and in keeping with the phenotype of migrating DCs in mouse LNs (Ohl et al., 2004), we identified the CD11c^lo^HLA-DR^hi^ population as tissue migratory DCs. Both LN and tonsil contained pDC and a CD11c^hi^HLA-DR^lo^ population, which contain resident DCs. Within both fractions, we found CD141^hi^ DCs and CD1c^+^ DCs but Langerhans cells, as predicted, were confined to the migratory fraction. In keeping with the phenotype expected of migratory cells that we had observed directly in the skin, all migratory fractions expressed higher CD1c, CD1a, CCR7, and activation antigens than did their resident counterparts. Taken together, these data indicate that CD141^hi^ DCs are capable of migration to LNs in vivo, at least in the inflammatory setting of dermatosis (Figures 4B and 4C).

### CD141^hi^ DCs Are Superior at Cross-Presentation of Soluble Antigen

Having identified CD141^hi^ DCs as a small subset of tissue-derived DCs with a phenotype consistent with cross-presenting DCs, we embarked on functional studies. We focused on cells isolated from the skin, because it was more easily obtained and contained the greatest number of cells. This still presented a technical challenge, because the frequency of CD141^hi^ DCs is only 1% of CD45^+^ cells, or fewer than 500 cells per cm^2^.

**Figure 5:**
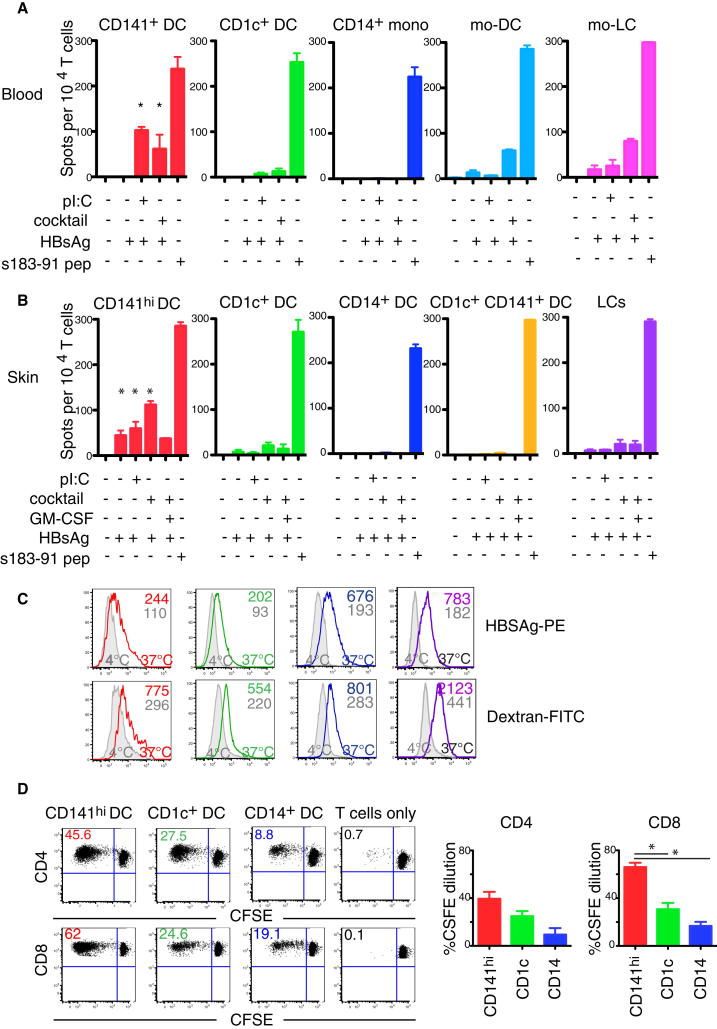
CD141^hi^ DCs Are Superior at Cross-Presenting Soluble Antigen (A and B) IFN-γ production assessed by ELISpot assay upon cross-presentation of soluble HBsAg by blood and skin DCs to HLA-A^∗^0201-restricted s183-91 CD8^+^ T cell clones at a DC:T cell ratio of 1:2.5. Composite data from four blood and seven skin donors are shown with mean ± SEM. ^∗^p < 0.05, Mann-Whitney U test, comparing respective experimental condition for CD141^+^ blood and CD141^hi^ skin DCs with CD1c^+^ DCs, CD14^+^ blood monocytes, skin DCs, and LCs. (C) Uptake of PE-labeled HBsAg and FITC-conjugated Dextran by skin CD141^hi^ DCs (red), CD1c^+^ DCs (green), CD14^+^ DCs (blue), and LCs (purple). Representative data from two skin donors are shown; median fluorescent intensity of antigen uptake for each DC subset at 37°C (color) compared to 4°C (gray) are stated in the histogram box. (D) Alloactivation of CD4^+^ and CD8^+^ T cells by skin CD141^hi^ DCs (red), CD1c^+^ DCs (green), and CD14^+^ DCs (blue). Proliferation was measured over 6 days by CFSE dilution. Representative dot plots and composite results from four skin donors are shown, mean ± SEM. ^∗^p < 0.05, ANOVA with post-test Bonferroni correction.

We tested the cross-presentation ability of CD141^hi^ DCs, CD1c^+^ DCs, CD14^+^ cells, and epidermal Langerhans cells from the skin in comparison to CD141^+^ DCs, CD1c^+^ DCs, CD14^+^ monocytes, and in vitro monocyte-derived DCs (mo-DCs) and monocyte-derived LCs (mo-LCs) obtained from blood. Cross-presentation of hepatitis B surface antigen (HBsAg) to HLA-A^∗^0201-restricted s183-91-specific CD8^+^ T cell clones was measured via an IFN-γ ELISpot, as described in the Experimental Procedures (Figures 5A and 5B). In blood, only CD141^+^ DCs were able to cross-present efficiently and required TLR3 stimulation with poly(I:C) or exposure to a maturation cocktail (containing poly(I:C), LPS, IFN-γ, IL-1β, TNF-α, and IFN-α). Mature mo-DCs and mo-LCs were also able to cross-present HBsAg upon exposure to maturation cocktail but were refractory to TLR3 stimulus alone (Figure 5A). In the skin, superior cross-presenting capacity was found in CD141^hi^ DCs, compared with all other skin DC subsets, including LCs (Figure 5B). In keeping with their more activated status, cross-presentation by CD141^hi^ DCs occurred in the absence of stimulation, although TLR3 stimulation and maturation cocktail both increased this activity. CD1c^+^ DCs showed little ability to cross-present antigens, even when the CD141^+^ fraction of these cells was specifically isolated and exposed to the maturation cocktail (Figure 5B). Hypothesizing that CD1c^+^ and CD14^+^ DCs cross-presenting capacities could be induced by other stimuli, we exposed all DCs and LCs to a wide range of inflammatory stimuli (including maturation cocktail plus GM-CSF, LPS plus CD40 ligand, TLR8 agonist CL075, and mycobacterial extracts) but none of these conditions elicited cross-presentation at a greater level than with cytokine cocktail (Figure 5 and data not shown). However, we cannot formally exclude that an untested condition could enhance CD1c^+^ and CD14^+^ DCs cross-presenting capacities. The ability of CD141^hi^ DCs to cross-present was not a function of antigen uptake as indicated by the fact that all populations were able to take up near-equivalent PE-labeled HBsAg (Figure 5C).

CD141^hi^ DCs were also the most active allostimulators of both CD4^+^ and CD8^+^ T cells; CD14^+^ DCs showed the least activity (Figure 5D). These results are consistent with the more activated status of CD141^hi^ DCs but did not indicate that they preferentially stimulated CD8^+^ T cells, compared with other DCs.

### CD141^hi^ DCs Synthesize CXCL10 and TNF-α but Not IL-12 or IL-23

**Figure 6:**
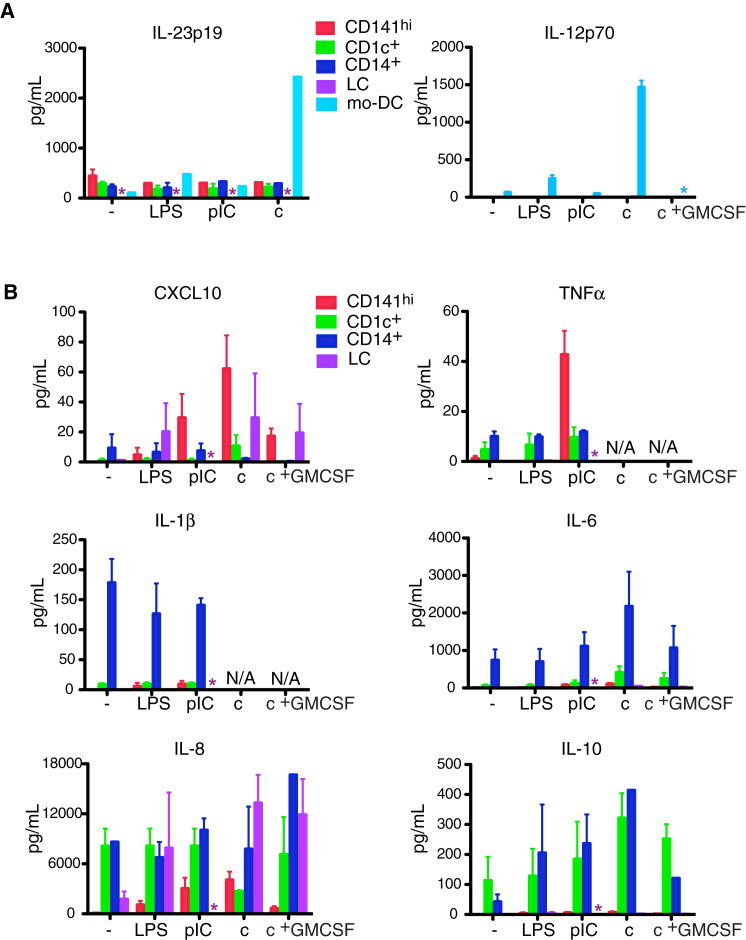
CD141^hi^ DCs Synthesize CXCL10 and TNF-α but Not IL-12 (A) IL-12p70 and IL-23p19 production from unstimulated (−), LPS (LPS)-, poly(I:C) (pIC)-, a cocktail of TNF-α, IL-1β, IFN-α, IFN-γ, LPS, and pIC (c)-, and cocktail with the addition of GM-CSF (c + GM-CSF)-stimulated CD141^hi^ DCs, CD1c^+^ DCs, CD14^+^ DCs, and LCs with mo-DCs as control. Composite results from six donors are shown, mean ± SEM. Asterisk denotes subset not analyzed. (B) CXCL10, TNF-α, IL-1β, IL-6, IL-8, IL-10, IL-23p19, and IL-12p70 production from CD141^hi^ DCs, CD1c^+^ DCs, CD14^+^ DCs, and LCs as stimulated in (A). Composite results from four donors are shown, mean ± SEM. Asterisk denotes subset not analyzed.

Polarization of T cell responses by cytokine production is an important function of DCs. In the mouse, cross-presentation to CD8^+^ T cells usually occurs in the context of a Th1 cell response reinforced by IL-12 production (Reis e Sousa et al., 1997). However, no interstitial DC subset produced IL-12p70 even after stimulation, although IL-23p19 was synthesized in modest amounts. Both were easily detected in cultures of mo-DCs (Figure 6A). CD141^hi^ DCs were most efficient at TNF-α and CXCL10 production after TLR3 stimulation but produced very little IL-1, IL-6, IL-8, or IL-10. LCs also produced CXCL10. CD14^+^ DCs were the highest producers of IL-1, IL-6, and IL-10 whereas CD1c^+^ DCs produced mainly IL-8 and IL-10 (Figure 6B). Neither CD1c^+^ DCs nor CD14^+^ DCs produced TNF-α or CXCL10 in response to all inflammatory conditions tested (Figure 6B) including LPS + CD40L, CL075, and mycobacterial extract (data not shown).

### Transcriptome Mapping of Human and Mouse Nonlymphoid Tissue DCs

The alignment of DC subsets between mouse and human is of key importance in correlating human studies with mouse in vivo experiments (Guilliams et al., 2010). The results described above suggest that CD141^hi^ DCs are functional homologs of mouse CD103^+^ DCs and that these are distinct from the major population of human CD1c^+^ DCs and mouse CD11b^+^ DCs. We sought to verify the conservation between species by an unbiased approach. In order to strengthen the analysis, we included human blood DCs and mouse lymphoid DCs.

**Figure 7:**
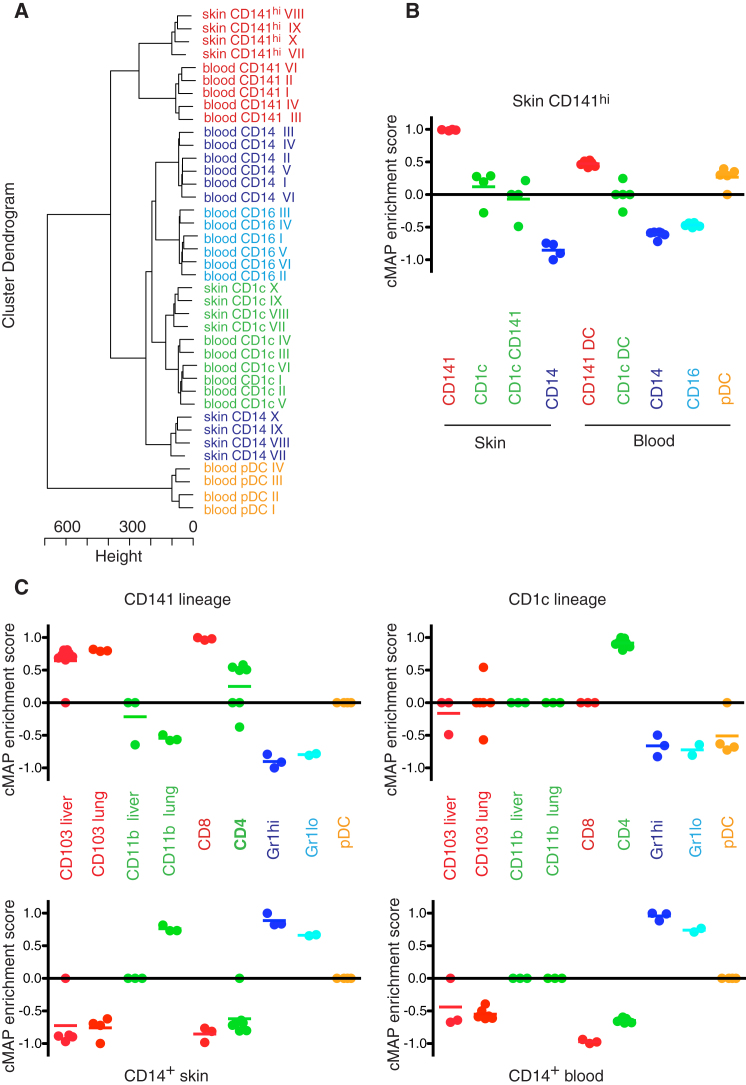
Transcriptome Mapping of Human and Mouse Nonlymphoid Tissue DCs Microarray expression profiles were obtained from FACS-purified monocyte and DC subsets from human blood and skin and mouse bone marrow, blood, spleen, liver, and lung. (A) Cluster dendrogram of human DC and monocyte subsets after removal of tissue-specific genes demonstrating the relationships between blood CD141^+^ DCs and skin CD141^hi^ DCs (red), blood and skin CD1c^+^ DCs (green), CD14^+^ monocytes and DCs (blue), blood CD16^+^ monocytes (light blue), and pDCs (brown). Microarray profiles were obtained from six blood and four skin donors. (B) CMAP enrichment scores for skin CD141^hi^ DCs against all human skin and blood monocyte and DC subsets. Scatterplot and mean, a 1,000 permutation test between gene signatures was performed on each enrichment score to determine significance. Enrichment scores for human skin CD141^hi^ DCs with all other human monocyte and DC subsets were significant at p < 0.0001. (C) CMAP enrichment scores for human (1) CD141 lineage, (2) CD1c lineage, (3) skin CD14^+^ DCs, and (4) blood CD14^+^ monocytes against mouse monocyte and DC subsets. CMAP analysis was performed with mouse orthologs of human transcripts. Microarray profiles were obtained from 3–4 experimental sets from 10–15 WT mice per set. Scatterplot and mean, a 1,000 permutation test between gene signatures was performed on each enrichment score to determine significance. Enrichment scores were significant at p < 0.05 for all data sets except for the inverse association between human CD1c lineage with mouse Gr1^hi^ and Gr1^lo^ monocytes.

We sorted tissue DC subsets, blood DCs, and monocytes from human samples and generated gene signatures for each subset by removing tissue-specific expression patterns as detailed in the Supplemental Experimental Procedures. Sorted populations are shown in Table S1. A hierarchical clustering of all the subsets used for signature generation shows close clustering of CD141^hi^ skin DCs with CD141^+^ blood DCs and CD1c^+^ DCs from blood with CD1c^+^ DCs from skin, suggesting the existence of two common DC subsets in blood and skin (Figure 7A).

Connectivity map analysis (CMAP) was performed comparing the skin CD141^hi^ DC gene set with the expression profile of other human DC and monocyte subsets (full details of bioinformatics analysis are described in Supplemental Experimental Procedures). The CMAP scores are scaled dimensionless quantities that indicate the degree of enrichment or “closeness” of one DC subset to another. CD141^+^ blood DCs show the highest enrichment with skin CD141^hi^ DCs, followed by pDCs, then CD1c DCs of skin and blood. CD14^+^ skin DCs and blood monocyte subsets both show inverse relationships with skin CD141^hi^ DCs (Figure 7B). This analysis also indicates similar enrichment scores between CD141^+^ and CD141^−^ fractions of CD1c^+^ skin cells, suggesting that they are both components of the CD1c lineage.

Because skin CD141^hi^ and blood CD141^+^ DCs and CD1c^+^ DCs from both skin and blood clustered with each other, we generated a pooled skin CD141^hi^ or blood CD141^+^ DC signature and a pooled CD1c^+^ DC signature from the two tissues. This was used to interrogate the relationship between these two human “DC lineages” and mouse DC subsets by further CMAP analysis (Figure 7C). Corresponding sorted mouse populations are shown in Table S1. The human CD141 lineage shared the highest enrichment scores with mouse CD103^+^ tissue DCs and CD8^+^ splenic DCs. In contrast, the CD1c lineage was closest to mouse splenic CD11b^+^CD4^+^ DCs. CD11b^+^ DCs in murine nonlymphoid tissues were more distantly related to the human CD1c lineage. CD11b^+^ murine DCs from lung had a positive association with human CD14^+^ DCs. This suggests that the CD11b^+^ population is heterogeneous and contains mouse equivalents of CD14^+^ DCs in a higher proportion in the lung than the liver. In addition, CD14^+^ DCs from the skin generated the highest enrichment with CD14^+^ monocytes and mouse monocytes (Figure 7C). This analysis suggests by an unbiased means that CD141^hi^ skin DCs have a transcriptional profile that links them to murine CD103^+^ tissue DCs and that both form a conserved DC lineage that includes CD141^+^ blood DCs in humans and CD8^+^ lymphoid DCs in mice.

## Discussion

This study describes the phenotype and function of a discrete human DC subset identified by high CD141 and low-to-intermediate CD11c expression, found within the HLA-DR^+^lineage^−^CD14^−^ fraction of leukocytes isolated from skin, liver, and lung. CD141^hi^ DCs express *CLEC9A, TLR3, CADM1*, and *XCR1* and consequently migrate in response to XCL1. Skin CD141^hi^ DCs are proliferating and include a subset of immature cells related to but distinct from blood CD141^+^ DCs, suggesting that they are potentially derived from CD141^+^ blood DCs. CD141^hi^ DCs acquire CD1c and CD1a, markers that are also expressed when human CD34^+^ progenitor cells or monocytes differentiate into DCs in vitro (Albert et al., 1998; Klechevsky et al., 2008). CD141^hi^ DCs in skin also express CCR7, migrate spontaneously, and are detectable as a distinct population within the migratory fraction of DCs in skin-draining lymph nodes. In vitro, CD141^hi^ DCs are most efficient at cross-presenting soluble antigens compared with other interstitial DCs and epidermal LCs. A wide range of inflammatory stimuli were tested, including TNF-α, IFN-α, GM-CSF, and LPS, with CD40 ligand, but failed to elicit efficient cross-presentation by other interstitial DCs or LCs. Monocyte-derived DCs and LCs were both able to cross-present in the assay but less so than CD141^hi^ DCs. Published data show that CD34-derived langerin^+^ DCs are good at cross-presentation compared with CD14^+^ DCs (Klechevsky et al., 2008). This contrasts with another report showing poor cross-presenting function of primary epidermal LCs (van der Vlist et al., 2011). Our data reconcile these observations to some degree showing that in-vitro-derived LCs have superior cross-presenting ability compared with primary LCs.

CD141^hi^ DCs synthesize very little IL-12 and IL-23 when stimulated but produce CXCL10 and TNF-α. No stimuli elicited CXCL10 or TNF-α from CD1c^+^ and CD14^+^ DCs. We considered the possibility that inflammatory cytokine production might be required for cross-presentation by these cells, but the addition of exogenous TNF-α, IFN-α, and GM-CSF failed to confer cross-presenting capacity. LCs produced CXCL10 but did not cross-present efficiently. It remains a formal possibility that an untested condition will induce cross-presenting capacity in CD1c^+^ and CD14^+^ DCs, but we did not find this among a wide range of physiological stimuli.

The production of CXCL10 by CD141^hi^ DCs is interesting because XCL1 and CXCL10 potentially form a chemokine circuit between DCs bearing XCR1 and activated NK cells or Th1 cells (Dorner et al., 2009; Crozat et al., 2010a; Contreras et al., 2010). These lymphocytes express the CXCL10 receptor CXCR3 (Acosta-Rodriguez et al., 2007) and are also major producers of XCL1.

In addition to CD141^hi^ DCs, human tissues contain CD1c^+^ DCs and CD14^+^ DCs, as previously described, notably in the skin (Lenz et al., 1993; Nestle et al., 1993; Haniffa et al., 2009). CD141^hi^ DCs were more difficult to isolate than CD141^+^ blood DCs, owing to the upregulation of CD141 on a number of other cells including CD14^+^ DCs and a proportion of CD1c^+^ DCs. Although CD141 is not selectively expressed, this antigen can be used to identify cross-presenting tissue DCs in the appropriate context. Other markers such as CLEC9A and XCR1 are highly discriminatory, as emphasized by others (Caminschi et al., 2008; Huysamen et al., 2008; Sancho et al., 2009; Crozat et al., 2011), but are difficult to stain on tissue cells. To validate the identity of CD141^hi^ DCs, we showed that other CD141-expressing cells lacked the critical features of cross-presenting DCs in phenotypic and functional assays.

A recent study isolated CD141^+^ DCs from human skin that also express CD14, produce IL-10, and induce regulatory T cells (Chu et al., 2012). This population most probably corresponds to “CD14^+^ DCs,” which we noted to express CD141 and synthesize IL-10. In contrast to Chu et al. (2012), we did not find CD14^+^ DCs efficient at cross-presentation. Our transcriptomic analysis indicates that CD14^+^ DCs are related to blood monocytes rather than the cross-presenting DCs of mouse tissues and human blood. Notably, Chu et al. (2012) also demonstrate that equivalents of their skin CD14^+^CD141^+^ DCs can be derived from human monocytes.

Having isolated CD141^hi^ DCs from skin, liver, and lung, we also demonstrated migratory and resident CD141^hi^ populations in skin-draining lymph nodes. Cells bearing related markers have previously been reported in the T cell areas of lymphoid tissues, bone marrow (Jongbloed et al., 2010), and in the spleen (Galibert et al., 2005; Poulin et al., 2010; Mittag et al., 2011). With the exception of splenic DCs, functional studies were not undertaken with these lymphoid-derived populations (Galibert et al., 2005). Work with humanized mice has also described the development of CD141^+^ DCs similar to blood CD141^+^ DCs in the spleen but not in nonlymphoid organs (Poulin et al., 2010). CD141^+^ cells have been found in human lung, alveolar fluid, and kidneys but have not been characterized in detail (Demedts et al., 2005; Tsoumakidou et al., 2006; Fiore et al., 2008). A recent report characterizing migratory and resident DCs from human lymph nodes concluded that CLEC9A^+^ DCs were absent among migratory skin DCs (Segura et al., 2012). In this study, CD1a^−^CD14^−^ DCs were examined for CLEC9A expression. We have shown that skin migratory CD141^hi^ DCs coexpress CLEC9A but most are also positive for CD1a. It is therefore possible that CLEC9A^+^ migratory DCs are excluded by gating only CD1a-negative cells.

Functional alignment of human and mouse DC subsets has been hampered by differences in surface marker expression and accessibility of equivalent sources. The identification of cross-presenting DCs as a specialized lineage first in mouse and now in humans has been predicated on a small number of specialized markers including NECL2 (cellular adhesion molecule-1; *CADM1*) (Galibert et al., 2005), CLEC9A (Caminschi et al., 2008; Huysamen et al., 2008; Sancho et al., 2009), XCR1 (Dorner et al., 2009; Crozat et al., 2010a), and TLR3. These analyses focused on human blood but did not address nonlymphoid organs. In the tissues, neither CD1c^+^ nor CD14^+^ DCs excel at cross-presentation and the CD14^+^ subset shares features of monocytes or macrophages (Haniffa et al., 2009) making it difficult to envisage comparisons with mouse CD103^+^ and CD11b^+^ DCs. The identification of CD141^hi^ DCs therefore provided a missing link with which to compare human and mouse DC subsets of nonlymphoid origin and to explore the wider relationships between the species.

For this purpose, we developed a modification of the functional genomic analysis by gene set enrichment analysis (GSEA), pioneered by Dalod and colleagues (Robbins et al., 2008; Crozat et al., 2010b, 2011). By eliminating probe sets that were differentially expressed between skin and blood, we were able to derive subset-specific signature transcriptomes that were not defined by arbitrary expression level thresholds. In order to compare these signatures, we adapted CMAP, an extension of the GSEA algorithm (Lamb et al., 2006). This gives enrichment scores with a directional element assigning positive and negative scores to proximal and distal relationships, respectively. The enrichment scores obtained from CMAP analysis suggests the existence of at least three separate antigen-presenting lineages conserved across species: (1) CD141^hi^ or CD141^+^ cross-presenting DCs linked with mouse CD103^+^ or CD8^+^ DCs; (2) CD1c^+^ “myeloid” DCs linked with mouse splenic CD4^+^ DCs; and (3) CD14^+^ monocyte-associated or monocyte-derived DCs linked with mouse monocytes and also more distantly with mouse CD11b^+^ DCs. These data not only validate the CD141^hi^ DCs as the cross-presenting DCs of human tissues by an unbiased means but also align the entire human and mouse nonlymphoid DC subsets.

A few exceptions are notable between mouse and human cross-presenting subsets. CD141^hi^ DCs and blood CD141^+^ DCs produce very little IL-12 (Jongbloed et al., 2010; Poulin et al., 2010) in contrast to monocyte-derived DCs (Ebner et al., 2001) and mouse cross-presenting DCs (Reis e Sousa et al., 1997). In addition, human CD141^hi^ DCs do not express langerin that characterizes mouse CD8^+^ or CD103^+^ DCs in many tissues, notably the skin (Ginhoux et al., 2009). Rather, our data indicate that langerin is more likely to be found on the CD1c^+^ population, especially in liver and lung (Eisenwort et al., 2011; V.B., data not shown).

The finding that human CD14^+^ DCs are linked to monocyte populations in both mouse and human is intriguing and consistent with our initial observation that CD14^+^ DCs express M-CSFR and CX3CR1 but very little FLT3. They also express CD209 (DC-SIGN) in common with mo-DCs and have been found to be poor allostimulators in vitro (Klechevsky et al., 2008; Haniffa et al., 2009). There is no homolog of CD14^+^ DCs in mouse tissues but we speculate that the mouse CD11b^+^ DC fraction of nonlymphoid tissue is heterogeneous, comprising equivalents of both CD1c^+^ DCs and CD14^+^ DCs. This potentially explains the intermediate relationship of mouse CD11b^+^ DCs with respect to CD1c^+^ and CD14^+^ DCs by CMAP analysis. CD11b^+^ mouse spleen DCs was recently shown to be heterogeneous containing CD4^+^ and CD4^−^ fractions, the latter being monocyte derived (Lewis et al., 2011; Kasahara and Clark, 2012). Consistent with this, we noted that CD4^+^ splenic DCs were much more closely linked to CD1c^+^ DCs than were CD11b^+^ DCs from tissues, which by analogy probably contain a monocyte-derived component. Altogether, our comparison of human and mouse DC subsets aligns the functional classification of DCs across species and allows clear inferences to be drawn between mouse and human.

Targeting lectins associated with specific DC subsets is feasible in mice (Bonifaz et al., 2004; Dudziak et al., 2007), primates, and humans (Flynn et al., 2011; Tsuji et al., 2011). We anticipate that the identification of CD141^hi^ DCs will facilitate future rational vaccine design.

## Experimental Procedures

### Cell Isolation and Culture

Human samples were obtained in accordance with a favorable ethical opinion from Newcastle and Singapore Singhealth and National Health Care Group Research Ethics Committees.

Normal skin was obtained from mammoplasty and breast reconstruction surgery. Lung and liver were obtained from peritumoral tissue. Tonsil and dermatopathic lymph nodes were obtained from tonsillectomy and lymph node diagnostic excisions. 300 μm whole skin dermatome sections, liver, and lung were cut into 0.5 cm squares and incubated with 0.8 mg/ml collagenase (Type IV, Worthington-Biochemical) in RPMI (PAA) with 10% FCS (AutogenBioclear) for 2 and 8 hr, respectively, or where stated mechanically dispersed. For LC isolation, skin was incubated in 1 mg/ml dispase (Invitrogen) for 1 hr to separate epidermis from dermis prior to collagenase treatment. Migrating cells were collected from whole skin cultured in RPMI with 10% FCS with or without 1 mg/ml XCL1 (R&D) or 0.1 mg/ml CCL3 (R&D). Viability was >90% by DAPI exclusion (Sigma).

Peripheral blood mononuclear cells were isolated by density centrifugation (Ficoll-Paque; GE Healthcare). CD3^+^ T cells were isolated from whole blood to >95% purity with Rosette-Sep isolation kit (StemCell Technologies). HLA-A^∗^0201 hepatitis B surface antigen 183-91 (HBs183-91)-restricted CD8^+^ T cell clones were generated as previously described (Gehring et al., 2011).

Blood, lung, liver, and dermal DC subsets and epidermal LCs were isolated to >91% purity by fluorescence activated cell sorting (FACS) with a FACSAriaII (Becton Dickinson [BD]). Monocyte-derived DCs (mo-DCs) were generated from magnetically isolated CD14^+^ monocytes (Miltenyi Biotec) cultured for 6 days with 50 ng/ml rGM-CSF and IL-4 (R&D).

### Flow Cytometry

Flow cytometry was performed on a BDLSRII and FACSCanto and data analyzed with FlowJo (Treestar). FITC-coated dextran particles (MW70,000) were obtained from Sigma. Antibodies used are listed in Supplemental Experimental Procedures.

For determination of DNA ploidy, FACS-sorted cells were fixed, permeabilized with BD Cytofix, Cytoperm, and Permwash, treated with 70% ice-cold ethanol for 2 hr, washed, and resuspended in 2 mg/ml DAPI solution.

### Microscopy

200 μm skin sheet was fixed in PBS containing 2% paraformaldehyde and 30% sucrose overnight at 4°C. Skin was incubated overnight in PBS containing 0.5% BSA and 0.3% Triton X-100 before staining with the following primary and secondary antibodies at 4°C overnight at each stage: XCR1 (polyclonal, LSBio), CD11c (B-ly6, BD Biosciences), HLA-DR FITC (L243, BD Biosciences), HLA-DQ FITC (SK10, BD Biosciences), and HLA-DP FITC (HI43, BioLegend); and donkey anti-rabbit and donkey anti-mouse Dy549 or Dy649 (Jackson ImmunoResearch) and donkey anti-sheep Alexa Fluor 647 (Invitrogen). Specimens were viewed with Axio Imager.Z2 fluorescence microscope with Axiovision software v4.8 and Axiocam MR3 camera (Carl Zeiss, Inc.).

Cytospins were prepared from FACS-purified DCs and stained with the Hema 3 System according to manufacturer's protocol (Fisher Diagnostics). Images were analyzed with a Nikon Eclipse E800 microscope (Nikon). For scanning electron microscopy (SEM), sorted cells coated on poly-lysine (Sigma) glass coverslips were fixed in 2.5% glutaraldehyde, washed, treated with 1% osmium tetroxide (Ted Pella Inc.), and critical point dried (CPD 030, Bal-Tec). Glass coverslips were sputter-coated with platinum in a high-vacuum sputtering device (SCD005 sputter coater, Bal-Tec) and imaged with a field emission scanning electron microscope (JSM-6701F, JEOL) at an acceleration voltage of 8 kV.

### Dermal APC Stimulation with TLR Ligands

FACS-purified dermal DCs were cultured in 96-well V-bottomed plates. Supernatant from unstimulated and cells stimulated with 0.1 mg/ml LPS (Sigma), 10 μg/ml poly(I:C) (InvivoGen), a “cocktail” containing 0.1 mg/ml LPS, 25 μg/ml poly(I:C), 1,000 IU/ml IFN-γ (R&D), 50 ng/ml TNF-α (R&D), 3,000 IU/ml IFN-α (R&D), and 25 ng/ml IL-1β (R&D) with and without 50 ng/ml GM-CSF, 1 μg/ml of the TLR8 agonist CL075 (InvivoGen), and 20 μg/ml mycobacterial extracts (Strain H37Rv; BEI Resources) were collected after 24 hr for cytokine analysis. TNF-α, IL-1β, IL-6, IL-10, IL-12p70, and CXCL10 were detected with BD Cytometric Bead Arrays and analyzed with BD-FCAP Array software v1.0. IL-23p19 was measured by ELISA (R&D Quantikine ELISA kit).

### Proliferation and T Cell Cytokine Production Assays

5,000 flow-sorted dermal DC subsets were cultured with 100,000 allogeneic CFSE-labeled CD3^+^ T cells in U-bottomed 96-well plates. Proliferation was assessed by CFSE dilution on day 6.

### Cross-Presentation and ELISpot Assay

4,000 FACS-sorted blood and dermal DCs or mo-DCs from HLA-A2^+^ donors were pulsed with 10 μg/ml Hepatitis B surface antigen (HBsAg) (Rhein Biotech) with and without poly(I:C) (25 μg/ml) and a cocktail (as stated above) overnight in RPMI with 10% FCS. DCs were pulsed with HBs183-91 peptide (FLLTRILTI) for 3 hr. DCs were washed extensively and cultured with HLA-A^∗^0201 HBs183-91-restricted CD8^+^ T cell clones in RPMI with 10% FCS and IFN-γ production assessed after 18 hr by ELISpot. IFN-γ spots were detected with an IFN-γ antibody kit (Mabtech) and counted with CTL ImmunoSpot S5 UV Analyzer and ImmunoSpot Version 5 Professional Software.

### Quantitative Real-Time PCR

RNA was extracted with the RNeasy Micro Kit (QIAGEN) and reverse transcribed into cDNA by oligo-dT primer (Invitrogen) and Superscript First Strand Synthesis System (Invitrogen). cDNA was analyzed by real-time PCR with SYBR Green I Master Mix (Roche) with the LightCycler 480 System (Roche) for the following genes: *GAPDH, XCR1, TLR3*, and *CADM1* (primer sequences are listed in Supplemental Experimental Procedures).

Details of transcriptomic analysis are available in Supplemental Experimental Procedures.

### Statistical Analyses

All statistical analyses were performed with Prism 5.0 (GraphPad Software). All p values are two-tailed.

## Accession Numbers

The microarray data are available in the Gene Expression Omnibus (GEO) database (http://www.ncbi.nlm.nih.gov/gds) under the accession number GSE35459.

## Supplemental Information

Document S1. Supplemental Experimental Procedures, Figure S1, and Table S1

Table S2. List of Gene Signatures for Human Skin and Blood Monocytes and Dendritic Cell Subsets
