# Supplementary material for: Human Tissues Contain CD141hi Cross-Presenting Dendritic Cells with Functional Homology to Mouse CD103+ Nonlymphoid Dendritic Cells
Source: Immunity. 2012 Jul 27;37(1):60–73. doi: 10.1016/j.immuni.2012.04.012 (PMC3476529; doi:10.1016/j.immuni.2012.04.012)
Supplement: Document S1. Supplemental Experimental Procedures, Figure S1, and Table S1 [file mmc1.pdf]

Immunity, Volume *37*

Supplemental Information

Human Tissues Contain CD141^{hi} Cross-Presenting Dendritic Cells with Functional Homology to Mouse CD103^{+} Nonlymphoid Dendritic Cells

Muzlifah Haniffa, Amanda Shin, Venetia Bigley, Naomi McGovern, Pearline Teo, Peter See, Pavandip Singh Wasan, Xiao-Nong Wang, Frano Malinarich, Benoit Malleret, Anis Larbi, Pearlie Tan, Helen Zhao, Michael Poidinger, Sarah Pagan, Sharon Cookson, Rachel Dickinson, Ian Dimmick, Ruth F. Jarrett, Laurent Renia, John Tam, Colin Song, John Connolly, Jerry K.Y. Chan, Adam Gehring, Antonio Bertoletti, Matthew Collin, and Florent Ginhoux

Inventory

Figure S1

Full gating strategy to identify human DC subsets in blood, skin, liver and lung including the frequency of identified subsets as a % of CD45^{+} mononuclear cells.

Table S1

Populations purified from human and mouse for microarray profiling.

Table S2

List of gene signatures for human skin and blood monocytes and dendritic cell subsets. Available online.

Supplemental Experimental Procedures

1) Antibodies (clones) used for flow cytometry experiments.

2) Detailed microarray methods and bioinformatics analysis used in the manuscript (includes two references for bioinformatics methods).

3) Primer sequences used in RQ-PCR.

Figure S1. Gating strategy of human DC subsets

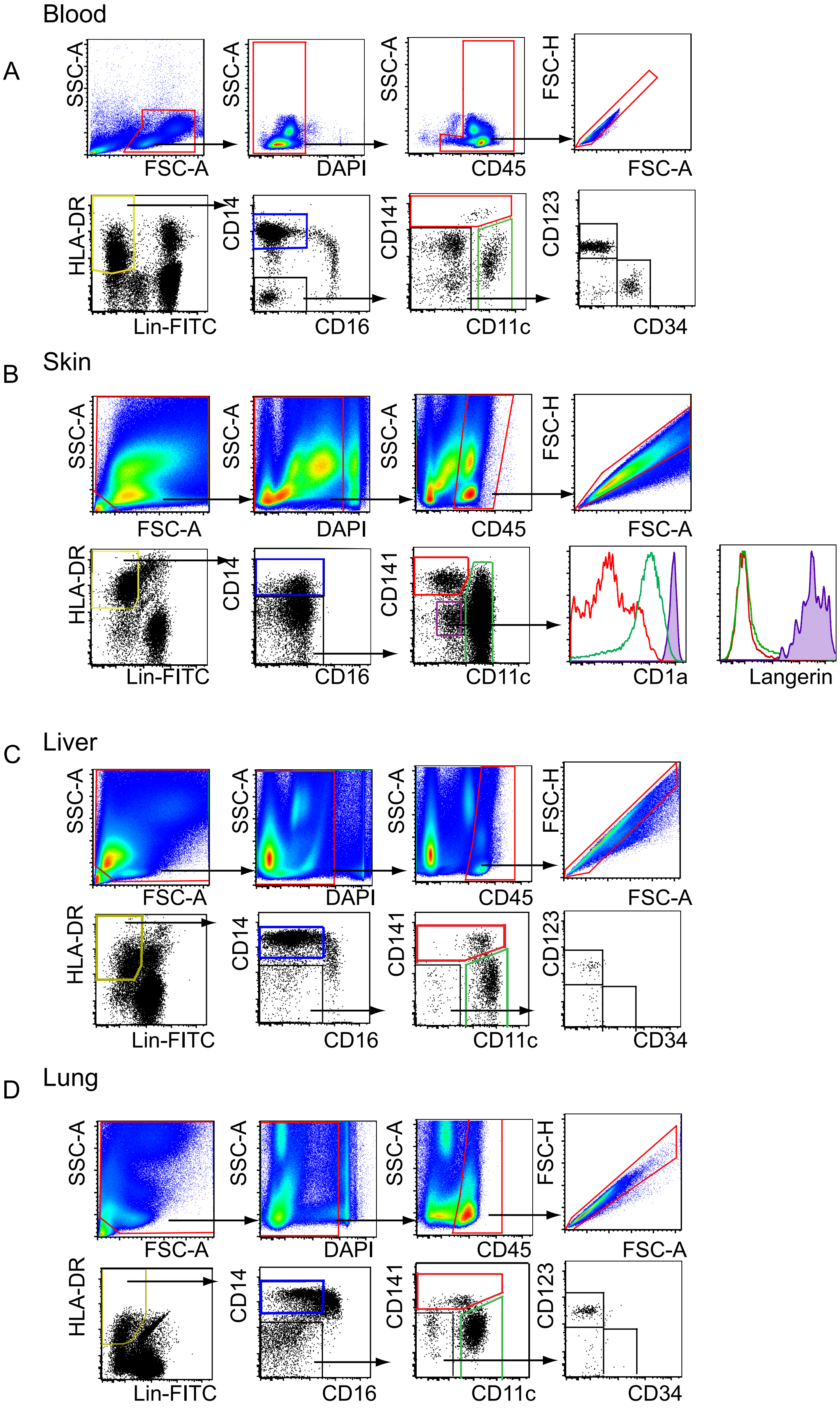

E

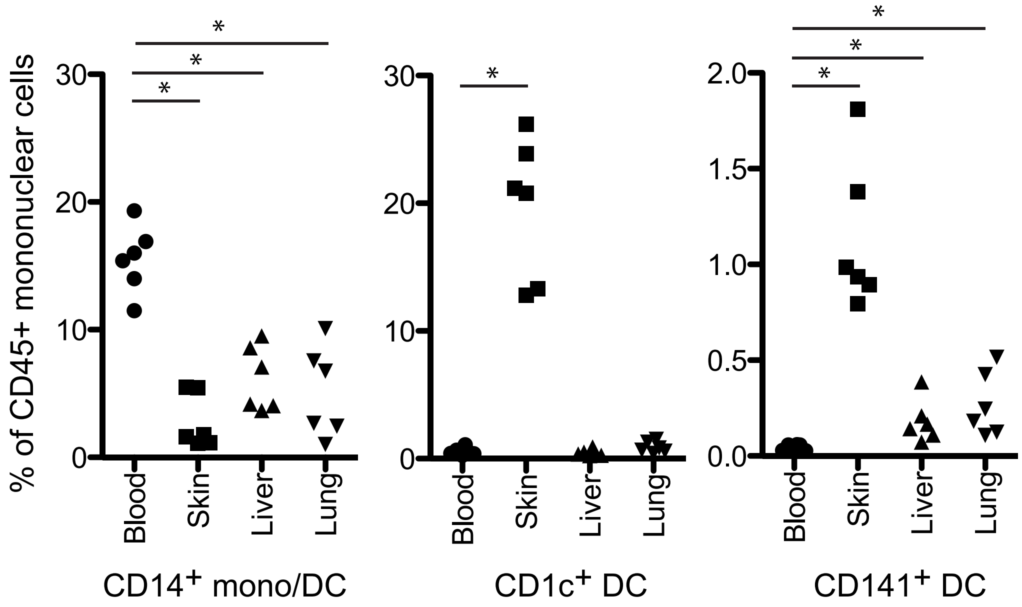

Figure S1. Related to Figure 1. Flow-cytometry gating strategy for **(A)** blood, collagenase-digested **(B)** whole skin, **(C)** liver and **(D)** mechanically-dispersed lung were analyzed using the same flow strategy to map known blood DC subsets to peripheral tissue populations. Cells were first gated by FSC-A and SSC-A and dead cells were excluded by DAPI staining. Leukocytes were identified using CD45 followed by exclusion of doublets using the FSC-H and FSC-A. Antigen Presenting Cells (APC) were identified within the HLA-DR$^+$ and Lineage$^-$ (CD3, CD19, CD20 and CD56 all in FITC) fraction. Having the Lineage cocktail in the FITC channel also allowed us to exclude autofluorescent macrophages in peripheral tissues. Three myeloid DC subsets can be identified in human tissues:1. CD14$^+$ DCs (blue gate) corresponding to blood CD14$^+$ monocytes; 2. CD1c$^+$ DCs (green) corresponding to blood CD1c$^+$ DCs; and 3. CD141^{hi} DCs corresponding to

CD141$^+$ blood DCs. CD11c$^-$CD141$^-$ cells are comprised of pDCs and CD34$^+$ cells in the blood. pDCs are absent in skin but are found in low frequency in the lung and liver. CD11c^{lo}CD141^{lo} fraction in the skin contains Langerhans cells (purple box), which are CD1a^{++} langerin^{++}. (**E**) Frequency of CD14 DC, CD1c DC and CD141 DC in blood, liver and skin as a % of CD45$^+$ mononuclear cells. Scatterplot from 6 donors are shown.

Table S1. Human and mouse DC subsets purified for microarray analysis

Human

Tissue	Subset	Gating strategy
Skin	CD14 DC	Live, CD45+ singlets HLA-DR+ AF- CD14+
	CD1c DC	Live, CD45+ singlets HLA-DR+ AF- CD14- CD11c+ CD141-
	CD1c+ CD141+	Live, CD45+ singlets HLA-DR+ AF- CD14- CD11c+ CD141+
	CD141 DC	Live, CD45+ singlets HLA-DR+ AF- CD14- CD11c lo CD141 high
Blood	CD14+ monos	Live, CD45+ singlets HLA-DR+ Lin- CD14+ CD16-
	CD16+ monos	Live, CD45+ singlets HLA-DR+ Lin- CD14lo CD16+
	pDC	Live, CD45+ singlets HLA-DR+ Lin- CD14- CD16- CD123+
	CD1c DC	Live, CD45+ singlets HLA-DR+ Lin- CD14- CD16- CD123- CD11c+ CD1c+ CD141-
	CD1141 DC	Live, CD45+ singlets HLA-DR+ Lin- CD14- CD16- CD123- CD11clo CD1c-/lo CD141+

Mouse

Tissue	Subset	Gating strategy
Spleen	CD4	Live, CD45+ singlets AF- CD11c+ IA/IE+ CD8- CD4+
	CD8	Live, CD45+ singlets AF- CD11c+ IA/IE+ CD8+
Blood	Gr1hi	Live, SSC-lo singlets CD115+ F4/80+ CD11b+ Gr1+
	Grilo	Live, SSC-lo singlets CD115+ F4/80+ CD11b+ Gr1-
Lung	CD11b total	Live, CD45+ singlets AF- CD11c+ IA/IE+ CD103- CD11b+
	CD103	Live, CD45+ singlets AF- CD11c+ IA/IE+ CD103+ CD11b-
Liver	CD11b	Live, CD45+ singlets AF- CD11c+ IA/IE+ CD103- CD11b+
	CD103	Live, CD45+ singlets AF- CD11c+ IA/IE+ CD103+ CD11b-
BM	pDC	Live, singlets lin- AF- CD11c+ IA/IE- pDCA1+ B220+ 440C+

Supplementary Materials and Methods

Antibodies for flow cytometry

Antibodies were supplied by BD unless stated otherwise and are denoted as: antigen fluorochrome (clone). CD45 V500 (HI30); CD45 PE-TexasRed (HI30; Invitrogen); CD3 FITC (SK7; UCHT1; BioLegend); CD7 FITC (4H9; eBioscience); CD19 FITC (HIB-19; BioLegend); CD20 FITC (2H7; BioLegend); CD56 FITC (MEM188; BioLegend); HLA-DR Qdot605 (Tu36; Invitrogen); HLA-DR V500 (L243); CD14 ECD (RM052; Beckman Coulter); CD16 APC-H7 (3G8; BioLegend); CD123 PerCPCy5.5 (7G3; 6H6; BioLegend); CD11c V450 (B-ly6); CD1c PECy7 (L161; BioLegend); CD141

PE or APC (AD5-14H12; Miltenyi Biotec); CD1a Alexa700 (H149; BioLegend); CD34 PE (563); CD135 PE (4G8); CLEC9A (683409; R&D); CD115 PE (61708; R&D); CX3CR1 PE (528728; R&D); CLA FITC (HECA-452; Miltenyi Biotec); CCR7 PE (3D12); CD80 PE (L307.4); CD83 PE (HB15e); CD86 (2331 (FUN-1)); PD-L1 (MIH1); langerin (DCGM4; Beckman Coulter); CD3 PerCPCy5.5 (SP34-2); CD4 APCCy7 (RPA-T4) and CD8 APC (SK1).

Microarray analysis

RNA was isolated from the sorted monocyte subsets using Qiagen RNeasy mini kit. RNA quality was confirmed using the QIAxcel analyzer (QIAGEN). A total of 300 ng of total RNA was processed using Illumina TotalPrep RNA Amplification Kit. Hybridization of human samples was performed on Illumina Human-HT12 Version 4, and mouse samples on Illumina Mouse WG6 Version 2 expression BeadChips. Data were Loess normalized without background subtraction.

All human blood and skin derived samples were pooled and differential gene expression determined with a t-test with a False Detection Rate (FDR) of 0.05. These genes were considered to be tissue specific rather than cell subtype specific and removed from the analysis. In addition, tissue specific genes were identified for blood and skin from the Tissue-specific Gene Expression and Regulation (TiGER) database (Liu et al., 2008), and also removed.

Human samples were then pooled by subtype and a 1-way ANOVA with a FDR of 0.05 was performed to identify differentially expressed genes from the pooled samples. For each gene so identified, a post-hoc t-test was

performed with an α of 0.05 contrasting each subtype in turn versus all other subtypes pooled, and statistically significant genes assigned to the respective subtype signature. The expression values for each gene were normalized by median-centering followed by division by the median absolute deviation (MAD). Genes from the signature of a given subtype that were located in the positive region of the normalized expression were considered up-regulated, and those in the negative region down-regulated. The resultant signatures for each DC subtype were then applied to each human sample using the Connectivity Map algorithm (Lamb et al., 2006), and enrichment scores were obtained. A 1000 permutation test between gene signatures was performed on each enrichment score to determine significance.

For cross species correlation, the human signatures were converted to a mouse signature by identifying gene orthologues using NCBI's HomoloGene dataset (http://www.ncbi.nlm.nih.gov/homologene/). In those cases where there was a 1-many relationship, all putative orthologues were included, except for those orthologues that were present in both the over expressed and under expressed genes of the signature, which were discarded. The converted signatures were then run against each mouse sample using the Connectivity map algorithm as before. All analyses and manipulations were carried out using the R programming language (version 2.13.2) and Pipeline Pilot (http://accelrys.com/products/pipeline-pilot/).

RQ-PCR Primer sequences

GAPDH	F1	5'-AGCCACATCGCTCAGACAC-3'
	R1	5'-GCCCAATACGACCAAATCC-3'
XCR1	F1	5'-TCAAGACGCATGTAAAGAGGTGTAG-3'
	R1	5'-GTTGCCTGAGGACTCCATCTG-3'
CLEC9A	F1	5'-TGTGTTTTCTGCATGGGATTA-3'
	R1	5'-GGTGGACACCTGCAACAAC-3'
TLR3	F1	5'-AGAGTTGTCATCGAATCAAATTAAAG-3'
	R1	5'-AATCTTCCAATTGCGTGAAAA-3'
NECL2	F1	5'-GAGTTAACATGTGAAGCCATCG-3'
	R1	5'-CGACTCTCACCCAAGTTACCA-3'

Lamb, J., Crawford, E.D., Peck, D., Modell, J.W., Blat, I.C., Wrobel, M.J., Lerner, J., Brunet, J.P., Subramanian, A., Ross, K.N., Reich, M., Hieronymus, H., Wei, G., Armstrong, S.A., Haggarty, S.J., Clemons, P.A., Wei, R., Carr, S.A., Lander, E.S., and Golub, T.R. (2006). The Connectivity Map: using gene-expression signatures to connect small molecules, genes, and disease. Science *313*, 1929–1935.

Liu, X., Yu, X., Zack, D.J., Zhu, H., and Qian, J. (2008). TiGER: a database for tissue-specific gene expression and regulation. BMC Bioinformatics *9*, 271.
