# Supplementary material for: Human Tissues Contain CD141hi Cross-Presenting Dendritic Cells with Functional Homology to Mouse CD103+ Nonlymphoid Dendritic Cells
Source: Immunity. 2012 Jul 27;37(1):60–73. doi: 10.1016/j.immuni.2012.04.012 (PMC3476529; doi:10.1016/j.immuni.2012.04.012)
Supplement: Table S2. List of Gene Signatures for Human Skin and Blood Monocytes and Dendritic Cell Subsets [file mmc2.pdf]

Table S2. Gene signatures for skin and blood DC subsets. The expression values for each gene were normalized by median-centering followed by division by the median absolute deviation (MAD). Genes from the signature of a given subtype that were located in the positive region of the normalized expression were considered up-regulated, and those in the negative region down-regulated.

CD141 lineage (upregulated genes)

ENTREZ ID	Gene Symbol	Gene Name
23677	SH3BP4	SH3-domain binding protein 4
2322	FLT3	fms-related tyrosine kinase 3
151888	BTLA	B and T lymphocyte associated
79630	C1orf54	chromosome 1 open reading frame 54
9249	DHRS3	dehydrogenase/reductase (SDR family) member 3
2317	FLNB	filamin B, beta
1803	DPP4	dipeptidyl-peptidase 4
274	BIN1	bridging integrator 1
1933	EEF1B2	eukaryotic translation elongation factor 1 beta 2
399665	FAM102A	family with sequence similarity 102, member A
257144	GCET2	germinal center expressed transcript 2
10576	CCT2	chaperonin containing TCP1, subunit 2 (beta)
79656	BEND5	BEN domain containing 5
51107	APH1A	anterior pharynx defective 1 homolog A (C. elegans)
51205	ACP6	acid phosphatase 6, lysophosphatidic
5993	RFX5	regulatory factor X, 5 (influences HLA class II expression)
3159	HMGA1	high mobility group AT-hook 1
8934	RAB7L1	RAB7, member RAS oncogene family-like 1
27346	TMEM97	transmembrane protein 97
5747	PTK2	PTK2 protein tyrosine kinase 2
85453	TSPYL5	TSPY-like 5
10263	CDK2AP2	cyclin-dependent kinase 2 associated protein 2
23521	RPL13A	ribosomal protein L13a
6193	RPS5	ribosomal protein S5
1718	DHCR24	24-dehydrocholesterol reductase
6728	SRP19	signal recognition particle 19kDa
638	BIK	BCL2-interacting killer (apoptosis-inducing)
9855	FARP2	FERM, RhoGEF and pleckstrin domain protein 2
79762	C1orf115	chromosome 1 open reading frame 115
8575	PRKRA	protein kinase, interferon-inducible double stranded RNA dependent activator
2788	GNG7	guanine nucleotide binding protein (G protein), gamma 7
2120	ETV6	ets variant 6
60481	ELOVL5	ELOVL family member 5, elongation of long chain fatty acids (FEN1/Elo2, SUR4/Elo3-like, yeast)
388574	FLJ43681	ribosomal protein L23a pseudogene
2852	GPER	G protein-coupled estrogen receptor 1
5333	PLCD1	phospholipase C, delta 1
80183	C13orf18	chromosome 13 open reading frame 18
4609	MYC	v-myc myelocytomatosis viral oncogene homolog (avian)
4724	NDUFS4	NADH dehydrogenase (ubiquinone) Fe-S protein 4, 18kDa (NADH-coenzyme Q reductase)
817	CAMK2D	calcium/calmodulin-dependent protein kinase II delta
9185	REPS2	RALBP1 associated Eps domain containing 2
730101	LOC730101	hypothetical LOC730101
6721	SREBF2	sterol regulatory element binding transcription factor 2
220930	LOC220930	hypothetical LOC220930
4171	MCM2	minichromosome maintenance complex component 2
6891	TAP2	transporter 2, ATP-binding cassette, sub-family B (MDR/TAP)
26031	OSBPL3	oxysterol binding protein-like 3
587	BCAT2	branched chain amino-acid transaminase 2, mitochondrial
641455	POTEM	POTE ankyrin domain family, member M
55227	LRRC1	leucine rich repeat containing 1
25849	PARM1	prostate androgen-regulated mucin-like protein 1
6158	RPL28	ribosomal protein L28
1616	DAXX	death-domain associated protein
10947	AP3M2	adaptor-related protein complex 3, mu 2 subunit
51571	FAM49B	family with sequence similarity 49, member B
118433	RPL23AP7	ribosomal protein L23a pseudogene 7
644511	RPL13AP6	ribosomal protein L13a pseudogene 6
1021	CDK6	cyclin-dependent kinase 6
10666	CD226	CD226 molecule
6228	RPS23	ribosomal protein S23
83988	NCALD	neurocalcin delta
25845	LOC25845	hypothetical LOC25845
51477	ISYNA1	inositol-3-phosphate synthase 1
6132	RPL8	ribosomal protein L8
84057	MND1	meiotic nuclear divisions 1 homolog (S. cerevisiae)
8099	CDK2AP1	cyclin-dependent kinase 2 associated protein 1
3066	HDAC2	histone deacetylase 2
9532	BAG2	BCL2-associated athanogene 2
4953	ODC1	ornithine decarboxylase 1
2833	CXCR3	chemokine (C-X-C motif) receptor 3
6188	RPS3	ribosomal protein S3
8458	TTF2	transcription termination factor, RNA polymerase II
5167	ENPP1	ectonucleotide pyrophosphatase/phosphodiesterase 1
100289019	LOC100289019	hypothetical LOC100289019
28978	TMEM14A	transmembrane protein 14A
6608	SMO	smoothened homolog (Drosophila)
1595	CYP51A1	cytochrome P450, family 51, subfamily A, polypeptide 1
2091	FBL	fibrillarin
57211	GPR126	G protein-coupled receptor 126
11279	KLF8	Kruppel-like factor 8
5664	PSEN2	presenilin 2 (Alzheimer disease 4)
79705	LRRK1	leucine-rich repeat kinase 1
6146	RPL22	ribosomal protein L22
3921	RPSA	ribosomal protein SA
28969	BZW2	basic leucine zipper and W2 domains 2
285148	IAH1	isoamyl acetate-hydrolyzing esterase 1 homolog (S. cerevisiae)
7982	ST7	suppression of tumorigenicity 7

51559	NT5DC3	5'-nucleotidase domain containing 3
10627	MYL12A	myosin, light chain 12A, regulatory, non-sarcomeric
692158	SNORA57	small nucleolar RNA, H/ACA box 57
8665	EIF3F	eukaryotic translation initiation factor 3, subunit F
645683	RPL13AP3	ribosomal protein L13a pseudogene 3
999	CDH1	cadherin 1, type 1, E-cadherin (epithelial)
445328	ARHGEF35	Rho guanine nucleotide exchange factor (GEF) 35
219902	TMEM136	transmembrane protein 136
55300	PI4K2B	phosphatidylinositol 4-kinase type 2 beta
5074	PAWR	PRKC, apoptosis, WT1, regulator
51320	MEX3C	mex-3 homolog C (C. elegans)
23394	ADNP	activity-dependent neuroprotector homeobox
1937	EEF1G	eukaryotic translation elongation factor 1 gamma
442421	LOC442421	hypothetical LOC442421
256355	RPS2P32	ribosomal protein S2 pseudogene 32
114883	OSBPL9	oxysterol binding protein-like 9
23555	TSPAN15	tetraspanin 15
649946	RPL23AP64	ribosomal protein L23a pseudogene 64
29803	REPIN1	replication initiator 1
5590	PRKCZ	protein kinase C, zeta
10478	SLC25A17	solute carrier family 25 (mitochondrial carrier; peroxisomal membrane protein, 34kDa), member 17
6207	RPS13	ribosomal protein S13
55526	DHTKD1	dehydrogenase E1 and transketolase domain containing 1
5539	PPY	pancreatic polypeptide
155382	VPS37D	vacuolar protein sorting 37 homolog D (S. cerevisiae)
401911	RPL29P33	ribosomal protein L29 pseudogene 33
6124	RPL4	ribosomal protein L4
729786	GOLGA8C	golgin A8 family, member C
56478	EIF4ENIF1	eukaryotic translation initiation factor 4E nuclear import factor 1
6144	RPL21	ribosomal protein L21
25873	RPL36	ribosomal protein L36
11142	PKIG	protein kinase (cAMP-dependent, catalytic) inhibitor gamma
6129	RPL7	ribosomal protein L7
6875	TAF4B	TAF4b RNA polymerase II, TATA box binding protein (TBP)-associated factor, 105kDa
10606	PAICS	phosphoribosylaminoimidazole carboxylase, phosphoribosylaminoimidazole succinocarboxamide synthetase
653162	RPSAP9	ribosomal protein SA pseudogene 9
55068	ENOX1	ecto-NOX disulfide-thiol exchanger 1
57514	ARHGAP31	Rho GTPase activating protein 31
6147	RPL23A	ribosomal protein L23a
5425	POLD2	polymerase (DNA directed), delta 2, regulatory subunit 50kDa
1975	EIF4B	eukaryotic translation initiation factor 4B
26354	GNL3	guanine nucleotide binding protein-like 3 (nucleolar)
9631	NUP155	nucleoporin 155kDa
3815	KIT	v-kit Hardy-Zuckerman 4 feline sarcoma viral oncogene homolog
23354	HAUS5	HAUS augmin-like complex, subunit 5
79856	SNX22	sorting nexin 22
7098	TLR3	toll-like receptor 3
7371	UCK2	uridine-cytidine kinase 2
6659	SOX4	SRY (sex determining region Y)-box 4
54165	DCUN1D1	DCN1, defective in cullin neddylation 1, domain containing 1 (S. cerevisiae)
57823	SLAMF7	SLAM family member 7
4649	MYO9A	myosin IXA
283345	RPL13P5	ribosomal protein L13 pseudogene 5
148	ADRA1A	adrenergic, alpha-1A-, receptor
90990	KIFC2	kinesin family member C2
57657	HCN3	hyperpolarization activated cyclic nucleotide-gated potassium channel 3
6130	RPL7A	ribosomal protein L7a
6160	RPL31	ribosomal protein L31
2529	FUT7	fucosyltransferase 7 (alpha (1,3) fucosyltransferase)
51474	LIMA1	LIM domain and actin binding 1
867	CBL	Cas-Br-M (murine) ecotropic retroviral transforming sequence
1994	ELAVL1	ELAV (embryonic lethal, abnormal vision, Drosophila)-like 1 (Hu antigen R)
9552	SPAG7	sperm associated antigen 7
79885	HDAC11	histone deacetylase 11
11224	RPL35	ribosomal protein L35
256236	NAPSB	napsin B aspartic peptidase pseudogene
440	ASNS	asparagine synthetase (glutamine-hydrolyzing)
6920	TCEA3	transcription elongation factor A (SII), 3
10866	HCP5	HLA complex P5
60	ACTB	actin, beta
9045	RPL14	ribosomal protein L14
23705	CADM1	cell adhesion molecule 1
6159	RPL29	ribosomal protein L29
29074	MRPL18	mitochondrial ribosomal protein L18
78999	LRFN4	leucine rich repeat and fibronectin type III domain containing 4
81552	VOPP1	vesicular, overexpressed in cancer, prosurvival protein 1
4065	LY75	lymphocyte antigen 75
7405	UVRAG	UV radiation resistance associated gene
7419	VDAC3	voltage-dependent anion channel 3
9727	RAB11FIP3	RAB11 family interacting protein 3 (class II)
8407	TAGLN2	transgelin 2
6189	RPS3A	ribosomal protein S3A
8502	PKP4	plakophilin 4
57475	PLEKHH1	pleckstrin homology domain containing, family H (with MyTH4 domain) member 1
6892	TAPBP	TAP binding protein (tapasin)
5250	SLC25A3	solute carrier family 25 (mitochondrial carrier; phosphate carrier), member 3
1431	CS	citrate synthase
79002	C19orf43	chromosome 19 open reading frame 43
27013	C2orf24	chromosome 2 open reading frame 24
6626	SNRPA	small nuclear ribonucleoprotein polypeptide A
201725	C4orf46	chromosome 4 open reading frame 46
56954	NIT2	nitrilase family, member 2
153769	SH3RF2	SH3 domain containing ring finger 2
64084	CLSTN2	calsyntenin 2
1501	CTNND2	catenin (cadherin-associated protein), delta 2 (neural plakophilin-related arm-repeat protein)

100505501	LOC100505501	hypothetical LOC100505501
6122	RPL3	ribosomal protein L3
3064	HTT	huntingtin
729739	LOC729739	hypothetical LOC729739
3426	CFI	complement factor I
9402	GRAP2	GRB2-related adaptor protein 2
908	CCT6A	chaperonin containing TCP1, subunit 6A (zeta 1)
54893	MTMR10	myotubularin related protein 10
57106	NAT14	N-acetyltransferase 14 (GCN5-related, putative)
116449	CLNK	cytokine-dependent hematopoietic cell linker
6205	RPS11	ribosomal protein S11
5170	PDPK1	3-phosphoinositide dependent protein kinase-1
6202	RPS8	ribosomal protein S8
51268	PIPOX	pipecolic acid oxidase
56623	INPP5E	inositol polyphosphate-5-phosphatase, 72 kDa
55911	APOBR	apolipoprotein B receptor
64110	MAGEF1	melanoma antigen family F, 1
55964	SEPT3	septin 3
5709	PSMD3	proteasome (prosome, macropain) 26S subunit, non-ATPase, 3
10428	CFDP1	craniofacial development protein 1
56924	PAK6	p21 protein (Cdc42/Rac)-activated kinase 6
55691	FRMD4A	FERM domain containing 4A
23002	DAAM1	dishevelled associated activator of morphogenesis 1
6157	RPL27A	ribosomal protein L27a
4882	NPR2	natriuretic peptide receptor B/guanylate cyclase B (atrionatriuretic peptide receptor B)
10943	MSL3	male-specific lethal 3 homolog (Drosophila)
23122	CLASP2	cytoplasmic linker associated protein 2
7307	U2AF1	U2 small nuclear RNA auxiliary factor 1
201895	C4orf34	chromosome 4 open reading frame 34
8481	OFD1	oral-facial-digital syndrome 1
51602	NOP58	NOP58 ribonucleoprotein homolog (yeast)
283420	CLEC9A	C-type lectin domain family 9, member A
51614	ERGIC3	ERGIC and golgi 3
440712	C1orf186	chromosome 1 open reading frame 186
100128252	LOC100128252	hypothetical LOC100128252
283248	RCOR2	REST corepressor 2
57128	LYRM4	LYR motif containing 4
10694	CCT8	chaperonin containing TCP1, subunit 8 (theta)
100507547	LOC100507547	hypothetical LOC100507547
145508	C14orf145	chromosome 14 open reading frame 145
8766	RAB11A	RAB11A, member RAS oncogene family
126669	SHE	Src homology 2 domain containing E
729086	LOC729086	vesicular, overexpressed in cancer, prosurvival protein 1-like
100510519	LOC100510519	HLA class II histocompatibility antigen, DR beta 4 chain-like
9760	TOX	thymocyte selection-associated high mobility group box
282618	IL29	interleukin 29 (interferon, lambda 1)
5757	PTMA	prothymosin, alpha
23234	DNAJC9	DnaJ (Hsp40) homolog, subfamily C, member 9
6194	RPS6	ribosomal protein S6
54503	ZDHHC13	zinc finger, DHHC-type containing 13
23308	ICOSLG	inducible T-cell co-stimulator ligand
8724	SNX3	sorting nexin 3
23165	NUP205	nucleoporin 205kDa
374650	GOLGA6L5	golgin A6 family-like 5 (pseudogene)
148523	C1orf51	chromosome 1 open reading frame 51
10360	NPM3	nucleophosmin/nucleoplasmin 3
22903	BTBD3	BTB (POZ) domain containing 3
55614	KIF16B	kinesin family member 16B
8611	PPAP2A	phosphatidic acid phosphatase type 2A
728658	RPL13AP5	ribosomal protein L13a pseudogene 5
100131205	RPL21P28	ribosomal protein L21 pseudogene 28
6181	RPLP2	ribosomal protein, large, P2
220963	SLC16A9	solute carrier family 16, member 9 (monocarboxylic acid transporter 9)
389114	ZNF662	zinc finger protein 662
79085	SLC25A23	solute carrier family 25 (mitochondrial carrier; phosphate carrier), member 23
6732	SRPK1	SRSF protein kinase 1
79791	FBXO31	F-box protein 31
6141	RPL18	ribosomal protein L18
339324	ZNF260	zinc finger protein 260
6137	RPL13	ribosomal protein L13
23107	MRPS27	mitochondrial ribosomal protein S27
6152	RPL24	ribosomal protein L24
2281	FKBP1B	FK506 binding protein 1B, 12.6 kDa
84707	BEX2	brain expressed X-linked 2
6001	RGS10	regulator of G-protein signaling 10
51692	CPSF3	cleavage and polyadenylation specific factor 3, 73kDa
54897	CASZ1	castor zinc finger 1
56265	CPXM1	carboxypeptidase X (M14 family), member 1
84433	CARD11	caspase recruitment domain family, member 11
5168	ENPP2	ectonucleotide pyrophosphatase/phosphodiesterase 2
3925	STMN1	stathmin 1
4642	MYO1D	myosin ID
2107	ETF1	eukaryotic translation termination factor 1
55619	DOCK10	dedicator of cytokinesis 10
115290	FBXO17	F-box protein 17
689	BTF3	basic transcription factor 3
6210	RPS15A	ribosomal protein S15a
3111	HLA-DOA	major histocompatibility complex, class II, DO alpha
1632	DCI	dodecenoyl-CoA isomerase
4173	MCM4	minichromosome maintenance complex component 4
5335	PLCG1	phospholipase C, gamma 1
5364	PLXNB1	plexin B1
29121	CLEC2D	C-type lectin domain family 2, member D
57591	MKL1	megakaryoblastic leukemia (translocation) 1
9126	SMC3	structural maintenance of chromosomes 3

5478	PPIA	peptidylprolyl isomerase A (cyclophilin A)
125144	NCRNA00188	non-protein coding RNA 188
728643	LOC728643	heterogeneous nuclear ribonucleoprotein A1 pseudogene
81620	CDT1	chromatin licensing and DNA replication factor 1
388564	LOC388564	hypothetical protein LOC388564
10093	ARPC4	actin related protein 2/3 complex, subunit 4, 20kDa
57507	ZNF608	zinc finger protein 608
81544	GDPD5	glycerophosphodiester phosphodiesterase domain containing 5
6175	RPLP0	ribosomal protein, large, P0
84067	FAM160A2	family with sequence similarity 160, member A2
6230	RPS25	ribosomal protein S25
286077	FAM83H	family with sequence similarity 83, member H
607	BCL9	B-cell CLL/lymphoma 9
6209	RPS15	ribosomal protein S15
23623	RUSC1	RUN and SH3 domain containing 1
2531	KDSR	3-ketodihydrosphingosine reductase
6233	RPS27A	ribosomal protein S27a
158405	KIAA1958	KIAA1958
8971	H1FX	H1 histone family, member X
991	CDC20	cell division cycle 20 homolog (S. cerevisiae)
6138	RPL15	ribosomal protein L15
55218	EXD2	exonuclease 3'-5' domain containing 2
286102	TMED10P1	transmembrane emp24-like trafficking protein 10 (yeast) pseudogene 1
6235	RPS29	ribosomal protein S29
6047	RNF4	ring finger protein 4
6804	STX1A	syntaxin 1A (brain)
116159	CYYR1	cysteine/tyrosine-rich 1
3190	HNRNPK	heterogeneous nuclear ribonucleoprotein K
11104	KATNA1	katanin p60 (ATPase containing) subunit A 1
6176	RPLP1	ribosomal protein, large, P1
80063	ATF7IP2	activating transcription factor 7 interacting protein 2
8669	EIF3J	eukaryotic translation initiation factor 3, subunit J
23483	TGDS	TDP-glucose 4,6-dehydratase
8975	USP13	ubiquitin specific peptidase 13 (isopeptidase T-3)
2829	XCR1	chemokine (C motif) receptor 1
57060	PCBP4	poly(rC) binding protein 4
79657	RPAP3	RNA polymerase II associated protein 3
51203	NUSAP1	nucleolar and spindle associated protein 1
8664	EIF3D	eukaryotic translation initiation factor 3, subunit D
6136	RPL12	ribosomal protein L12
6171	RPL41	ribosomal protein L41
401884	MGC57346	hypothetical LOC401884
10100	TSPAN2	tetraspanin 2
57037	ANKMY2	ankyrin repeat and MYND domain containing 2
9991	ROD1	ROD1 regulator of differentiation 1 (S. pombe)
5901	RAN	RAN, member RAS oncogene family
2948	GSTM4	glutathione S-transferase mu 4
134	ADORA1	adenosine A1 receptor
9871	SEC24D	SEC24 family, member D (S. cerevisiae)
81793	TLR10	toll-like receptor 10
109	ADCY3	adenylate cyclase 3
55509	BATF3	basic leucine zipper transcription factor, ATF-like 3
339005	WHAMML1	WAS protein homolog associated with actin, golgi membranes and microtubules-like 1 (pseudogene)
126668	TDRD10	tudor domain containing 10
10810	WASF3	WAS protein family, member 3
57705	WDFY4	WDFY family member 4
3615	IMPDH2	IMP (inosine 5'-monophosphate) dehydrogenase 2
81539	SLC38A1	solute carrier family 38, member 1
57579	FAM135A	family with sequence similarity 135, member A
56969	RPL23AP32	ribosomal protein L23a pseudogene 32
7570	ZNF22	zinc finger protein 22 (KOX 15)
10505	SEMA4F	sema domain, immunoglobulin domain(Ig), transmembrane domain(TM) and short cytoplasmic domain, (semaphorin) 4F
170960	ZNF721	zinc finger protein 721
3902	LAG3	lymphocyte-activation gene 3
196996	GRAMD2	GRAM domain containing 2
50628	GEMIN4	gem (nuclear organelle) associated protein 4
55697	VAC14	Vac14 homolog (S. cerevisiae)
283683	LOC283683	hypothetical LOC283683
865	CBFB	core-binding factor, beta subunit
6168	RPL37A	ribosomal protein L37a
2950	GSTP1	glutathione S-transferase pi 1
9889	ZBED4	zinc finger, BED-type containing 4
80728	ARHGAP39	Rho GTPase activating protein 39
23231	SEL1L3	sel-1 suppressor of lin-12-like 3 (C. elegans)
8318	CDC45	cell division cycle 45 homolog (S. cerevisiae)
9349	RPL23	ribosomal protein L23
646567	LOC646567	oligosaccharyltransferase complex subunit OSTC-like
51199	NIN	ninein (GSK3B interacting protein)
9319	TRIP13	thyroid hormone receptor interactor 13
253769	WDR27	WD repeat domain 27
2944	GSTM1	glutathione S-transferase mu 1
6187	RPS2	ribosomal protein S2
3092	HIP1	huntingtin interacting protein 1
152559	PAQR3	progestin and adipoQ receptor family member III
5863	RGL2	ral guanine nucleotide dissociation stimulator-like 2
10236	HNRNPR	heterogeneous nuclear ribonucleoprotein R
6134	RPL10	ribosomal protein L10
2926	GRSF1	G-rich RNA sequence binding factor 1
79697	C14orf169	chromosome 14 open reading frame 169
84451	KIAA1804	mixed lineage kinase 4
1465	CSRP1	cysteine and glycine-rich protein 1
26003	GORASP2	golgi reassembly stacking protein 2, 55kDa
57600	FNIP2	folliculin interacting protein 2
162979	ZNF296	zinc finger protein 296
23264	ZC3H7B	zinc finger CCCH-type containing 7B

387066	SNHG5	small nucleolar RNA host gene 5 (non-protein coding)
6201	RPS7	ribosomal protein S7
404785	POTEG	POTE ankyrin domain family, member G
100188949	LOC100188949	hypothetical LOC100188949
116028	C16orf75	chromosome 16 open reading frame 75
11340	EXOSC8	exosome component 8
123228	SENP8	SUMO/sentrin specific peptidase family member 8
4839	NOP2	NOP2 nucleolar protein homolog (yeast)
80142	PTGES2	prostaglandin E synthase 2
80145	THOC7	THO complex 7 homolog (Drosophila)
2781	GNAZ	guanine nucleotide binding protein (G protein), alpha z polypeptide
262	AMD1	adenosylmethionine decarboxylase 1
166614	DCLK2	doublecortin-like kinase 2
1000	CDH2	cadherin 2, type 1, N-cadherin (neuronal)
6155	RPL27	ribosomal protein L27
2194	FASN	fatty acid synthase
283102	FLJ46111	keratin 8 pseudogene
7984	ARHGEF5	Rho guanine nucleotide exchange factor (GEF) 5
5037	PEBP1	phosphatidylethanolamine binding protein 1
23151	GRAMD4	GRAM domain containing 4
100130613	CXorf64	chromosome X open reading frame 64
6421	SFPQ	splicing factor proline/glutamine-rich
476	ATP1A1	ATPase, Na+/K+ transporting, alpha 1 polypeptide
51678	MPP6	membrane protein, palmitoylated 6 (MAGUK p55 subfamily member 6)
10106	CTDSP2	CTD (carboxy-terminal domain, RNA polymerase II, polypeptide A) small phosphatase 2
8869	ST3GAL5	ST3 beta-galactoside alpha-2,3-sialyltransferase 5
1503	CTPS	CTP synthase
6242	RTKN	rhotekin
3146	HMGB1	high-mobility group box 1
1350	COX7C	cytochrome c oxidase subunit VIIc
9978	RBX1	ring-box 1, E3 ubiquitin protein ligase
27316	RBMX	RNA binding motif protein, X-linked
11332	ACOT7	acyl-CoA thioesterase 7
51639	SF3B14	splicing factor 3B, 14 kDa subunit
10009	ZBTB33	zinc finger and BTB domain containing 33
3006	HIST1H1C	histone cluster 1, H1c
344148	NCKAP5	NCK-associated protein 5
4799	NFX1	nuclear transcription factor, X-box binding 1
343477	HSP90B3P	heat shock protein 90kDa beta (Grp94), member 3 (pseudogene)
6389	SDHA	succinate dehydrogenase complex, subunit A, flavoprotein (Fp)
5176	SERPINF1	serpin peptidase inhibitor, clade F (alpha-2 antiplasmin, pigment epithelium derived factor), member 1
3418	IDH2	isocitrate dehydrogenase 2 (NADP+), mitochondrial
84660	CCDC62	coiled-coil domain containing 62
1139	CHRNA7	cholinergic receptor, nicotinic, alpha 7
6161	RPL32	ribosomal protein L32
79877	DCAKD	dephospho-CoA kinase domain containing
1265	CNN2	calponin 2
51339	DACT1	dapper, antagonist of beta-catenin, homolog 1 (Xenopus laevis)
121268	RHEBL1	Ras homolog enriched in brain like 1
55915	LANCL2	LanC lantibiotic synthetase component C-like 2 (bacterial)
85015	USP45	ubiquitin specific peptidase 45
2729	GCLC	glutamate-cysteine ligase, catalytic subunit
6206	RPS12	ribosomal protein S12
9333	TGM5	transglutaminase 5
55959	SULF2	sulfatase 2
440563	LOC440563	heterogeneous nuclear ribonucleoprotein C-like
6229	RPS24	ribosomal protein S24
100289079	LOC100289079	60S ribosomal protein L36-like
55276	PGM2	phosphoglucomutase 2
65250	C5orf42	chromosome 5 open reading frame 42
4673	NAP1L1	nucleosome assembly protein 1-like 1
57026	PDXP	pyridoxal (pyridoxine, vitamin B6) phosphatase
6196	RPS6KA2	ribosomal protein S6 kinase, 90kDa, polypeptide 2
84617	TUBB6	tubulin, beta 6
8850	KAT2B	K(lysine) acetyltransferase 2B
5134	PDCD2	programmed cell death 2
401647	GOLGA7B	golgin A7 family, member B
23390	ZDHHC17	zinc finger, DHHC-type containing 17
148789	B3GALNT2	beta-1,3-N-acetylgalactosaminyltransferase 2
440574	C1orf151	chromosome 1 open reading frame 151
338440	ANO9	anoctamin 9
84275	SLC25A33	solute carrier family 25, member 33
343413	FCRL6	Fc receptor-like 6
2286	FKBP2	FK506 binding protein 2, 13kDa
56922	MCCC1	methylcrotonoyl-CoA carboxylase 1 (alpha)
4723	NDUFV1	NADH dehydrogenase (ubiquinone) flavoprotein 1, 51kDa
3490	IGFBP7	insulin-like growth factor binding protein 7
8634	RTCD1	RNA terminal phosphate cyclase domain 1
6814	STXBP3	syntaxin binding protein 3
79174	CRELD2	cysteine-rich with EGF-like domains 2
10845	CLPX	ClpX caseinolytic peptidase X homolog (E. coli)
80758	PRR7	proline rich 7 (synaptic)
10096	ACTR3	ARP3 actin-related protein 3 homolog (yeast)
692148	SCARNA10	small Cajal body-specific RNA 10
8349	HIST2H2BE	histone cluster 2, H2be
6143	RPL19	ribosomal protein L19
25904	CNOT10	CCR4-NOT transcription complex, subunit 10
7411	VBP1	von Hippel-Lindau binding protein 1
64073	C19orf33	chromosome 19 open reading frame 33
6191	RPS4X	ribosomal protein S4, X-linked
23762	OSBP2	oxysterol binding protein 2
55922	NKRF	NFKB repressing factor
3275	PRMT2	protein arginine methyltransferase 2
9651	PLCH2	phospholipase C, eta 2
9394	HS6ST1	heparan sulfate 6-O-sulfotransferase 1

376693	RPS10P7	ribosomal protein S10 pseudogene 7
728743	LOC728743	similar to GLI-Kruppel family member HKR1
6234	RPS28	ribosomal protein S28
4736	RPL10A	ribosomal protein L10a
9377	COX5A	cytochrome c oxidase subunit Va
23157	SEPT6	septin 6
9025	RNF8	ring finger protein 8
51011	FAHD2A	fumarylacetoacetate hydrolase domain containing 2A
163	AP2B1	adaptor-related protein complex 2, beta 1 subunit
115286	SLC25A26	solute carrier family 25, member 26
390539	OR4N3P	olfactory receptor, family 4, subfamily N, member 3 pseudogene
5445	PON2	paraoxonase 2
203427	SLC25A43	solute carrier family 25, member 43
404770	FAM75B	family with sequence similarity 75, member B
6125	RPL5	ribosomal protein L5
352961	HCG26	HLA complex group 26 (non-protein coding)
7386	UQCRFS1	ubiquinol-cytochrome c reductase, Rieske iron-sulfur polypeptide 1
6640	SNTA1	syntrophin, alpha 1 (dystrophin-associated protein A1, 59kDa, acidic component)
388962	BOLA3	bolA homolog 3 (E. coli)
26060	APPL1	adaptor protein, phosphotyrosine interaction, PH domain and leucine zipper containing 1
293	SLC25A6	solute carrier family 25 (mitochondrial carrier; adenine nucleotide translocator), member 6
8883	NAE1	NEDD8 activating enzyme E1 subunit 1
55082	ARGLU1	arginine and glutamate rich 1
6154	RPL26	ribosomal protein L26
26996	GPR160	G protein-coupled receptor 160
151313	FAHD2B	fumarylacetoacetate hydrolase domain containing 2B
788	SLC25A20	solute carrier family 25 (carnitine/acylcarnitine translocase), member 20
10213	PSMD14	proteasome (prosome, macropain) 26S subunit, non-ATPase, 14
55552	ZNF823	zinc finger protein 823
57599	WDR48	WD repeat domain 48
81853	TMEM14B	transmembrane protein 14B
6647	SOD1	superoxide dismutase 1, soluble
80212	CCDC92	coiled-coil domain containing 92
5341	PLEK	pleckstrin
79864	C11orf63	chromosome 11 open reading frame 63
57711	ZNF529	zinc finger protein 529
280655	C14orf19	immunoglobulin (CD79A) binding protein 1 pseudogene
3703	STT3A	STT3, subunit of the oligosaccharyltransferase complex, homolog A (S. cerevisiae)
51503	CWC15	CWC15 spliceosome-associated protein homolog (S. cerevisiae)
3108	HLA-DMA	major histocompatibility complex, class II, DM alpha
6232	RPS27	ribosomal protein S27
9117	SEC22C	SEC22 vesicle trafficking protein homolog C (S. cerevisiae)
23244	PDS5A	PDS5, regulator of cohesion maintenance, homolog A (S. cerevisiae)
6133	RPL9	ribosomal protein L9
60436	TGIF2	TGFB-induced factor homeobox 2
9744	ACAP1	ArfGAP with coiled-coil, ankyrin repeat and PH domains 1
80305	TRABD	TraB domain containing
2339	FNTA	farnesyltransferase, CAAX box, alpha
81608	FIP1L1	FIP1 like 1 (S. cerevisiae)
51187	RSL24D1	ribosomal L24 domain containing 1
5805	PTS	6-pyruvoyltetrahydropterin synthase
58505	OSTC	oligosaccharyltransferase complex subunit
79443	FYCO1	FYVE and coiled-coil domain containing 1
381	ARF5	ADP-ribosylation factor 5
286053	NSMCE2	non-SMC element 2, MMS21 homolog (S. cerevisiae)

CD141 lineage (downregulated genes)

ENTREZ ID	Gene Symbol	Gene Name
121551	BTBD11	BTB (POZ) domain containing 11
54453	RIN2	Ras and Rab interactor 2
114769	CARD16	caspase recruitment domain family, member 16
9046	DOK2	docking protein 2, 56kDa
837	CASP4	caspase 4, apoptosis-related cysteine peptidase
83719	YPEL3	yippee-like 3 (Drosophila)
29992	PILRA	paired immunoglobin-like type 2 receptor alpha
79168	LILRA6	leukocyte immunoglobulin-like receptor, subfamily A (with TM domain), member 6
2799	GNS	glucosamine (N-acetyl)-6-sulfatase
80727	TTYH3	tweety homolog 3 (Drosophila)
1436	CSF1R	colony stimulating factor 1 receptor
11213	IRAK3	interleukin-1 receptor-associated kinase 3
219855	SLC37A2	solute carrier family 37 (glycerol-3-phosphate transporter), member 2
1462	VCAN	versican
834	CASP1	caspase 1, apoptosis-related cysteine peptidase (interleukin 1, beta, convertase)
51296	SLC15A3	solute carrier family 15, member 3
341405	ANKRD33	ankyrin repeat domain 33
968	CD68	CD68 molecule
5265	SERPINA1	serpin peptidase inhibitor, clade A (alpha-1 antiproteinase, antitrypsin), member 1
64411	ARAP3	ArfGAP with RhoGAP domain, ankyrin repeat and PH domain 3
7739	ZNF185	zinc finger protein 185 (LIM domain)
11033	ADAP1	ArfGAP with dual PH domains 1
27287	VENTX	VENT homeobox homolog (Xenopus laevis)
11025	LILRB3	leukocyte immunoglobulin-like receptor, subfamily B (with TM and ITIM domains), member 3
79901	CYBRD1	cytochrome b reductase 1
5724	PTAFR	platelet-activating factor receptor
10581	IFITM2	interferon induced transmembrane protein 2 (1-8D)
100049587	SIGLEC14	sialic acid binding Ig-like lectin 14
10312	TCIRG1	T-cell, immune regulator 1, ATPase, H+ transporting, lysosomal V0 subunit A3
2633	GBP1	guanylate binding protein 1, interferon-inducible
3176	HNMT	histamine N-methyltransferase
9404	LPXN	leupaxin
79720	VPS37B	vacuolar protein sorting 37 homolog B (S. cerevisiae)
27239	GPR162	G protein-coupled receptor 162
55365	TMEM176A	transmembrane protein 176A
84293	C10orf58	chromosome 10 open reading frame 58
1508	CTSB	cathepsin B

137835	TMEM71	transmembrane protein 71
162989	DEDD2	death effector domain containing 2
100129550	LOC100129550	hypothetical LOC100129550
6453	ITSN1	intersectin 1 (SH3 domain protein)
404636	FAM45A	family with sequence similarity 45, member A
116985	ARAP1	ArfGAP with RhoGAP domain, ankyrin repeat and PH domain 1
9839	ZEB2	zinc finger E-box binding homeobox 2
4043	LRPAP1	low density lipoprotein receptor-related protein associated protein 1
8569	MKNK1	MAP kinase interacting serine/threonine kinase 1
3964	LGALS8	lectin, galactoside-binding, soluble, 8
55763	EXOC1	exocyst complex component 1
3916	LAMP1	lysosomal-associated membrane protein 1
6252	RTN1	reticulon 1
929	CD14	CD14 molecule
10410	IFITM3	interferon induced transmembrane protein 3 (1-8U)
91351	DDX60L	DEAD (Asp-Glu-Ala-Asp) box polypeptide 60-like
7099	TLR4	toll-like receptor 4
54540	FAM193B	family with sequence similarity 193, member B
5476	CTSA	cathepsin A
10439	OLFM1	olfactomedin 1
4126	MANBA	mannosidase, beta A, lysosomal
8564	KMO	kynurenine 3-monooxygenase (kynurenine 3-hydroxylase)
10924	SMPDL3A	sphingomyelin phosphodiesterase, acid-like 3A
5578	PRKCA	protein kinase C, alpha
84034	EMILIN2	elastin microfibril interfacer 2
93589	CACNA2D4	calcium channel, voltage-dependent, alpha 2/delta subunit 4
59	ACTA2	actin, alpha 2, smooth muscle, aorta
57477	SHROOM4	shroom family member 4
3311	HSPA7	heat shock 70kDa protein 7 (HSP70B)
113878	DTX2	deltex homolog 2 (Drosophila)
1050	CEBPA	CCAAT/enhancer binding protein (C/EBP), alpha
4354	MPP1	membrane protein, palmitoylated 1, 55kDa
2550	GABBR1	gamma-aminobutyric acid (GABA) B receptor, 1
115948	CCDC151	coiled-coil domain containing 151
10288	LILRB2	leukocyte immunoglobulin-like receptor, subfamily B (with TM and ITIM domains), member 2
100506144	LOC100506144	hypothetical protein LOC100506144
80267	EDEM3	ER degradation enhancer, mannosidase alpha-like 3
113675	SDSL	serine dehydratase-like
2537	IFI6	interferon, alpha-inducible protein 6
3305	HSPA1L	heat shock 70kDa protein 1-like
112770	C1orf85	chromosome 1 open reading frame 85
7132	TNFRSF1A	tumor necrosis factor receptor superfamily, member 1A
64207	IRF2BPL	interferon regulatory factor 2 binding protein-like
51257	MARCH2	membrane-associated ring finger (C3HC4) 2
2634	GBP2	guanylate binding protein 2, interferon-inducible
1138	CHRNA5	cholinergic receptor, nicotinic, alpha 5
10170	DHRS9	dehydrogenase/reductase (SDR family) member 9
6237	RRAS	related RAS viral (r-ras) oncogene homolog
5210	PFKFB4	6-phosphofructo-2-kinase/fructose-2,6-biphosphatase 4
115825	WDFY2	WD repeat and FYVE domain containing 2
55654	TMEM127	transmembrane protein 127
57192	MCOLN1	mucolipin 1
54458	PRR13	proline rich 13
27348	TOR1B	torsin family 1, member B (torsin B)
80896	NPL	N-acetylneuraminate pyruvate lyase (dihydrodipicolinate synthase)
10673	TNFSF13B	tumor necrosis factor (ligand) superfamily, member 13b
200030	NBPF11	neuroblastoma breakpoint family, member 11
79042	TSEN34	tRNA splicing endonuclease 34 homolog (S. cerevisiae)
54861	SNRK	SNF related kinase
3784	KCNQ1	potassium voltage-gated channel, KQT-like subfamily, member 1
79594	MUL1	mitochondrial E3 ubiquitin protein ligase 1
2131	EXT1	exostosin 1
126014	OSCAR	osteoclast associated, immunoglobulin-like receptor
9446	GSTO1	glutathione S-transferase omega 1
55788	LMBRD1	LMBR1 domain containing 1
65018	PINK1	PTEN induced putative kinase 1
9354	UBE4A	ubiquitination factor E4A (UFD2 homolog, yeast)
79650	C16orf57	chromosome 16 open reading frame 57
54965	PIGX	phosphatidylinositol glycan anchor biosynthesis, class X
10154	PLXNC1	plexin C1
57794	SUGP1	SURP and G patch domain containing 1
353514	LILRA5	leukocyte immunoglobulin-like receptor, subfamily A (with TM domain), member 5
256586	LYSMD2	LysM, putative peptidoglycan-binding, domain containing 2
55022	PID1	phosphotyrosine interaction domain containing 1
84689	MS4A14	membrane-spanning 4-domains, subfamily A, member 14
9466	IL27RA	interleukin 27 receptor, alpha
25979	DHRS7B	dehydrogenase/reductase (SDR family) member 7B
58526	MID1IP1	MID1 interacting protein 1 (gastrulation specific G12 homolog (zebrafish))
79065	ATG9A	ATG9 autophagy related 9 homolog A (S. cerevisiae)
9550	ATP6V1G1	ATPase, H+ transporting, lysosomal 13kDa, V1 subunit G1
8675	STX16	syntaxin 16
100132967	NCRNA00204	non-protein coding RNA 204
55357	TBC1D2	TBC1 domain family, member 2
146923	RUNDC1	RUN domain containing 1
8650	NUMB	numb homolog (Drosophila)
84173	ELMOD3	ELMO/CED-12 domain containing 3
26262	TSPAN17	tetraspanin 17
84193	SETD3	SET domain containing 3
718	C3	complement component 3
8451	CUL4A	cullin 4A
55760	DHX32	DEAH (Asp-Glu-Ala-His) box polypeptide 32
148867	SLC30A7	solute carrier family 30 (zinc transporter), member 7
10908	PNPLA6	patatin-like phospholipase domain containing 6
11018	TMED1	transmembrane emp24 protein transport domain containing 1
8819	SAP30	Sin3A-associated protein, 30kDa

83464	APH1B	anterior pharynx defective 1 homolog B (C. elegans)
79132	DHX58	DEXH (Asp-Glu-X-His) box polypeptide 58
1051	CEBPB	CCAAT/enhancer binding protein (C/EBP), beta
6548	SLC9A1	solute carrier family 9 (sodium/hydrogen exchanger), member 1
29097	CNIH4	cornichon homolog 4 (Drosophila)
196383	RILPL2	Rab interacting lysosomal protein-like 2
57590	WDFY1	WD repeat and FYVE domain containing 1
3588	IL10RB	interleukin 10 receptor, beta
144402	CPNE8	copine VIII
57674	RNF213	ring finger protein 213
64429	ZDHHC6	zinc finger, DHHC-type containing 6
51311	TLR8	toll-like receptor 8
5286	PIK3C2A	phosphoinositide-3-kinase, class 2, alpha polypeptide
6195	RPS6KA1	ribosomal protein S6 kinase, 90kDa, polypeptide 1
9826	ARHGEF11	Rho guanine nucleotide exchange factor (GEF) 11
203054	ADCK5	aarF domain containing kinase 5
11182	SLC2A6	solute carrier family 2 (facilitated glucose transporter), member 6
126003	TRAPPC5	trafficking protein particle complex 5
5394	EXOSC10	exosome component 10
23294	ANKS1A	ankyrin repeat and sterile alpha motif domain containing 1A
93594	WDR67	WD repeat domain 67
55344	PLCXD1	phosphatidylinositol-specific phospholipase C, X domain containing 1
55625	ZDHHC7	zinc finger, DHHC-type containing 7
10870	HCST	hematopoietic cell signal transducer
103	ADAR	adenosine deaminase, RNA-specific
666	BOK	BCL2-related ovarian killer
5326	PLAGL2	pleiomorphic adenoma gene-like 2
80325	ABTB1	ankyrin repeat and BTB (POZ) domain containing 1
96459	FNIP1	folliculin interacting protein 1
90639	COX19	COX19 cytochrome c oxidase assembly homolog (S. cerevisiae)
58475	MS4A7	membrane-spanning 4-domains, subfamily A, member 7
145389	SLC38A6	solute carrier family 38, member 6
100287171	WASH1	WAS protein family homolog 1
6655	SOS2	son of sevenless homolog 2 (Drosophila)
27128	CYTH4	cytohesin 4
6901	TAZ	tafazzin
9382	COG1	component of oligomeric golgi complex 1
55823	VPS11	vacuolar protein sorting 11 homolog (S. cerevisiae)
3454	IFNAR1	interferon (alpha, beta and omega) receptor 1
55505	NOP10	NOP10 ribonucleoprotein homolog (yeast)
1659	DHX8	DEAH (Asp-Glu-Ala-His) box polypeptide 8
84515	MCM8	minichromosome maintenance complex component 8
9960	USP3	ubiquitin specific peptidase 3
6556	SLC11A1	solute carrier family 11 (proton-coupled divalent metal ion transporters), member 1
29116	MYLIP	myosin regulatory light chain interacting protein
3455	IFNAR2	interferon (alpha, beta and omega) receptor 2
57583	TMEM181	transmembrane protein 181
442578	STAG3L3	stromal antigen 3-like 3
54788	DNAJB12	DnaJ (Hsp40) homolog, subfamily B, member 12
53	ACP2	acid phosphatase 2, lysosomal
80736	SLC44A4	solute carrier family 44, member 4
9474	ATG5	ATG5 autophagy related 5 homolog (S. cerevisiae)
10092	ARPC5	actin related protein 2/3 complex, subunit 5, 16kDa
374882	TMEM205	transmembrane protein 205
6621	SNAPC4	small nuclear RNA activating complex, polypeptide 4, 190kDa
201294	UNC13D	unc-13 homolog D (C. elegans)
5305	PIP4K2A	phosphatidylinositol-5-phosphate 4-kinase, type II, alpha
138151	NACC2	NACC family member 2, BEN and BTB (POZ) domain containing
54856	GON4L	gon-4-like (C. elegans)
8605	PLA2G4C	phospholipase A2, group IVC (cytosolic, calcium-independent)
9673	SLC25A44	solute carrier family 25, member 44
54834	GDAP2	ganglioside induced differentiation associated protein 2
51023	MRPS18C	mitochondrial ribosomal protein S18C
943	TNFRSF8	tumor necrosis factor receptor superfamily, member 8
9935	MAFB	v-maf musculoaponeurotic fibrosarcoma oncogene homolog B (avian)
60684	C4orf41	chromosome 4 open reading frame 41
2885	GRB2	growth factor receptor-bound protein 2
5606	MAP2K3	mitogen-activated protein kinase kinase 3
5297	PI4KA	phosphatidylinositol 4-kinase, catalytic, alpha
54507	ADAMTSL4	ADAMTS-like 4
2869	GRK5	G protein-coupled receptor kinase 5
8705	B3GALT4	UDP-Gal:betaGlcNAc beta 1,3-galactosyltransferase, polypeptide 4
4122	MAN2A2	mannosidase, alpha, class 2A, member 2
3241	HPCAL1	hippocalcin-like 1
8402	SLC25A11	solute carrier family 25 (mitochondrial carrier; oxoglutarate carrier), member 11
440836	ODF3B	outer dense fiber of sperm tails 3B
51092	SIDT2	SID1 transmembrane family, member 2
326	AIRE	autoimmune regulator
1318	SLC31A2	solute carrier family 31 (copper transporters), member 2
51168	MYO15A	myosin XVA
56034	PDGFC	platelet derived growth factor C
201627	FAM116A	family with sequence similarity 116, member A
85450	ITPRIP	inositol 1,4,5-triphosphate receptor interacting protein
6752	SSTR2	somatostatin receptor 2
23247	KIAA0556	KIAA0556
83666	PARP9	poly (ADP-ribose) polymerase family, member 9
79602	ADIPOR2	adiponectin receptor 2
81844	TRIM56	tripartite motif containing 56
5184	PEPD	peptidase D
8519	IFITM1	interferon induced transmembrane protein 1 (9-27)
55858	TMEM165	transmembrane protein 165
55051	C14orf102	chromosome 14 open reading frame 102
80256	KIAA1539	KIAA1539
10899	JTB	jumping translocation breakpoint
374395	TMEM179B	transmembrane protein 179B

6813	STXBP2	syntaxin binding protein 2
3783	KCNN4	potassium intermediate/small conductance calcium-activated channel, subfamily N, member 4
8915	BCL10	B-cell CLL/lymphoma 10
3732	CD82	CD82 molecule
5351	PLOD1	procollagen-lysine 1, 2-oxoglutarate 5-dioxygenase 1
64114	TMBIM1	transmembrane BAX inhibitor motif containing 1
527	ATP6V0C	ATPase, H+ transporting, lysosomal 16kDa, V0 subunit c
2733	GLE1	GLE1 RNA export mediator homolog (yeast)
30827	CXXC1	CXXC finger protein 1
8893	EIF2B5	eukaryotic translation initiation factor 2B, subunit 5 epsilon, 82kDa
3098	HK1	hexokinase 1
114785	MBD6	methyl-CpG binding domain protein 6
64759	TNS3	tensin 3
221662	RBM24	RNA binding motif protein 24
64077	LHPP	phospholysine phosphohistidine inorganic pyrophosphate phosphatase
55601	DDX60	DEAD (Asp-Glu-Ala-Asp) box polypeptide 60
10062	NR1H3	nuclear receptor subfamily 1, group H, member 3
6390	SDHB	succinate dehydrogenase complex, subunit B, iron sulfur (Ip)
152815	THAP6	THAP domain containing 6
537	ATP6AP1	ATPase, H+ transporting, lysosomal accessory protein 1
147798	TMC4	transmembrane channel-like 4
29919	C18orf8	chromosome 18 open reading frame 8
51765	MST4	serine/threonine protein kinase MST4
92595	ZNF764	zinc finger protein 764
9821	RB1CC1	RB1-inducible coiled-coil 1
23208	SYT11	synaptotagmin XI
51312	SLC25A37	solute carrier family 25, member 37
84957	RELT	RELT tumor necrosis factor receptor
114548	NLRP3	NLR family, pyrin domain containing 3
6051	RNPEP	arginyl aminopeptidase (aminopeptidase B)
84222	TMEM191A	transmembrane protein 191A
118472	ZNF511	zinc finger protein 511
220929	ZNF438	zinc finger protein 438
87178	PNPT1	polyribonucleotide nucleotidyltransferase 1
7009	TMBIM6	transmembrane BAX inhibitor motif containing 6
6778	STAT6	signal transducer and activator of transcription 6, interleukin-4 induced
9990	SLC12A6	solute carrier family 12 (potassium/chloride transporters), member 6
23315	SLC9A8	solute carrier family 9 (sodium/hydrogen exchanger), member 8
5987	TRIM27	tripartite motif containing 27
994	CDC25B	cell division cycle 25 homolog B (S. pombe)
113791	PIK3IP1	phosphoinositide-3-kinase interacting protein 1
112616	CMTM7	CKLF-like MARVEL transmembrane domain containing 7
22863	ATG14	ATG14 autophagy related 14 homolog (S. cerevisiae)
26030	PLEKHG3	pleckstrin homology domain containing, family G (with RhoGef domain) member 3
3074	HEXB	hexosaminidase B (beta polypeptide)
55008	HERC6	hect domain and RLD 6
64784	CRTC3	CREB regulated transcription coactivator 3
4277	MICB	MHC class I polypeptide-related sequence B
54210	TREM1	triggering receptor expressed on myeloid cells 1
2526	FUT4	fucosyltransferase 4 (alpha (1,3) fucosyltransferase, myeloid-specific)
93323	HAUS8	HAUS augmin-like complex, subunit 8
3920	LAMP2	lysosomal-associated membrane protein 2
441251	SPDYE7P	speedy homolog E7 (Xenopus laevis), pseudogene
10068	IL18BP	interleukin 18 binding protein
57658	CALCOCO1	calcium binding and coiled-coil domain 1
55270	NUDT15	nudix (nucleoside diphosphate linked moiety X)-type motif 15
22861	NLRP1	NLR family, pyrin domain containing 1
5595	MAPK3	mitogen-activated protein kinase 3
6388	SDF2	stromal cell-derived factor 2
57532	NUFIP2	nuclear fragile X mental retardation protein interacting protein 2
23585	TMEM50A	transmembrane protein 50A
54464	XRN1	5'-3' exoribonuclease 1
7226	TRPM2	transient receptor potential cation channel, subfamily M, member 2
55145	THAP1	THAP domain containing, apoptosis associated protein 1
1982	EIF4G2	eukaryotic translation initiation factor 4 gamma, 2
1486	CTBS	chitobiase, di-N-acetyl-
60682	SMAP1	small ArfGAP 1
7728	ZNF175	zinc finger protein 175
221322	C6orf170	chromosome 6 open reading frame 170
79137	FAM134A	family with sequence similarity 134, member A
23608	MKRN1	makorin ring finger protein 1
9397	NMT2	N-myristoyltransferase 2
23167	EFR3A	EFR3 homolog A (S. cerevisiae)
197335	WDR90	WD repeat domain 90
116988	AGAP3	ArfGAP with GTPase domain, ankyrin repeat and PH domain 3
6622	SNCA	synuclein, alpha (non A4 component of amyloid precursor)
585	BBS4	Bardet-Biedl syndrome 4
114826	SMYD4	SET and MYND domain containing 4
10307	APBB3	amyloid beta (A4) precursor protein-binding, family B, member 3
10634	GAS2L1	growth arrest-specific 2 like 1
8495	PPFIBP2	PTPRF interacting protein, binding protein 2 (liprin beta 2)
10589	DRAP1	DR1-associated protein 1 (negative cofactor 2 alpha)
8497	PPFIA4	protein tyrosine phosphatase, receptor type, f polypeptide (PTPRF), interacting protein (liprin), alpha 4
221496	LEMD2	LEM domain containing 2
197257	LDHD	lactate dehydrogenase D
63922	CHTF18	CTF18, chromosome transmission fidelity factor 18 homolog (S. cerevisiae)
65010	SLC26A6	solute carrier family 26, member 6
255919	TMEM188	transmembrane protein 188
399761	BMS1P5	BMS1 pseudogene 5
8714	ABCC3	ATP-binding cassette, sub-family C (CFTR/MRP), member 3
643418	LIPN	lipase, family member N
91543	RSAD2	radical S-adenosyl methionine domain containing 2
63929	XPNPEP3	X-prolyl aminopeptidase (aminopeptidase P) 3, putative
5222	PGA5	pepsinogen 5, group I (pepsinogen A)
56181	FAM54B	family with sequence similarity 54, member B

7248	TSC1	tuberous sclerosis 1
6617	SNAPC1	small nuclear RNA activating complex, polypeptide 1, 43kDa
492311	C5orf53	chromosome 5 open reading frame 53
2629	GBA	glucosidase, beta, acid
284422	C19orf77	chromosome 19 open reading frame 77
54622	ARL15	ADP-ribosylation factor-like 15
84892	C3orf39	chromosome 3 open reading frame 39
55238	SLC38A7	solute carrier family 38, member 7
1030	CDKN2B	cyclin-dependent kinase inhibitor 2B (p15, inhibits CDK4)
9592	IER2	immediate early response 2
148932	MOBKL2C	MOB1, Mps One Binder kinase activator-like 2C (yeast)
80129	C6orf97	chromosome 6 open reading frame 97
151636	DTX3L	deltex 3-like (Drosophila)
747	DAGLA	diacylglycerol lipase, alpha
5621	PRNP	prion protein
55603	FAM46A	family with sequence similarity 46, member A
401082	FLJ25363	hypothetical LOC401082
64072	CDH23	cadherin-related 23
79847	TMEM180	transmembrane protein 180
134510	UBLCP1	ubiquitin-like domain containing CTD phosphatase 1
51305	KCNK9	potassium channel, subfamily K, member 9
375061	FAM89A	family with sequence similarity 89, member A
56257	MEPCE	methylphosphate capping enzyme
64764	CREB3L2	cAMP responsive element binding protein 3-like 2
84572	GNPTG	N-acetylglucosamine-1-phosphate transferase, gamma subunit
4744	NEFH	neurofilament, heavy polypeptide
51280	GOLM1	golgi membrane protein 1
79992	NCRNA00241	non-protein coding RNA 241
8455	ATRN	attractin
197259	MLKL	mixed lineage kinase domain-like
8685	MARCO	macrophage receptor with collagenous structure
22821	RASA3	RAS p21 protein activator 3
9129	PRPF3	PRP3 pre-mRNA processing factor 3 homolog (S. cerevisiae)
51714	SELT	selenoprotein T
388	RHOB	ras homolog gene family, member B
28959	TMEM176B	transmembrane protein 176B
7408	VASP	vasodilator-stimulated phosphoprotein
838	CASP5	caspase 5, apoptosis-related cysteine peptidase
100131607	LOC100131607	hypothetical LOC100131607
8843	GPR109B	G protein-coupled receptor 109B
285533	RNF175	ring finger protein 175
57719	ANO8	anoctamin 8
8638	OASL	2'-5'-oligoadenylate synthetase-like
719	C3AR1	complement component 3a receptor 1
23523	CABIN1	calcineurin binding protein 1
9853	RUSC2	RUN and SH3 domain containing 2
474338	SUMO1P3	SUMO1 pseudogene 3
5908	RAP1B	RAP1B, member of RAS oncogene family
1513	CTSK	cathepsin K
3382	ICA1	islet cell autoantigen 1, 69kDa
83931	STK40	serine/threonine kinase 40
28966	SNX24	sorting nexin 24
360132	FKBP9L	FK506 binding protein 9-like
22919	MAPRE1	microtubule-associated protein, RP/EB family, member 1
6583	SLC22A4	solute carrier family 22 (organic cation/ergothioneine transporter), member 4
6398	SECTM1	secreted and transmembrane 1
641649	TMEM91	transmembrane protein 91
23405	DICER1	dicer 1, ribonuclease type III
5768	QSOX1	quiescin Q6 sulfhydryl oxidase 1
55723	ASF1B	ASF1 anti-silencing function 1 homolog B (S. cerevisiae)
51538	ZCCHC17	zinc finger, CCHC domain containing 17
85403	EAF1	ELL associated factor 1
79888	LPCAT1	lysophosphatidylcholine acyltransferase 1
84498	FAM120B	family with sequence similarity 120B
84446	BRSK1	BR serine/threonine kinase 1
221786	FAM200A	family with sequence similarity 200, member A
7936	RDBP	RD RNA binding protein
1509	CTSD	cathepsin D
10435	CDC42EP2	CDC42 effector protein (Rho GTPase binding) 2
60412	EXOC4	exocyst complex component 4
54149	C21orf91	chromosome 21 open reading frame 91
5140	PDE3B	phosphodiesterase 3B, cGMP-inhibited
199675	C19orf59	chromosome 19 open reading frame 59
150365	MEI1	meiosis inhibitor 1
7060	THBS4	thrombospondin 4
116372	LYPD1	LY6/PLAUR domain containing 1
1958	EGR1	early growth response 1
5914	RARA	retinoic acid receptor, alpha
53834	FGFRL1	fibroblast growth factor receptor-like 1
9296	ATP6V1F	ATPase, H+ transporting, lysosomal 14kDa, V1 subunit F
284	ANGPT1	angiopoietin 1
7732	RNF112	ring finger protein 112
25946	ZNF385A	zinc finger protein 385A
5708	PSMD2	proteasome (prosome, macropain) 26S subunit, non-ATPase, 2
9890	LPPR4	lipid phosphate phosphatase-related protein type 4
165530	CLEC4F	C-type lectin domain family 4, member F
25903	OLFML2B	olfactomedin-like 2B
944	TNFSF8	tumor necrosis factor (ligand) superfamily, member 8
5873	RAB27A	RAB27A, member RAS oncogene family
149018	LELP1	late cornified envelope-like proline-rich 1
116842	LEAP2	liver expressed antimicrobial peptide 2
4094	MAF	v-maf musculoaponeurotic fibrosarcoma oncogene homolog (avian)
57827	C6orf47	chromosome 6 open reading frame 47
123	PLIN2	perilipin 2
27202	GPR77	G protein-coupled receptor 77

2162	F13A1	coagulation factor XIII, A1 polypeptide
83478	ARHGAP24	Rho GTPase activating protein 24
717	C2	complement component 2
80139	ZNF703	zinc finger protein 703
84179	MFSD7	major facilitator superfamily domain containing 7
1066	CES1	carboxylesterase 1
5783	PTPN13	protein tyrosine phosphatase, non-receptor type 13 (APO-1/CD95 (Fas)-associated phosphatase)
286333	NCRNA00256A	non-protein coding RNA 256A
2350	FOLR2	folate receptor 2 (fetal)
2213	FCGR2B	Fc fragment of IgG, low affinity IIb, receptor (CD32)
9034	CCRL2	chemokine (C-C motif) receptor-like 2
23474	ETHE1	ethylmalonic encephalopathy 1
90362	FAM110B	family with sequence similarity 110, member B
5585	PKN1	protein kinase N1
285550	FAM200B	family with sequence similarity 200, member B
3753	KCNE1	potassium voltage-gated channel, Isk-related family, member 1
54463	FAM134B	family with sequence similarity 134, member B
79852	EPHX3	epoxide hydrolase 3
79842	ZBTB3	zinc finger and BTB domain containing 3
6840	SVIL	supervillin
5806	PTX3	pentraxin 3, long
116443	GRIN3A	glutamate receptor, ionotropic, N-methyl-D-aspartate 3A
23328	SASH1	SAM and SH3 domain containing 1
7464	CORO2A	coronin, actin binding protein, 2A
83787	ARMC10	armadillo repeat containing 10
65124	ANKRD57	ankyrin repeat domain 57
122402	TDRD9	tudor domain containing 9
100132417	FCGR1C	Fc fragment of IgG, high affinity Ic, receptor (CD64)
4481	MSR1	macrophage scavenger receptor 1
339390	CLEC4G	C-type lectin domain family 4, member G
23639	LRRC6	leucine rich repeat containing 6
7106	TSPAN4	tetraspanin 4
53826	FXYD6	FXYD domain containing ion transport regulator 6
657	BMPR1A	bone morphogenetic protein receptor, type IA
1762	DMWD	dystrophia myotonica, WD repeat containing
10462	CLEC10A	C-type lectin domain family 10, member A
89958	C9orf140	chromosome 9 open reading frame 140
222962	SLC29A4	solute carrier family 29 (nucleoside transporters), member 4
2857	GPR34	G protein-coupled receptor 34
9332	CD163	CD163 molecule
10544	PROCR	protein C receptor, endothelial
117144	CATSPER1	cation channel, sperm associated 1
712	C1QA	complement component 1, q subcomponent, A chain
9454	HOMER3	homer homolog 3 (Drosophila)
23166	STAB1	stabilin 1
326342	EMR4P	egf-like module containing, mucin-like, hormone receptor-like 4 pseudogene
55615	PRR5	proline rich 5 (renal)
84668	FAM126A	family with sequence similarity 126, member A
10855	HPSE	heparanase
1959	EGR2	early growth response 2
4023	LPL	lipoprotein lipase
4923	NTSR1	neurotensin receptor 1 (high affinity)
151011	SEPT10	septin 10
165140	OXER1	oxoeicosanoid (OXE) receptor 1
11326	VSIG4	V-set and immunoglobulin domain containing 4
10461	MERTK	c-mer proto-oncogene tyrosine kinase
79605	PGBD5	piggyBac transposable element derived 5
913	CD1E	CD1e molecule
4640	MYO1A	myosin IA
910	CD1B	CD1b molecule
1955	MEGF9	multiple EGF-like-domains 9
2048	EPHB2	EPH receptor B2
3240	HP	haptoglobin
5337	PLD1	phospholipase D1, phosphatidylcholine-specific
653509	SFTPA1	surfactant protein A1
57010	CABP4	calcium binding protein 4
100128059	LOC100128059	hypothetical LOC100128059
653567	FAM23A	family with sequence similarity 23, member A
125206	SLC5A10	solute carrier family 5 (sodium/glucose cotransporter), member 10
10570	DPYSL4	dihydropyrimidinase-like 4
30835	CD209	CD209 molecule
51760	SYT17	synaptotagmin XVII
79971	WLS	wntless homolog (Drosophila)
1593	CYP27A1	cytochrome P450, family 27, subfamily A, polypeptide 1
10911	UTS2	urotensin 2
56300	IL1F9	interleukin 1 family, member 9
5360	PLTP	phospholipid transfer protein
118932	ANKRD22	ankyrin repeat domain 22
6355	CCL8	chemokine (C-C motif) ligand 8
283316	CD163L1	CD163 molecule-like 1

CD1c lineage (upregulated genes)

ENTREZ ID	Gene Symbol	Gene Name
913	CD1E	CD1e molecule
2205	FCER1A	Fc fragment of IgE, high affinity I, receptor for; alpha polypeptide
129293	C2orf89	chromosome 2 open reading frame 89
1235	CCR6	chemokine (C-C motif) receptor 6
911	CD1C	CD1c molecule
64798	DEPTOR	DEP domain containing MTOR-interacting protein
54733	SLC35F2	solute carrier family 35, member F2
5475	PPEF1	protein phosphatase, EF-hand calcium binding domain 1
130589	GALM	galactose mutarotase (aldose 1-epimerase)
1298	COL9A2	collagen, type IX, alpha 2
28951	TRIB2	tribbles homolog 2 (Drosophila)
910	CD1B	CD1b molecule

3516	RBPJ	recombination signal binding protein for immunoglobulin kappa J region
914	CD2	CD2 molecule
143941	TTC36	tetratricopeptide repeat domain 36
60468	BACH2	BTB and CNC homology 1, basic leucine zipper transcription factor 2
9185	REPS2	RALBP1 associated Eps domain containing 2
81539	SLC38A1	solute carrier family 38, member 1
3111	HLA-DOA	major histocompatibility complex, class II, DO alpha
909	CD1A	CD1a molecule
5993	RFX5	regulatory factor X, 5 (influences HLA class II expression)
3581	IL9R	interleukin 9 receptor
26996	GPR160	G protein-coupled receptor 160
9133	CCNB2	cyclin B2
55665	URGCP	upregulator of cell proliferation
201134	CCDC46	coiled-coil domain containing 46
10090	UST	uronyl-2-sulfotransferase
2535	FZD2	frizzled homolog 2 (Drosophila)
921	CD5	CD5 molecule
3784	KCNQ1	potassium voltage-gated channel, KQT-like subfamily, member 1
1107	CHD3	chromodomain helicase DNA binding protein 3
5578	PRKCA	protein kinase C, alpha
8841	HDAC3	histone deacetylase 3
51611	DPH5	DPH5 homolog (S. cerevisiae)
85440	DOCK7	dedicator of cytokinesis 7
2322	FLT3	fms-related tyrosine kinase 3
6209	RPS15	ribosomal protein S15
2675	GFRA2	GDNF family receptor alpha 2
56271	BEX4	brain expressed, X-linked 4
9744	ACAP1	ArfGAP with coiled-coil, ankyrin repeat and PH domains 1
113675	SDSL	serine dehydratase-like
85012	TCEAL3	transcription elongation factor A (SII)-like 3
493	ATP2B4	ATPase, Ca++ transporting, plasma membrane 4
5009	OTC	ornithine carbamoyltransferase
5445	PON2	paraoxonase 2
860	RUNX2	runt-related transcription factor 2
9404	LPXN	leupaxin
80221	ACSF2	acyl-CoA synthetase family member 2
2037	EPB41L2	erythrocyte membrane protein band 4.1-like 2
23231	SEL1L3	sel-1 suppressor of lin-12-like 3 (C. elegans)
64077	LHPP	phospholysine phosphohistidine inorganic pyrophosphate phosphatase
339344	MYPOP	Myb-related transcription factor, partner of profilin
30011	SH3KBP1	SH3-domain kinase binding protein 1
2665	GDI2	GDP dissociation inhibitor 2
6621	SNAPC4	small nuclear RNA activating complex, polypeptide 4, 190kDa
7263	TST	thiosulfate sulfurtransferase (rhodanese)
51522	TMEM14C	transmembrane protein 14C
6252	RTN1	reticulon 1
5305	PIP4K2A	phosphatidylinositol-5-phosphate 4-kinase, type II, alpha
80347	COASY	CoA synthase
5296	PIK3R2	phosphoinositide-3-kinase, regulatory subunit 2 (beta)

CD1c lineage (downregulated genes)

ENTREZ ID	Gene Symbol	Gene Name
5732	PTGER2	prostaglandin E receptor 2 (subtype EP2), 53kDa
341405	ANKRD33	ankyrin repeat domain 33
968	CD68	CD68 molecule
29948	OSGIN1	oxidative stress induced growth inhibitor 1
79443	FYCO1	FYVE and coiled-coil domain containing 1
2030	SLC29A1	solute carrier family 29 (nucleoside transporters), member 1
25798	BRI3	brain protein I3
220929	ZNF438	zinc finger protein 438
23303	KIF13B	kinesin family member 13B
145389	SLC38A6	solute carrier family 38, member 6
57616	TSHZ3	teashirt zinc finger homeobox 3
10924	SMPDL3A	sphingomyelin phosphodiesterase, acid-like 3A
80896	NPL	N-acetylneuraminate pyruvate lyase (dihydrodipicolinate synthase)
171586	ABHD3	abhydrolase domain containing 3
3759	KCNJ2	potassium inwardly-rectifying channel, subfamily J, member 2
8459	TPST2	tyrosylprotein sulfotransferase 2
5341	PLEK	pleckstrin
56851	C15orf24	chromosome 15 open reading frame 24
112770	C1orf85	chromosome 1 open reading frame 85
27348	TOR1B	torsin family 1, member B (torsin B)
11319	ECD	ecdysoneless homolog (Drosophila)
2317	FLNB	filamin B, beta
57192	MCOLN1	mucolipin 1
161742	SPRED1	sprouty-related, EVH1 domain containing 1
666	BOK	BCL2-related ovarian killer
7099	TLR4	toll-like receptor 4
55829	SELS	selenoprotein S
29115	SAP30BP	SAP30 binding protein
55357	TBC1D2	TBC1 domain family, member 2
23111	SPG20	spastic paraplegia 20 (Troyer syndrome)
152687	ZNF595	zinc finger protein 595
9871	SEC24D	SEC24 family, member D (S. cerevisiae)
25832	NBPF14	neuroblastoma breakpoint family, member 14
5222	PGA5	pepsinogen 5, group I (pepsinogen A)
5660	PSAP	prosaposin
23590	PDSS1	prenyl (decaprenyl) diphosphate synthase, subunit 1
8674	VAMP4	vesicle-associated membrane protein 4
50651	SLC45A1	solute carrier family 45, member 1
64795	RMND5A	required for meiotic nuclear division 5 homolog A (S. cerevisiae)
57106	NAT14	N-acetyltransferase 14 (GCN5-related, putative)
91012	LASS5	LAG1 homolog, ceramide synthase 5
51280	GOLM1	golgi membrane protein 1
83693	HSDL1	hydroxysteroid dehydrogenase like 1

4883	NPR3	natriuretic peptide receptor C/guanylate cyclase C (atrionatriuretic peptide receptor C)
11156	PTP4A3	protein tyrosine phosphatase type IVA, member 3
8869	ST3GAL5	ST3 beta-galactoside alpha-2,3-sialyltransferase 5
2766	GMPR	guanosine monophosphate reductase
9601	PDIA4	protein disulfide isomerase family A, member 4
224	ALDH3A2	aldehyde dehydrogenase 3 family, member A2
9554	SEC22B	SEC22 vesicle trafficking protein homolog B (S. cerevisiae) (gene/pseudogene)
9935	MAFB	v-maf musculoaponeurotic fibrosarcoma oncogene homolog B (avian)
51714	SELT	selenoprotein T
113791	PIK3IP1	phosphoinositide-3-kinase interacting protein 1
55711	FAR2	fatty acyl CoA reductase 2
2931	GSK3A	glycogen synthase kinase 3 alpha
643418	LIPN	lipase, family member N
26873	OPLAH	5-oxoprolinase (ATP-hydrolysing)
6556	SLC11A1	solute carrier family 11 (proton-coupled divalent metal ion transporters), member 1
57486	NLN	neurolysin (metallopeptidase M3 family)
1509	CTSD	cathepsin D
3688	ITGB1	integrin, beta 1 (fibronectin receptor, beta polypeptide, antigen CD29 includes MDF2, MSK12)
55723	ASF1B	ASF1 anti-silencing function 1 homolog B (S. cerevisiae)
27202	GPR77	G protein-coupled receptor 77
28966	SNX24	sorting nexin 24
4651	MYO10	myosin X
28959	TMEM176B	transmembrane protein 176B
9853	RUSC2	RUN and SH3 domain containing 2
710	SERPING1	serpin peptidase inhibitor, clade G (C1 inhibitor), member 1
84446	BRSK1	BR serine/threonine kinase 1
8269	TMEM187	transmembrane protein 187
57507	ZNF608	zinc finger protein 608
7704	ZBTB16	zinc finger and BTB domain containing 16
10184	LHFPL2	lipoma HMGIC fusion partner-like 2
80139	ZNF703	zinc finger protein 703
148741	ANKRD35	ankyrin repeat domain 35
23328	SASH1	SAM and SH3 domain containing 1
5335	PLCG1	phospholipase C, gamma 1
151011	SEPT10	septin 10
2048	EPHB2	EPH receptor B2
90362	FAM110B	family with sequence similarity 110, member B
717	C2	complement component 2
80183	C13orf18	chromosome 13 open reading frame 18
10461	MERTK	c-mer proto-oncogene tyrosine kinase
25903	OLFML2B	olfactomedin-like 2B
65124	ANKRD57	ankyrin repeat domain 57
5064	PALM	paralemmin
1906	EDN1	endothelin 1
10855	HPSE	heparanase
719	C3AR1	complement component 3a receptor 1
79412	KREMEN2	kringle containing transmembrane protein 2
5368	PNOC	prepronociceptin
3904	LAIR2	leukocyte-associated immunoglobulin-like receptor 2
79605	PGBD5	piggyBac transposable element derived 5
1501	CTNND2	catenin (cadherin-associated protein), delta 2 (neural plakophilin-related arm-repeat protein)
160335	TMTC2	transmembrane and tetratricopeptide repeat containing 2
5159	PDGFRB	platelet-derived growth factor receptor, beta polypeptide
9454	HOMER3	homer homolog 3 (Drosophila)
1903	S1PR3	sphingosine-1-phosphate receptor 3
147495	APCDD1	adenomatosis polyposis coli down-regulated 1
55384	MEG3	maternally expressed 3 (non-protein coding)
440712	C1orf186	chromosome 1 open reading frame 186
83716	CRISPLD2	cysteine-rich secretory protein LCCL domain containing 2
5167	ENPP1	ectonucleotide pyrophosphatase/phosphodiesterase 1
7450	VWF	von Willebrand factor
1803	DPP4	dipeptidyl-peptidase 4
5337	PLD1	phospholipase D1, phosphatidylcholine-specific
5730	PTGDS	prostaglandin D2 synthase 21kDa (brain)
116159	CYYR1	cysteine/tyrosine-rich 1

CD14+ skin DC (upregulated genes)

ENTREZ ID	Gene Symbol	Gene Name
9446	GSTO1	glutathione S-transferase omega 1
30835	CD209	CD209 molecule
10062	NR1H3	nuclear receptor subfamily 1, group H, member 3
585	BBS4	Bardet-Biedl syndrome 4
7226	TRPM2	transient receptor potential cation channel, subfamily M, member 2
65010	SLC26A6	solute carrier family 26, member 6
57583	TMEM181	transmembrane protein 181
24149	ZNF318	zinc finger protein 318
10184	LHFPL2	lipoma HMGIC fusion partner-like 2
145389	SLC38A6	solute carrier family 38, member 6
25903	OLFML2B	olfactomedin-like 2B
80765	STARD5	StAR-related lipid transfer (START) domain containing 5
65124	ANKRD57	ankyrin repeat domain 57
123	PLIN2	perilipin 2
53831	GPR84	G protein-coupled receptor 84
4860	PNP	purine nucleoside phosphorylase
63875	MRPL17	mitochondrial ribosomal protein L17
537	ATP6AP1	ATPase, H+ transporting, lysosomal accessory protein 1
401321	LOC401321	hypothetical LOC401321
9296	ATP6V1F	ATPase, H+ transporting, lysosomal 14kDa, V1 subunit F
7106	TSPAN4	tetraspanin 4
23646	PLD3	phospholipase D family, member 3
57669	EPB41L5	erythrocyte membrane protein band 4.1 like 5
161742	SPRED1	sprouty-related, EVH1 domain containing 1
116985	ARAP1	ArfGAP with RhoGAP domain, ankyrin repeat and PH domain 1
5768	QSOX1	quiescin Q6 sulfhydryl oxidase 1

10307	APBB3	amyloid beta (A4) precursor protein-binding, family B, member 3
492311	C5orf53	chromosome 5 open reading frame 53
84879	MFSD2A	major facilitator superfamily domain containing 2A
30827	CXXC1	CXXC finger protein 1
80896	NPL	N-acetylneuraminate pyruvate lyase (dihydrodipicolinate synthase)
7466	WFS1	Wolfram syndrome 1 (wolframin)
5337	PLD1	phospholipase D1, phosphatidylcholine-specific
55353	LAPTM4B	lysosomal protein transmembrane 4 beta
1603	DAD1	defender against cell death 1
283316	CD163L1	CD163 molecule-like 1
1837	DTNA	dystrobrevin, alpha
9404	LPXN	leupaxin
5660	PSAP	prosaposin
3783	KCNN4	potassium intermediate/small conductance calcium-activated channel, subfamily N, member 4
9454	HOMER3	homer homolog 3 (Drosophila)
360132	FKBP9L	FK506 binding protein 9-like
9853	RUSC2	RUN and SH3 domain containing 2
9935	MAFB	v-maf musculoaponeurotic fibrosarcoma oncogene homolog B (avian)
200030	NBPF11	neuroblastoma breakpoint family, member 11
93663	ARHGAP18	Rho GTPase activating protein 18
29100	TMEM208	transmembrane protein 208
666	BOK	BCL2-related ovarian killer
85012	TCEAL3	transcription elongation factor A (SII)-like 3
8714	ABCC3	ATP-binding cassette, sub-family C (CFTR/MRP), member 3
4245	MGAT1	mannosyl (alpha-1,3-)-glycoprotein beta-1,2-N-acetylglucosaminyltransferase
719	C3AR1	complement component 3a receptor 1
79921	TCEAL4	transcription elongation factor A (SII)-like 4
84446	BRSK1	BR serine/threonine kinase 1
1803	DPP4	dipeptidyl-peptidase 4
79567	FAM65A	family with sequence similarity 65, member A
27018	NGFRAP1	nerve growth factor receptor (TNFRSF16) associated protein 1
8605	PLA2G4C	phospholipase A2, group IVC (cytosolic, calcium-independent)
341405	ANKRD33	ankyrin repeat domain 33
55365	TMEM176A	transmembrane protein 176A
929	CD14	CD14 molecule
1601	DAB2	disabled homolog 2, mitogen-responsive phosphoprotein (Drosophila)
51309	ARMCX1	armadillo repeat containing, X-linked 1
3964	LGALS8	lectin, galactoside-binding, soluble, 8
55756	INTS9	integrator complex subunit 9
55665	URGCP	upregulator of cell proliferation
8706	B3GALNT1	beta-1,3-N-acetylgalactosaminyltransferase 1 (globoside blood group)
29948	OSGIN1	oxidative stress induced growth inhibitor 1
64218	SEMA4A	sema domain, immunoglobulin domain (Ig), transmembrane domain (TM) and short cytoplasmic domain, (semaphorin) 4A
2040	STOM	stomatin
200558	APLF	aprataxin and PNKP like factor
6774	STAT3	signal transducer and activator of transcription 3 (acute-phase response factor)
2055	CLN8	ceroid-lipofuscinosis, neuronal 8 (epilepsy, progressive with mental retardation)
203069	R3HCC1	R3H domain and coiled-coil containing 1
27151	CPAMD8	C3 and PZP-like, alpha-2-macroglobulin domain containing 8
1508	CTSB	cathepsin B
10863	ADAM28	ADAM metallopeptidase domain 28
374882	TMEM205	transmembrane protein 205
112770	C1orf85	chromosome 1 open reading frame 85
23357	ANGEL1	angel homolog 1 (Drosophila)
221496	LEMD2	LEM domain containing 2
55051	C14orf102	chromosome 14 open reading frame 102
3311	HSPA7	heat shock 70kDa protein 7 (HSP70B)
1030	CDKN2B	cyclin-dependent kinase inhibitor 2B (p15, inhibits CDK4)
57514	ARHGAP31	Rho GTPase activating protein 31
3074	HEXB	hexosaminidase B (beta polypeptide)
8674	VAMP4	vesicle-associated membrane protein 4
11188	NISCH	nischarin
374395	TMEM179B	transmembrane protein 179B
339344	MYPOP	Myb-related transcription factor, partner of profilin
755	C21orf2	chromosome 21 open reading frame 2
84875	PARP10	poly (ADP-ribose) polymerase family, member 10
84193	SETD3	SET domain containing 3
9637	FEZ2	fasciculation and elongation protein zeta 2 (zygin II)
10312	TCIRG1	T-cell, immune regulator 1, ATPase, H+ transporting, lysosomal V0 subunit A3
127066	OR14C36	olfactory receptor, family 14, subfamily C, member 36
5732	PTGER2	prostaglandin E receptor 2 (subtype EP2), 53kDa
144402	CPNE8	copine VIII
64411	ARAP3	ArfGAP with RhoGAP domain, ankyrin repeat and PH domain 3
8943	AP3D1	adaptor-related protein complex 3, delta 1 subunit
527	ATP6V0C	ATPase, H+ transporting, lysosomal 16kDa, V0 subunit c
140467	ZNF358	zinc finger protein 358
26175	C14orf109	chromosome 14 open reading frame 109
4126	MANBA	mannosidase, beta A, lysosomal
63906	GPATCH3	G patch domain containing 3
54453	RIN2	Ras and Rab interactor 2
29992	PILRA	paired immunoglobin-like type 2 receptor alpha
85450	ITPRIP	inositol 1,4,5-triphosphate receptor interacting protein
53	ACP2	acid phosphatase 2, lysosomal
6453	ITSN1	intersectin 1 (SH3 domain protein)
1793	DOCK1	dedicator of cytokinesis 1
54103	PION	pigeon homolog (Drosophila)
10985	GCN1L1	GCN1 general control of amino-acid synthesis 1-like 1 (yeast)
90843	TCEAL8	transcription elongation factor A (SII)-like 8
27128	CYTH4	cytohesin 4
9839	ZEB2	zinc finger E-box binding homeobox 2
2770	GNAI1	guanine nucleotide binding protein (G protein), alpha inhibiting activity polypeptide 1
10455	PECI	peroxisomal D3,D2-enoyl-CoA isomerase
3916	LAMP1	lysosomal-associated membrane protein 1
837	CASP4	caspase 4, apoptosis-related cysteine peptidase
11213	IRAK3	interleukin-1 receptor-associated kinase 3

22918	CD93	CD93 molecule
116832	RPL39L	ribosomal protein L39-like
1051	CEBPB	CCAAT/enhancer binding protein (C/EBP), beta
203054	ADCK5	aarF domain containing kinase 5
54856	GON4L	gon-4-like (C. elegans)
5210	PFKFB4	6-phosphofructo-2-kinase/fructose-2,6-biphosphatase 4
968	CD68	CD68 molecule
8495	PPFIBP2	PTPRF interacting protein, binding protein 2 (liprin beta 2)
55748	CNDP2	CNDP dipeptidase 2 (metallopeptidase M20 family)
79168	LILRA6	leukocyte immunoglobulin-like receptor, subfamily A (with TM domain), member 6
64784	CRTC3	CREB regulated transcription coactivator 3
84034	EMILIN2	elastin microfibril interfacer 2
23247	KIAA0556	KIAA0556
2799	GNS	glucosamine (N-acetyl)-6-sulfatase
10410	IFITM3	interferon induced transmembrane protein 3 (1-8U)
3628	INPP1	inositol polyphosphate-1-phosphatase
100129550	LOC100129550	hypothetical LOC100129550
10581	IFITM2	interferon induced transmembrane protein 2 (1-8D)
79720	VPS37B	vacuolar protein sorting 37 homolog B (S. cerevisiae)
57794	SUGP1	SURP and G patch domain containing 1
51296	SLC15A3	solute carrier family 15, member 3
3987	LIMS1	LIM and senescent cell antigen-like domains 1
1436	CSF1R	colony stimulating factor 1 receptor
11025	LILRB3	leukocyte immunoglobulin-like receptor, subfamily B (with TM and ITIM domains), member 3
9061	PAPSS1	3'-phosphoadenosine 5'-phosphosulfate synthase 1
151888	BTLA	B and T lymphocyte associated
834	CASP1	caspase 1, apoptosis-related cysteine peptidase (interleukin 1, beta, convertase)

CD14+ skin DC (downregulated genes)

ENTREZ ID	Gene Symbol	Gene Name
2021	ENDOG	endonuclease G
10659	CELF2	CUGBP, Elav-like family member 2
3784	KCNQ1	potassium voltage-gated channel, KQT-like subfamily, member 1
6210	RPS15A	ribosomal protein S15a
51187	RSL24D1	ribosomal L24 domain containing 1
51155	HN1	hematological and neurological expressed 1
23354	HAUS5	HAUS augmin-like complex, subunit 5
57326	PBXIP1	pre-B-cell leukemia homeobox interacting protein 1
2322	FLT3	fms-related tyrosine kinase 3
162979	ZNF296	zinc finger protein 296
9185	REPS2	RALBP1 associated Eps domain containing 2
1933	EEF1B2	eukaryotic translation elongation factor 1 beta 2
401647	GOLGA7B	golgin A7 family, member B
2852	GPER	G protein-coupled estrogen receptor 1
158358	KIAA2026	KIAA2026
57599	WDR48	WD repeat domain 48
5981	RFC1	replication factor C (activator 1) 1, 145kDa
26060	APPL1	adaptor protein, phosphotyrosine interaction, PH domain and leucine zipper containing 1
1595	CYP51A1	cytochrome P450, family 51, subfamily A, polypeptide 1
6891	TAP2	transporter 2, ATP-binding cassette, sub-family B (MDR/TAP)
9991	ROD1	ROD1 regulator of differentiation 1 (S. pombe)
4724	NDUFS4	NADH dehydrogenase (ubiquinone) Fe-S protein 4, 18kDa (NADH-coenzyme Q reductase)
493	ATP2B4	ATPase, Ca++ transporting, plasma membrane 4
6207	RPS13	ribosomal protein S13
51056	LAP3	leucine aminopeptidase 3
8850	KAT2B	K(lysine) acetyltransferase 2B
3476	IGBP1	immunoglobulin (CD79A) binding protein 1
55911	APOBR	apolipoprotein B receptor
6421	SFPQ	splicing factor proline/glutamine-rich
51199	NIN	ninein (GSK3B interacting protein)
80145	THOC7	THO complex 7 homolog (Drosophila)
728643	LOC728643	heterogeneous nuclear ribonucleoprotein A1 pseudogene
90990	KIFC2	kinesin family member C2
57591	MKL1	megakaryoblastic leukemia (translocation) 1
118433	RPL23AP7	ribosomal protein L23a pseudogene 7
79791	FBXO31	F-box protein 31
6235	RPS29	ribosomal protein S29
6640	SNTA1	syntrophin, alpha 1 (dystrophin-associated protein A1, 59kDa, acidic component)
23625	FAM89B	family with sequence similarity 89, member B
8099	CDK2AP1	cyclin-dependent kinase 2 associated protein 1
1316	KLF6	Kruppel-like factor 6
10890	RAB10	RAB10, member RAS oncogene family
338707	B4GALNT4	beta-1,4-N-acetyl-galactosaminyl transferase 4
5223	PGAM1	phosphoglycerate mutase 1 (brain)
55915	LANCL2	LanC lantibiotic synthetase component C-like 2 (bacterial)
4627	MYH9	myosin, heavy chain 9, non-muscle
6202	RPS8	ribosomal protein S8
5170	PDPK1	3-phosphoinositide dependent protein kinase-1
5993	RFX5	regulatory factor X, 5 (influences HLA class II expression)
100130613	CXorf64	chromosome X open reading frame 64
51559	NT5DC3	5'-nucleotidase domain containing 3
388574	FLJ43681	ribosomal protein L23a pseudogene
171586	ABHD3	abhydrolase domain containing 3
57106	NAT14	N-acetyltransferase 14 (GCN5-related, putative)
54414	SIAE	sialic acid acetylesterase
348180	CTU2	cytosolic thiouridylase subunit 2 homolog (S. pombe)
909	CD1A	CD1a molecule
9744	ACAP1	ArfGAP with coiled-coil, ankyrin repeat and PH domains 1
157	ADRBK2	adrenergic, beta, receptor kinase 2
5090	PBX3	pre-B-cell leukemia homeobox 3
2729	GCLC	glutamate-cysteine ligase, catalytic subunit
7791	ZYX	zyxin
26750	RPS6KC1	ribosomal protein S6 kinase, 52kDa, polypeptide 1
2529	FUT7	fucosyltransferase 7 (alpha (1,3) fucosyltransferase)
10627	MYL12A	myosin, light chain 12A, regulatory, non-sarcomeric

1633	DCK	deoxycytidine kinase
54149	C21orf91	chromosome 21 open reading frame 91
6050	RNH1	ribonuclease/angiogenin inhibitor 1
6164	RPL34	ribosomal protein L34
23157	SEPT6	septin 6
2534	FYN	FYN oncogene related to SRC, FGR, YES
23240	KIAA0922	KIAA0922
8869	ST3GAL5	ST3 beta-galactoside alpha-2,3-sialyltransferase 5
3338	DNAJC4	DnaJ (Hsp40) homolog, subfamily C, member 4
57185	NIPAL3	NIPA-like domain containing 3
3422	IDI1	isopentenyl-diphosphate delta isomerase 1
2788	GNG7	guanine nucleotide binding protein (G protein), gamma 7
55300	PI4K2B	phosphatidylinositol 4-kinase type 2 beta
57579	FAM135A	family with sequence similarity 135, member A
729786	GOLGA8C	golgin A8 family, member C
132720	C4orf32	chromosome 4 open reading frame 32
8622	PDE8B	phosphodiesterase 8B
60	ACTB	actin, beta
11138	TBC1D8	TBC1 domain family, member 8 (with GRAM domain)
8934	RAB7L1	RAB7, member RAS oncogene family-like 1
22955	SCMH1	sex comb on midleg homolog 1 (Drosophila)
81539	SLC38A1	solute carrier family 38, member 1
57037	ANKMY2	ankyrin repeat and MYND domain containing 2
79705	LRRK1	leucine-rich repeat kinase 1
152559	PAQR3	progestin and adipoQ receptor family member III
5664	PSEN2	presenilin 2 (Alzheimer disease 4)
166614	DCLK2	doublecortin-like kinase 2
728743	LOC728743	similar to GLI-Kruppel family member HKR1
23483	TGDS	TDP-glucose 4,6-dehydratase
160335	TMTC2	transmembrane and tetratricopeptide repeat containing 2
2766	GMPR	guanosine monophosphate reductase
55266	TMEM19	transmembrane protein 19
81552	VOPP1	vesicular, overexpressed in cancer, prosurvival protein 1
4065	LY75	lymphocyte antigen 75
79630	C1orf54	chromosome 1 open reading frame 54
25873	RPL36	ribosomal protein L36
165530	CLEC4F	C-type lectin domain family 4, member F
921	CD5	CD5 molecule
84057	MND1	meiotic nuclear divisions 1 homolog (S. cerevisiae)
89958	C9orf140	chromosome 9 open reading frame 140
220963	SLC16A9	solute carrier family 16, member 9 (monocarboxylic acid transporter 9)
79762	C1orf115	chromosome 1 open reading frame 115
51205	ACP6	acid phosphatase 6, lysophosphatidic
81606	LBH	limb bud and heart development homolog (mouse)
4810	NHS	Nance-Horan syndrome (congenital cataracts and dental anomalies)
55086	CXorf57	chromosome X open reading frame 57
154313	C6orf165	chromosome 6 open reading frame 165
5527	PPP2R5C	protein phosphatase 2, regulatory subunit B', gamma
57705	WDFY4	WDFY family member 4
9760	TOX	thymocyte selection-associated high mobility group box
2833	CXCR3	chemokine (C-X-C motif) receptor 3
100505501	LOC100505501	hypothetical LOC100505501
79656	BEND5	BEN domain containing 5
81620	CDT1	chromatin licensing and DNA replication factor 1
5368	PNOC	prepronociceptin
7984	ARHGEF5	Rho guanine nucleotide exchange factor (GEF) 5
946	SIGLEC6	sialic acid binding Ig-like lectin 6
22874	PLEKHA6	pleckstrin homology domain containing, family A member 6
7710	ZNF154	zinc finger protein 154
2047	EPHB1	EPH receptor B1
9901	SRGAP3	SLIT-ROBO Rho GTPase activating protein 3
84433	CARD11	caspase recruitment domain family, member 11
9806	SPOCK2	sparc/osteonectin, cwcv and kazal-like domains proteoglycan (testican) 2
219902	TMEM136	transmembrane protein 136

CD14+ blood monocytes (upregulated genes)

ENTREZ ID	Gene Symbol	Gene Name
929	CD14	CD14 molecule
83716	CRISPLD2	cysteine-rich secretory protein LCCL domain containing 2
199675	C19orf59	chromosome 19 open reading frame 59
117144	CATSPER1	cation channel, sperm associated 1
1593	CYP27A1	cytochrome P450, family 27, subfamily A, polypeptide 1
10855	HPSE	heparanase
1903	S1PR3	sphingosine-1-phosphate receptor 3
83478	ARHGAP24	Rho GTPase activating protein 24
6583	SLC22A4	solute carrier family 22 (organic cation/ergothioneine transporter), member 4
3176	HNMT	histamine N-methyltransferase
389634	LOC389634	hypothetical LOC389634
666	BOK	BCL2-related ovarian killer
9332	CD163	CD163 molecule
6556	SLC11A1	solute carrier family 11 (proton-coupled divalent metal ion transporters), member 1
53826	FXYD6	FXYD domain containing ion transport regulator 6
150365	MEI1	meiosis inhibitor 1
57616	TSHZ3	teashirt zinc finger homeobox 3
55365	TMEM176A	transmembrane protein 176A
5476	CTSA	cathepsin A
2162	F13A1	coagulation factor XIII, A1 polypeptide
5724	PTAFR	platelet-activating factor receptor
79971	WLS	wntless homolog (Drosophila)
1462	VCAN	versican
9466	IL27RA	interleukin 27 receptor, alpha
641649	TMEM91	transmembrane protein 91
3240	HP	haptoglobin

326	AIRE	autoimmune regulator
1991	ELANE	elastase, neutrophil expressed
10970	CKAP4	cytoskeleton-associated protein 4
285550	FAM200B	family with sequence similarity 200, member B
5595	MAPK3	mitogen-activated protein kinase 3
64411	ARAP3	ArfGAP with RhoGAP domain, ankyrin repeat and PH domain 3
84446	BRSK1	BR serine/threonine kinase 1
4810	NHS	Nance-Horan syndrome (congenital cataracts and dental anomalies)
100132417	FCGR1C	Fc fragment of IgG, high affinity Ic, receptor (CD64)
9853	RUSC2	RUN and SH3 domain containing 2
51734	SEPX1	selenoprotein X, 1
3148	HMGB2	high-mobility group box 2
441251	SPDYE7P	speedy homolog E7 (Xenopus laevis), pseudogene
11152	WDR45	WD repeat domain 45
54540	FAM193B	family with sequence similarity 193, member B
4257	MGST1	microsomal glutathione S-transferase 1
9454	HOMER3	homer homolog 3 (Drosophila)
2054	STX2	syntaxin 2
55357	TBC1D2	TBC1 domain family, member 2
8819	SAP30	Sin3A-associated protein, 30kDa
54210	TREM1	triggering receptor expressed on myeloid cells 1
4999	ORC2	origin recognition complex, subunit 2
4043	LRPAP1	low density lipoprotein receptor-related protein associated protein 1
83719	YPEL3	yippee-like 3 (Drosophila)
80896	NPL	N-acetylneuraminate pyruvate lyase (dihydrodipicolinate synthase)
27151	CPAMD8	C3 and PZP-like, alpha-2-macroglobulin domain containing 8
51280	GOLM1	golgi membrane protein 1
7739	ZNF185	zinc finger protein 185 (LIM domain)
113277	TMEM106A	transmembrane protein 106A
79954	NOL10	nucleolar protein 10
23166	STAB1	stabilin 1
55890	GPRC5C	G protein-coupled receptor, family C, group 5, member C
79939	SLC35E1	solute carrier family 35, member E1
360132	FKBP9L	FK506 binding protein 9-like
6993	DYNLT1	dynein, light chain, Tctex-type 1
151011	SEPT10	septin 10
91663	MYADM	myeloid-associated differentiation marker
53346	TM6SF1	transmembrane 6 superfamily member 1
122402	TDRD9	tudor domain containing 9
341405	ANKRD33	ankyrin repeat domain 33
80267	EDEM3	ER degradation enhancer, mannosidase alpha-like 3
23590	PDSS1	prenyl (decaprenyl) diphosphate synthase, subunit 1
401082	FLJ25363	hypothetical LOC401082
28959	TMEM176B	transmembrane protein 176B
3957	LGALS2	lectin, galactoside-binding, soluble, 2
144402	CPNE8	copine VIII
51102	MECR	mitochondrial trans-2-enoyl-CoA reductase
116832	RPL39L	ribosomal protein L39-like
440836	ODF3B	outer dense fiber of sperm tails 3B
3949	LDLR	low density lipoprotein receptor
10205	MPZL2	myelin protein zero-like 2
10144	FAM13A	family with sequence similarity 13, member A
7099	TLR4	toll-like receptor 4
944	TNFSF8	tumor necrosis factor (ligand) superfamily, member 8
10924	SMPDL3A	sphingomyelin phosphodiesterase, acid-like 3A
9935	MAFB	v-maf musculoaponeurotic fibrosarcoma oncogene homolog B (avian)
9821	RB1CC1	RB1-inducible coiled-coil 1
317781	DDX51	DEAD (Asp-Glu-Ala-Asp) box polypeptide 51
1066	CES1	carboxylesterase 1
339390	CLEC4G	C-type lectin domain family 4, member G
9592	IER2	immediate early response 2
121551	BTBD11	BTB (POZ) domain containing 11
91750	LIN52	lin-52 homolog (C. elegans)
6540	SLC6A13	solute carrier family 6 (neurotransmitter transporter, GABA), member 13
5768	QSOX1	quiescin Q6 sulfhydryl oxidase 1
51304	ZDHHC3	zinc finger, DHHC-type containing 3
2048	EPHB2	EPH receptor B2
114785	MBD6	methyl-CpG binding domain protein 6
126014	OSCAR	osteoclast associated, immunoglobulin-like receptor
197259	MLKL	mixed lineage kinase domain-like
6752	SSTR2	somatostatin receptor 2
146223	CMTM4	CKLF-like MARVEL transmembrane domain containing 4
9397	NMT2	N-myristoyltransferase 2
2150	F2RL1	coagulation factor II (thrombin) receptor-like 1
2644	GCHFR	GTP cyclohydrolase I feedback regulator
80867	HCG2P7	HLA complex group 2 pseudogene 7
221662	RBM24	RNA binding motif protein 24
84255	SLC37A3	solute carrier family 37 (glycerol-3-phosphate transporter), member 3
7226	TRPM2	transient receptor potential cation channel, subfamily M, member 2
404636	FAM45A	family with sequence similarity 45, member A
87178	PNPT1	polyribonucleotide nucleotidyltransferase 1
55711	FAR2	fatty acyl CoA reductase 2
51311	TLR8	toll-like receptor 8
11188	NISCH	nischarin
4923	NTSR1	neurotensin receptor 1 (high affinity)
6774	STAT3	signal transducer and activator of transcription 3 (acute-phase response factor)
116988	AGAP3	ArfGAP with GTPase domain, ankyrin repeat and PH domain 3
11270	NRM	nurim (nuclear envelope membrane protein)
374882	TMEM205	transmembrane protein 205
5914	RARA	retinoic acid receptor, alpha
7554	ZNF8	zinc finger protein 8
221037	JMJD1C	jumonji domain containing 1C
137835	TMEM71	transmembrane protein 71
9232	PTTG1	pituitary tumor-transforming 1
54507	ADAMTSL4	ADAMTS-like 4

116092	DNTTIP1	deoxynucleotidyltransferase, terminal, interacting protein 1
3636	INPPL1	inositol polyphosphate phosphatase-like 1
4117	MAK	male germ cell-associated kinase
3074	HEXB	hexosaminidase B (beta polypeptide)
8569	MKNK1	MAP kinase interacting serine/threonine kinase 1
1513	CTSK	cathepsin K
283	ANG	angiogenin, ribonuclease, RNase A family, 5
5873	RAB27A	RAB27A, member RAS oncogene family
26873	OPLAH	5-oxoprolinase (ATP-hydrolysing)
10542	HBXIP	hepatitis B virus x interacting protein
319085	ITPK1-AS1	ITPK1 antisense RNA 1 (non-protein coding)
142679	DUSP19	dual specificity phosphatase 19
55238	SLC38A7	solute carrier family 38, member 7
23514	KIAA0146	KIAA0146
147372	CCBE1	collagen and calcium binding EGF domains 1
1871	E2F3	E2F transcription factor 3
117155	CATSPER2	cation channel, sperm associated 2
11330	CTRC	chymotrypsin C (caldecrin)
285172	FAM126B	family with sequence similarity 126, member B
79992	NCRNA00241	non-protein coding RNA 241
399761	BMS1P5	BMS1 pseudogene 5
54453	RIN2	Ras and Rab interactor 2
93589	CACNA2D4	calcium channel, voltage-dependent, alpha 2/delta subunit 4
27348	TOR1B	torsin family 1, member B (torsin B)
57190	SEPN1	selenoprotein N, 1
374875	HSD11B1L	hydroxysteroid (11-beta) dehydrogenase 1-like
80325	ABTB1	ankyrin repeat and BTB (POZ) domain containing 1
64784	CRTC3	CREB regulated transcription coactivator 3
25798	BRI3	brain protein I3
3189	HNRNPH3	heterogeneous nuclear ribonucleoprotein H3 (2H9)
79847	TMEM180	transmembrane protein 180
7791	ZYX	zyxin
7456	WIPF1	WAS/WASL interacting protein family, member 1
388692	LOC388692	hypothetical LOC388692
64645	HIAT1	hippocampus abundant transcript 1
9051	PSTPIP1	proline-serine-threonine phosphatase interacting protein 1
151636	DTX3L	deltex 3-like (Drosophila)
1138	CHRNA5	cholinergic receptor, nicotinic, alpha 5
607	BCL9	B-cell CLL/lymphoma 9
3241	HPCAL1	hippocalcin-like 1
79042	TSEN34	tRNA splicing endonuclease 34 homolog (S. cerevisiae)
54930	HAUS4	HAUS augmin-like complex, subunit 4
3682	ITGAE	integrin, alpha E (antigen CD103, human mucosal lymphocyte antigen 1; alpha polypeptide)
57477	SHROOM4	shroom family member 4
6548	SLC9A1	solute carrier family 9 (sodium/hydrogen exchanger), member 1
100129550	LOC100129550	hypothetical LOC100129550
5184	PEPD	peptidase D
8455	ATRN	attractin
79168	LILRA6	leukocyte immunoglobulin-like receptor, subfamily A (with TM domain), member 6
4851	NOTCH1	notch 1
84572	GNPTG	N-acetylglucosamine-1-phosphate transferase, gamma subunit
2869	GRK5	G protein-coupled receptor kinase 5
2526	FUT4	fucosyltransferase 4 (alpha (1,3) fucosyltransferase, myeloid-specific)
1102	RCBTB2	regulator of chromosome condensation (RCC1) and BTB (POZ) domain containing protein 2
5337	PLD1	phospholipase D1, phosphatidylcholine-specific
84293	C10orf58	chromosome 10 open reading frame 58
23474	ETHE1	ethylmalonic encephalopathy 1
6453	ITSN1	intersectin 1 (SH3 domain protein)
3305	HSPA1L	heat shock 70kDa protein 1-like
7803	PTP4A1	protein tyrosine phosphatase type IVA, member 1
9997	SCO2	SCO cytochrome oxidase deficient homolog 2 (yeast)
5728	PTEN	phosphatase and tensin homolog
8408	ULK1	unc-51-like kinase 1 (C. elegans)
126272	EID2B	EP300 interacting inhibitor of differentiation 2B
29992	PILRA	paired immunoglobin-like type 2 receptor alpha
80736	SLC44A4	solute carrier family 44, member 4
2876	GPX1	glutathione peroxidase 1
5222	PGA5	pepsinogen 5, group I (pepsinogen A)
1387	CREBBP	CREB binding protein
220929	ZNF438	zinc finger protein 438
4354	MPP1	membrane protein, palmitoylated 1, 55kDa
2131	EXT1	exostosin 1
55625	ZDHHC7	zinc finger, DHHC-type containing 7
474338	SUMO1P3	SUMO1 pseudogene 3
23167	EFR3A	EFR3 homolog A (S. cerevisiae)
79026	AHNAK	AHNAK nucleoprotein
4126	MANBA	mannosidase, beta A, lysosomal
23593	HEBP2	heme binding protein 2
29097	CNIH4	cornichon homolog 4 (Drosophila)
909	CD1A	CD1a molecule
706	TSPO	translocator protein (18kDa)
56895	AGPAT4	1-acylglycerol-3-phosphate O-acyltransferase 4 (lysophosphatidic acid acyltransferase, delta)
9296	ATP6V1F	ATPase, H+ transporting, lysosomal 14kDa, V1 subunit F
196383	RILPL2	Rab interacting lysosomal protein-like 2
3156	HMGCR	3-hydroxy-3-methylglutaryl-CoA reductase
2350	FOLR2	folate receptor 2 (fetal)
26301	GBGT1	globoside alpha-1,3-N-acetylgalactosaminyltransferase 1
57498	KIDINS220	kinase D-interacting substrate, 220kDa
6617	SNAPC1	small nuclear RNA activating complex, polypeptide 1, 43kDa
6778	STAT6	signal transducer and activator of transcription 6, interleukin-4 induced
2885	GRB2	growth factor receptor-bound protein 2
7132	TNFRSF1A	tumor necrosis factor receptor superfamily, member 1A
100506144	LOC100506144	hypothetical protein LOC100506144
51621	KLF13	Kruppel-like factor 13
27239	GPR162	G protein-coupled receptor 162

112616	CMTM7	CKLF-like MARVEL transmembrane domain containing 7
7462	LAT2	linker for activation of T cells family, member 2
138151	NACC2	NACC family member 2, BEN and BTB (POZ) domain containing
6622	SNCA	synuclein, alpha (non A4 component of amyloid precursor)
6277	S100A6	S100 calcium binding protein A6
112770	C1orf85	chromosome 1 open reading frame 85
10673	TNFSF13B	tumor necrosis factor (ligand) superfamily, member 13b
63929	XPNPEP3	X-prolyl aminopeptidase (aminopeptidase P) 3, putative
88455	ANKRD13A	ankyrin repeat domain 13A
58475	MS4A7	membrane-spanning 4-domains, subfamily A, member 7
91351	DDX60L	DEAD (Asp-Glu-Ala-Asp) box polypeptide 60-like
65018	PINK1	PTEN induced putative kinase 1
353514	LILRA5	leukocyte immunoglobulin-like receptor, subfamily A (with TM domain), member 5
55654	TMEM127	transmembrane protein 127
4303	FOXO4	forkhead box O4
55760	DHX32	DEAH (Asp-Glu-Ala-His) box polypeptide 32
114548	NLRP3	NLR family, pyrin domain containing 3
9673	SLC25A44	solute carrier family 25, member 44
8650	NUMB	numb homolog (Drosophila)
4245	MGAT1	mannosyl (alpha-1,3-)-glycoprotein beta-1,2-N-acetylglucosaminyltransferase
8266	UBL4A	ubiquitin-like 4A
29994	BAZ2B	bromodomain adjacent to zinc finger domain, 2B
23585	TMEM50A	transmembrane protein 50A
11320	MGAT4A	mannosyl (alpha-1,3-)-glycoprotein beta-1,4-N-acetylglucosaminyltransferase, isozyme A
11070	TMEM115	transmembrane protein 115
463	ZFHX3	zinc finger homeobox 3
10307	APBB3	amyloid beta (A4) precursor protein-binding, family B, member 3
9577	BRE	brain and reproductive organ-expressed (TNFRSF1A modulator)
116985	ARAP1	ArfGAP with RhoGAP domain, ankyrin repeat and PH domain 1
1201	CLN3	ceroid-lipofuscinosis, neuronal 3
5140	PDE3B	phosphodiesterase 3B, cGMP-inhibited
23646	PLD3	phospholipase D family, member 3
54625	PARP14	poly (ADP-ribose) polymerase family, member 14
55841	WWC3	WWC family member 3
126003	TRAPPC5	trafficking protein particle complex 5
5732	PTGER2	prostaglandin E receptor 2 (subtype EP2), 53kDa
7704	ZBTB16	zinc finger and BTB domain containing 16
5987	TRIM27	tripartite motif containing 27
219855	SLC37A2	solute carrier family 37 (glycerol-3-phosphate transporter), member 2
4277	MICB	MHC class I polypeptide-related sequence B
8795	TNFRSF10B	tumor necrosis factor receptor superfamily, member 10b
81844	TRIM56	tripartite motif containing 56
11018	TMED1	transmembrane emp24 protein transport domain containing 1
6652	SORD	sorbitol dehydrogenase
57533	TBC1D14	TBC1 domain family, member 14
9354	UBE4A	ubiquitination factor E4A (UFD2 homolog, yeast)
51312	SLC25A37	solute carrier family 25, member 37
2720	GLB1	galactosidase, beta 1
64759	TNS3	tensin 3
84034	EMILIN2	elastin microfibril interfacer 2
5351	PLOD1	procollagen-lysine 1, 2-oxoglutarate 5-dioxygenase 1
114769	CARD16	caspase recruitment domain family, member 16
10981	RAB32	RAB32, member RAS oncogene family
10170	DHRS9	dehydrogenase/reductase (SDR family) member 9
9262	STK17B	serine/threonine kinase 17b
9960	USP3	ubiquitin specific peptidase 3
285533	RNF175	ring finger protein 175
3628	INPP1	inositol polyphosphate-1-phosphatase
1979	EIF4EBP2	eukaryotic translation initiation factor 4E binding protein 2
10870	HCST	hematopoietic cell signal transducer
57590	WDFY1	WD repeat and FYVE domain containing 1
9470	EIF4E2	eukaryotic translation initiation factor 4E family member 2
64127	NOD2	nucleotide-binding oligomerization domain containing 2
10866	HCP5	HLA complex P5
1876	E2F6	E2F transcription factor 6
1318	SLC31A2	solute carrier family 31 (copper transporters), member 2
79901	CYBRD1	cytochrome b reductase 1
824	CAPN2	calpain 2, (m/II) large subunit
3459	IFNGR1	interferon gamma receptor 1
5265	SERPINA1	serpin peptidase inhibitor, clade A (alpha-1 antiproteinase, antitrypsin), member 1
22918	CD93	CD93 molecule
55022	PID1	phosphotyrosine interaction domain containing 1
11213	IRAK3	interleukin-1 receptor-associated kinase 3
10288	LILRB2	leukocyte immunoglobulin-like receptor, subfamily B (with TM and ITIM domains), member 2
23608	MKRN1	makorin ring finger protein 1
96459	FNIP1	folliculin interacting protein 1
2634	GBP2	guanylate binding protein 2, interferon-inducible
9446	GSTO1	glutathione S-transferase omega 1
4653	MYOC	myocilin, trabecular meshwork inducible glucocorticoid response
25977	NECAP1	NECAP endocytosis associated 1
642	BLMH	bleomycin hydrolase
837	CASP4	caspase 4, apoptosis-related cysteine peptidase
2733	GLE1	GLE1 RNA export mediator homolog (yeast)
2799	GNS	glucosamine (N-acetyl)-6-sulfatase
6252	RTN1	reticulon 1
7045	TGFBI	transforming growth factor, beta-induced, 68kDa
85450	ITPRIP	inositol 1,4,5-triphosphate receptor interacting protein
1050	CEBPA	CCAAT/enhancer binding protein (C/EBP), alpha
27287	VENTX	VENT homeobox homolog (Xenopus laevis)
11025	LILRB3	leukocyte immunoglobulin-like receptor, subfamily B (with TM and ITIM domains), member 3
9046	DOK2	docking protein 2, 56kDa
8495	PPFIBP2	PTPRF interacting protein, binding protein 2 (liprin beta 2)
5862	RAB2A	RAB2A, member RAS oncogene family
54861	SNRK	SNF related kinase
6992	PPP1R11	protein phosphatase 1, regulatory (inhibitor) subunit 11

1389	CREBL2	cAMP responsive element binding protein-like 2
4682	NUBP1	nucleotide binding protein 1 (MinD homolog, E. coli)
7263	TST	thiosulfate sulfurtransferase (rhodanese)
3916	LAMP1	lysosomal-associated membrane protein 1
7248	TSC1	tuberous sclerosis 1
968	CD68	CD68 molecule
1051	CEBPB	CCAAT/enhancer binding protein (C/EBP), beta
375387	LRRC33	leucine rich repeat containing 33
54103	PION	pigeon homolog (Drosophila)
3098	HK1	hexokinase 1
5899	RALB	v-ral simian leukemia viral oncogene homolog B (ras related; GTP binding protein)
23208	SYT11	synaptotagmin XI
1436	CSF1R	colony stimulating factor 1 receptor
834	CASP1	caspase 1, apoptosis-related cysteine peptidase (interleukin 1, beta, convertase)
11033	ADAP1	ArfGAP with dual PH domains 1
51296	SLC15A3	solute carrier family 15, member 3
5210	PFKFB4	6-phosphofructo-2-kinase/fructose-2,6-biphosphatase 4

CD14+ blood monocytes (downregulated genes)

ENTREZ ID	Gene Symbol	Gene Name
10090	UST	uronyl-2-sulfotransferase
3111	HLA-DOA	major histocompatibility complex, class II, DO alpha
388962	BOLA3	bolA homolog 3 (E. coli)
7430	EZR	ezrin
8099	CDK2AP1	cyclin-dependent kinase 2 associated protein 1
5800	PTPRO	protein tyrosine phosphatase, receptor type, O
6047	RNF4	ring finger protein 4
55509	BATF3	basic leucine zipper transcription factor, ATF-like 3
84319	C3orf26	chromosome 3 open reading frame 26
10263	CDK2AP2	cyclin-dependent kinase 2 associated protein 2
4171	MCM2	minichromosome maintenance complex component 2
140707	BRI3BP	BRI3 binding protein
1163	CKS1B	CDC28 protein kinase regulatory subunit 1B
80221	ACSF2	acyl-CoA synthetase family member 2
4673	NAP1L1	nucleosome assembly protein 1-like 1
55619	DOCK10	dedicator of cytokinesis 10
6647	SOD1	superoxide dismutase 1, soluble
3092	HIP1	huntingtin interacting protein 1
9978	RBX1	ring-box 1, E3 ubiquitin protein ligase
23231	SEL1L3	sel-1 suppressor of lin-12-like 3 (C. elegans)
81608	FIP1L1	FIP1 like 1 (S. cerevisiae)
286367	LOC286367	FP944
55737	VPS35	vacuolar protein sorting 35 homolog (S. cerevisiae)
338707	B4GALNT4	beta-1,4-N-acetyl-galactosaminyl transferase 4
113246	C12orf57	chromosome 12 open reading frame 57
3615	IMPDH2	IMP (inosine 5'-monophosphate) dehydrogenase 2
2322	FLT3	fms-related tyrosine kinase 3
5250	SLC25A3	solute carrier family 25 (mitochondrial carrier; phosphate carrier), member 3
27244	SESN1	sestrin 1
10576	CCT2	chaperonin containing TCP1, subunit 2 (beta)
2665	GDI2	GDP dissociation inhibitor 2
404770	FAM75B	family with sequence similarity 75, member B
23234	DNAJC9	DnaJ (Hsp40) homolog, subfamily C, member 9
10172	ZNF256	zinc finger protein 256
56061	UBFD1	ubiquitin family domain containing 1
10606	PAICS	phosphoribosylaminoimidazole carboxylase, phosphoribosylaminoimidazole succinocarboxamide synthetase
51005	AMDHD2	amidohydrolase domain containing 2
55748	CNDP2	CNDP dipeptidase 2 (metallopeptidase M20 family)
90313	TP53I13	tumor protein p53 inducible protein 13
339221	ENPP7	ectonucleotide pyrophosphatase/phosphodiesterase 7
6209	RPS15	ribosomal protein S15
1503	CTPS	CTP synthase
2972	BRF1	BRF1 homolog, subunit of RNA polymerase III transcription initiation factor IIIB (S. cerevisiae)
4953	ODC1	ornithine decarboxylase 1
2287	FKBP3	FK506 binding protein 3, 25kDa
11340	EXOSC8	exosome component 8
162979	ZNF296	zinc finger protein 296
3275	PRMT2	protein arginine methyltransferase 2
401397	LOC401397	hypothetical LOC401397
27346	TMEM97	transmembrane protein 97
653162	RPSAP9	ribosomal protein SA pseudogene 9
8237	USP11	ubiquitin specific peptidase 11
60343	FAM3A	family with sequence similarity 3, member A
56271	BEX4	brain expressed, X-linked 4
1743	DLST	dihydrolipoamide S-succinyltransferase (E2 component of 2-oxo-glutarate complex)
10019	SH2B3	SH2B adaptor protein 3
90594	ZNF439	zinc finger protein 439
274	BIN1	bridging integrator 1
8445	DYRK2	dual-specificity tyrosine-(Y)-phosphorylation regulated kinase 2
2119	ETV5	ets variant 5
5709	PSMD3	proteasome (prosome, macropain) 26S subunit, non-ATPase, 3
2064	ERBB2	v-erb-b2 erythroblastic leukemia viral oncogene homolog 2, neuro/glioblastoma derived oncogene homolog (avian)
1021	CDK6	cyclin-dependent kinase 6
1019	CDK4	cyclin-dependent kinase 4
10068	IL18BP	interleukin 18 binding protein
10093	ARPC4	actin related protein 2/3 complex, subunit 4, 20kDa
5134	PDCD2	programmed cell death 2
23157	SEPT6	septin 6
57599	WDR48	WD repeat domain 48
6233	RPS27A	ribosomal protein S27a
55521	TRIM36	tripartite motif containing 36
57823	SLAMF7	SLAM family member 7
10096	ACTR3	ARP3 actin-related protein 3 homolog (yeast)
25849	PARM1	prostate androgen-regulated mucin-like protein 1
440	ASNS	asparagine synthetase (glutamine-hydrolyzing)

2592	GALT	galactose-1-phosphate uridylyltransferase
10051	SMC4	structural maintenance of chromosomes 4
6261	RYR1	ryanodine receptor 1 (skeletal)
166614	DCLK2	doublecortin-like kinase 2
2317	FLNB	filamin B, beta
6193	RPS5	ribosomal protein S5
8991	SELENBP1	selenium binding protein 1
79140	CCDC28B	coiled-coil domain containing 28B
7371	UCK2	uridine-cytidine kinase 2
8934	RAB7L1	RAB7, member RAS oncogene family-like 1
729786	GOLGA8C	golgin A8 family, member C
9185	REPS2	RALBP1 associated Eps domain containing 2
51703	ACSL5	acyl-CoA synthetase long-chain family member 5
649946	RPL23AP64	ribosomal protein L23a pseudogene 64
85453	TSPYL5	TSPY-like 5
57600	FNIP2	folliculin interacting protein 2
23677	SH3BP4	SH3-domain binding protein 4
770	CA11	carbonic anhydrase XI
5176	SERPINF1	serpin peptidase inhibitor, clade F (alpha-2 antiplasmin, pigment epithelium derived factor), member 1
8575	PRKRA	protein kinase, interferon-inducible double stranded RNA dependent activator
6132	RPL8	ribosomal protein L8
729739	LOC729739	hypothetical LOC729739
8841	HDAC3	histone deacetylase 3
27106	ARRDC2	arrestin domain containing 2
4839	NOP2	NOP2 nucleolar protein homolog (yeast)
54843	SYTL2	synaptotagmin-like 2
64766	S100PBP	S100P binding protein
60492	CCDC90B	coiled-coil domain containing 90B
3638	INSIG1	insulin induced gene 1
23165	NUP205	nucleoporin 205kDa
10947	AP3M2	adaptor-related protein complex 3, mu 2 subunit
6920	TCEA3	transcription elongation factor A (SII), 3
51477	ISYNA1	inositol-3-phosphate synthase 1
3921	RPSA	ribosomal protein SA
5993	RFX5	regulatory factor X, 5 (influences HLA class II expression)
81539	SLC38A1	solute carrier family 38, member 1
118433	RPL23AP7	ribosomal protein L23a pseudogene 7
6235	RPS29	ribosomal protein S29
134	ADORA1	adenosine A1 receptor
54908	CCDC99	coiled-coil domain containing 99
4723	NDUFV1	NADH dehydrogenase (ubiquinone) flavoprotein 1, 51kDa
253769	WDR27	WD repeat domain 27
55214	LEPREL1	leprecan-like 1
493861	EID3	EP300 interacting inhibitor of differentiation 3
79829	NAA40	N(alpha)-acetyltransferase 40, NatD catalytic subunit, homolog (S. cerevisiae)
51602	NOP58	NOP58 ribonucleoprotein homolog (yeast)
1595	CYP51A1	cytochrome P450, family 51, subfamily A, polypeptide 1
5319	PLA2G1B	phospholipase A2, group IB (pancreas)
11332	ACOT7	acyl-CoA thioesterase 7
55915	LANCL2	LanC lantibiotic synthetase component C-like 2 (bacterial)
201134	CCDC46	coiled-coil domain containing 46
60489	APOBEC3G	apolipoprotein B mRNA editing enzyme, catalytic polypeptide-like 3G
11224	RPL35	ribosomal protein L35
6158	RPL28	ribosomal protein L28
56478	EIF4ENIF1	eukaryotic translation initiation factor 4E nuclear import factor 1
10755	GIPC1	GIPC PDZ domain containing family, member 1
1933	EEF1B2	eukaryotic translation elongation factor 1 beta 2
7405	UVRAG	UV radiation resistance associated gene
8706	B3GALNT1	beta-1,3-N-acetylgalactosaminyltransferase 1 (globoside blood group)
2995	GYPC	glycophorin C (Gerbich blood group)
10589	DRAP1	DR1-associated protein 1 (negative cofactor 2 alpha)
4818	NKG7	natural killer cell group 7 sequence
7283	TUBG1	tubulin, gamma 1
2675	GFRA2	GDNF family receptor alpha 2
23098	SARM1	sterile alpha and TIR motif containing 1
6136	RPL12	ribosomal protein L12
57475	PLEKHH1	pleckstrin homology domain containing, family H (with MyTH4 domain) member 1
85015	USP45	ubiquitin specific peptidase 45
11279	KLF8	Kruppel-like factor 8
129293	C2orf89	chromosome 2 open reading frame 89
6232	RPS27	ribosomal protein S27
23555	TSPAN15	tetraspanin 15
51692	CPSF3	cleavage and polyadenylation specific factor 3, 73kDa
9497	SLC4A7	solute carrier family 4, sodium bicarbonate cotransporter, member 7
54806	AHI1	Abelson helper integration site 1
388574	FLJ43681	ribosomal protein L23a pseudogene
6335	SCN9A	sodium channel, voltage-gated, type IX, alpha subunit
9249	DHRS3	dehydrogenase/reductase (SDR family) member 3
54733	SLC35F2	solute carrier family 35, member F2
26003	GORASP2	golgi reassembly stacking protein 2, 55kDa
677838	SNORA61	small nucleolar RNA, H/ACA box 61
121268	RHEBL1	Ras homolog enriched in brain like 1
5424	POLD1	polymerase (DNA directed), delta 1, catalytic subunit 125kDa
3925	STMN1	stathmin 1
898	CCNE1	cyclin E1
644511	RPL13AP6	ribosomal protein L13a pseudogene 6
57026	PDXP	pyridoxal (pyridoxine, vitamin B6) phosphatase
4145	MATK	megakaryocyte-associated tyrosine kinase
220930	LOC220930	hypothetical LOC220930
79864	C11orf63	chromosome 11 open reading frame 63
6904	TBCD	tubulin folding cofactor D
26228	STAP1	signal transducing adaptor family member 1
4175	MCM6	minichromosome maintenance complex component 6
971	CD72	CD72 molecule
283491	OR7E156P	olfactory receptor, family 7, subfamily E, member 156 pseudogene

10743	RAI1	retinoic acid induced 1
4047	LSS	lanosterol synthase (2,3-oxidosqualene-lanosterol cyclase)
79697	C14orf169	chromosome 14 open reading frame 169
4642	MYO1D	myosin ID
8622	PDE8B	phosphodiesterase 8B
8481	OFD1	oral-facial-digital syndrome 1
60	ACTB	actin, beta
55526	DHTKD1	dehydrogenase E1 and transketolase domain containing 1
6717	SRI	sorcin
2788	GNG7	guanine nucleotide binding protein (G protein), gamma 7
476	ATP1A1	ATPase, Na+/K+ transporting, alpha 1 polypeptide
1235	CCR6	chemokine (C-C motif) receptor 6
79705	LRRK1	leucine-rich repeat kinase 1
57493	HEG1	HEG homolog 1 (zebrafish)
6389	SDHA	succinate dehydrogenase complex, subunit A, flavoprotein (Fp)
6484	ST3GAL4	ST3 beta-galactoside alpha-2,3-sialyltransferase 4
79856	SNX22	sorting nexin 22
23264	ZC3H7B	zinc finger CCCH-type containing 7B
221188	GPR114	G protein-coupled receptor 114
801	CALM1	calmodulin 1 (phosphorylase kinase, delta)
63910	SLC17A9	solute carrier family 17, member 9
29919	C18orf8	chromosome 18 open reading frame 8
23046	KIF21B	kinesin family member 21B
84617	TUBB6	tubulin, beta 6
5475	PPEF1	protein phosphatase, EF-hand calcium binding domain 1
81571	NCRNA00287	non-protein coding RNA 287
28969	BZW2	basic leucine zipper and W2 domains 2
25873	RPL36	ribosomal protein L36
25994	HIGD1A	HIG1 hypoxia inducible domain family, member 1A
57121	LPAR5	lysophosphatidic acid receptor 5
1633	DCK	deoxycytidine kinase
57037	ANKMY2	ankyrin repeat and MYND domain containing 2
51559	NT5DC3	5'-nucleotidase domain containing 3
149018	LELP1	late cornified envelope-like proline-rich 1
55624	POMGNT1	protein O-linked mannose beta1,2-N-acetylglucosaminyltransferase
3006	HIST1H1C	histone cluster 1, H1c
4065	LY75	lymphocyte antigen 75
80127	C14orf45	chromosome 14 open reading frame 45
23390	ZDHHC17	zinc finger, DHHC-type containing 17
1803	DPP4	dipeptidyl-peptidase 4
5335	PLCG1	phospholipase C, gamma 1
6122	RPL3	ribosomal protein L3
150946	FAM59B	family with sequence similarity 59, member B
158056	MAMDC4	MAM domain containing 4
79085	SLC25A23	solute carrier family 25 (mitochondrial carrier; phosphate carrier), member 23
51655	RASD1	RAS, dexamethasone-induced 1
64798	DEPTOR	DEP domain containing MTOR-interacting protein
343477	HSP90B3P	heat shock protein 90kDa beta (Grp94), member 3 (pseudogene)
27143	KIAA1274	KIAA1274
29760	BLNK	B-cell linker
4239	MFAP4	microfibrillar-associated protein 4
8644	AKR1C3	aldo-keto reductase family 1, member C3 (3-alpha hydroxysteroid dehydrogenase, type II)
653125	LOC653125	Golgin subfamily A member 8-like protein 1-like
152559	PAQR3	progestin and adipoQ receptor family member III
5478	PPIA	peptidylprolyl isomerase A (cyclophilin A)
25904	CNOT10	CCR4-NOT transcription complex, subunit 10
23649	POLA2	polymerase (DNA directed), alpha 2 (70kD subunit)
3338	DNAJC4	DnaJ (Hsp40) homolog, subfamily C, member 4
285268	ZNF621	zinc finger protein 621
55266	TMEM19	transmembrane protein 19
51678	MPP6	membrane protein, palmitoylated 6 (MAGUK p55 subfamily member 6)
165055	CCDC138	coiled-coil domain containing 138
5333	PLCD1	phospholipase C, delta 1
10505	SEMA4F	sema domain, immunoglobulin domain (Ig), transmembrane domain (TM) and short cytoplasmic domain, (semaphorin) 4F
10627	MYL12A	myosin, light chain 12A, regulatory, non-sarcomeric
55897	MESP1	mesoderm posterior 1 homolog (mouse)
51339	DACT1	dapper, antagonist of beta-catenin, homolog 1 (Xenopus laevis)
728743	LOC728743	similar to GLI-Kruppel family member HKR1
80115	BAIAP2L2	BAI1-associated protein 2-like 2
81606	LBH	limb bud and heart development homolog (mouse)
92922	CCDC102A	coiled-coil domain containing 102A
115290	FBXO17	F-box protein 17
9214	FAIM3	Fas apoptotic inhibitory molecule 3
7976	FZD3	frizzled homolog 3 (Drosophila)
55353	LAPTM4B	lysosomal protein transmembrane 4 beta
7915	ALDH5A1	aldehyde dehydrogenase 5 family, member A1
57576	KIF17	kinesin family member 17
145508	C14orf145	chromosome 14 open reading frame 145
3815	KIT	v-kit Hardy-Zuckerman 4 feline sarcoma viral oncogene homolog
387882	C12orf75	chromosome 12 open reading frame 75
51474	LIMA1	LIM domain and actin binding 1
54549	SDK2	sidekick homolog 2 (chicken)
712	C1QA	complement component 1, q subcomponent, A chain
10666	CD226	CD226 molecule
399665	FAM102A	family with sequence similarity 102, member A
51268	PIPOX	pipecolic acid oxidase
9806	SPOCK2	sparc/osteonectin, cwcv and kazal-like domains proteoglycan (testican) 2
253827	MSRB3	methionine sulfoxide reductase B3
587	BCAT2	branched chain amino-acid transaminase 2, mitochondrial
157769	FAM91A1	family with sequence similarity 91, member A1
84433	CARD11	caspase recruitment domain family, member 11
53834	FGFRL1	fibroblast growth factor receptor-like 1
1601	DAB2	disabled homolog 2, mitogen-responsive phosphoprotein (Drosophila)
90060	CCDC120	coiled-coil domain containing 120
55964	SEPT3	septin 3

4041	LRP5	low density lipoprotein receptor-related protein 5
5159	PDGFRB	platelet-derived growth factor receptor, beta polypeptide
79172	CENPO	centromere protein O
3386	ICAM4	intercellular adhesion molecule 4 (Landsteiner-Wiener blood group)
2781	GNAZ	guanine nucleotide binding protein (G protein), alpha z polypeptide
55342	STRBP	spermatid perinuclear RNA binding protein
1906	EDN1	endothelin 1
55083	KIF26B	kinesin family member 26B
151888	BTLA	B and T lymphocyte associated
999	CDH1	cadherin 1, type 1, E-cadherin (epithelial)
3775	KCNK1	potassium channel, subfamily K, member 1
344148	NCKAP5	NCK-associated protein 5
6242	RTKN	rhotekin
114899	C1QTNF3	C1q and tumor necrosis factor related protein 3
921	CD5	CD5 molecule
5754	PTK7	PTK7 protein tyrosine kinase 7
5168	ENPP2	ectonucleotide pyrophosphatase/phosphodiesterase 2
4099	MAG	myelin associated glycoprotein
28978	TMEM14A	transmembrane protein 14A
2273	FHL1	four and a half LIM domains 1
5624	PROC	protein C (inactivator of coagulation factors Va and VIIIa)
55615	PRR5	proline rich 5 (renal)
7098	TLR3	toll-like receptor 3
2199	FBLN2	fibulin 2
121053	C12orf45	chromosome 12 open reading frame 45
8506	CNTNAP1	contactin associated protein 1
5151	PDE8A	phosphodiesterase 8A
2100	ESR2	estrogen receptor 2 (ER beta)
2833	CXCR3	chemokine (C-X-C motif) receptor 3
27090	ST6GALNAC4	ST6 (alpha-N-acetyl-neuraminyl-2,3-beta-galactosyl-1,3)-N-acetylgalactosaminide alpha-2,6-sialyltransferase 4
257144	GCET2	germinal center expressed transcript 2
50649	ARHGEF4	Rho guanine nucleotide exchange factor (GEF) 4
3800	KIF5C	kinesin family member 5C
9957	HS3ST1	heparan sulfate (glucosamine) 3-O-sulfotransferase 1
494513	DFNB59	deafness, autosomal recessive 59
9892	SNAP91	synaptosomal-associated protein, 91kDa homolog (mouse)
84707	BEX2	brain expressed X-linked 2
4629	MYH11	myosin, heavy chain 11, smooth muscle
400508	NCRNA00169	non-protein coding RNA 169
85358	SHANK3	SH3 and multiple ankyrin repeat domains 3
57211	GPR126	G protein-coupled receptor 126
10253	SPRY2	sprouty homolog 2 (Drosophila)
3002	GZMB	granzyme B (granzyme 2, cytotoxic T-lymphocyte-associated serine esterase 1)
55691	FRMD4A	FERM domain containing 4A
2621	GAS6	growth arrest-specific 6
55384	MEG3	maternally expressed 3 (non-protein coding)
81552	VOPP1	vesicular, overexpressed in cancer, prosurvival protein 1
5519	PPP2R1B	protein phosphatase 2, regulatory subunit A, beta
946	SIGLEC6	sialic acid binding Ig-like lectin 6
282618	IL29	interleukin 29 (interferon, lambda 1)
3902	LAG3	lymphocyte-activation gene 3
440712	C1orf186	chromosome 1 open reading frame 186
6608	SMO	smoothened homolog (Drosophila)
116028	C16orf75	chromosome 16 open reading frame 75
83988	NCALD	neurocalcin delta
51760	SYT17	synaptotagmin XVII
100505501	LOC100505501	hypothetical LOC100505501
219736	STOX1	storkhead box 1
7169	TPM2	tropomyosin 2 (beta)
445328	ARHGEF35	Rho guanine nucleotide exchange factor (GEF) 35
991	CDC20	cell division cycle 20 homolog (S. cerevisiae)
116238	TLCD1	TLC domain containing 1
7710	ZNF154	zinc finger protein 154
326342	EMR4P	egf-like module containing, mucin-like, hormone receptor-like 4 pseudogene
126669	SHE	Src homology 2 domain containing E
7984	ARHGEF5	Rho guanine nucleotide exchange factor (GEF) 5
728715	LOC728715	ovostatin homolog 2-like
11098	PRSS23	protease, serine, 23
5539	PPY	pancreatic polypeptide
57657	HCN3	hyperpolarization activated cyclic nucleotide-gated potassium channel 3
22874	PLEKHA6	pleckstrin homology domain containing, family A member 6
9760	TOX	thymocyte selection-associated high mobility group box
5358	PLS3	plastin 3
638	BIK	BCL2-interacting killer (apoptosis-inducing)
84660	CCDC62	coiled-coil domain containing 62
55653	BCAS4	breast carcinoma amplified sequence 4
23242	COBL	cordon-bleu homolog (mouse)
55086	CXorf57	chromosome X open reading frame 57
79656	BEND5	BEN domain containing 5
1815	DRD4	dopamine receptor D4
57447	NDRG2	NDRG family member 2
9963	SLC23A1	solute carrier family 23 (nucleobase transporters), member 1
100505650	LOC100505650	hypothetical LOC100505650
219902	TMEM136	transmembrane protein 136
79007	DBNDD1	dysbindin (dystrobrevin binding protein 1) domain containing 1
3741	KCNA5	potassium voltage-gated channel, shaker-related subfamily, member 5
152485	ZNF827	zinc finger protein 827
91319	DERL3	Der1-like domain family, member 3
54836	BSPRY	B-box and SPRY domain containing
1000	CDH2	cadherin 2, type 1, N-cadherin (neuronal)
389816	LRRC26	leucine rich repeat containing 26
5129	CDK18	cyclin-dependent kinase 18
3852	KRT5	keratin 5
116159	CYYR1	cysteine/tyrosine-rich 1
434	ASIP	agouti signaling protein

113451	ADC	arginine decarboxylase
51237	MZB1	marginal zone B and B1 cell-specific protein
3512	IGJ	immunoglobulin J polypeptide, linker protein for immunoglobulin alpha and mu polypeptides
5730	PTGDS	prostaglandin D2 synthase 21kDa (brain)
80326	WNT10A	wingless-type MMTV integration site family, member 10A
23189	KANK1	KN motif and ankyrin repeat domains 1
54106	TLR9	toll-like receptor 9
163702	IL28RA	interleukin 28 receptor, alpha (interferon, lambda receptor)
2047	EPHB1	EPH receptor B1
153769	SH3RF2	SH3 domain containing ring finger 2

CD16+ monocytes (upregulated genes)

ENTREZ ID	Gene Symbol	Gene Name
719	C3AR1	complement component 3a receptor 1
6556	SLC11A1	solute carrier family 11 (proton-coupled divalent metal ion transporters), member 1
943	TNFRSF8	tumor necrosis factor receptor superfamily, member 8
58475	MS4A7	membrane-spanning 4-domains, subfamily A, member 7
838	CASP5	caspase 5, apoptosis-related cysteine peptidase
113791	PIK3IP1	phosphoinositide-3-kinase interacting protein 1
5326	PLAGL2	pleiomorphic adenoma gene-like 2
9935	MAFB	v-maf musculoaponeurotic fibrosarcoma oncogene homolog B (avian)
3800	KIF5C	kinesin family member 5C
3386	ICAM4	intercellular adhesion molecule 4 (Landsteiner-Wiener blood group)
8714	ABCC3	ATP-binding cassette, sub-family C (CFTR/MRP), member 3
83541	FAM110A	family with sequence similarity 110, member A
2592	GALT	galactose-1-phosphate uridylyltransferase
83931	STK40	serine/threonine kinase 40
5908	RAP1B	RAP1B, member of RAS oncogene family
51274	KLF3	Kruppel-like factor 3 (basic)
255919	TMEM188	transmembrane protein 188
10924	SMPDL3A	sphingomyelin phosphodiesterase, acid-like 3A
147699	PPM1N	protein phosphatase, Mg2+/Mn2+ dependent, 1N (putative)
8863	PER3	period homolog 3 (Drosophila)
29948	OSGIN1	oxidative stress induced growth inhibitor 1
8932	MBD2	methyl-CpG binding domain protein 2
6237	RRAS	related RAS viral (r-ras) oncogene homolog
51092	SIDT2	SID1 transmembrane family, member 2
83593	RASSF5	Ras association (RalGDS/AF-6) domain family member 5
27106	ARRDC2	arrestin domain containing 2
161742	SPRED1	sprouty-related, EVH1 domain containing 1
5783	PTPN13	protein tyrosine phosphatase, non-receptor type 13 (APO-1/CD95 (Fas)-associated phosphatase)
29097	CNIH4	cornichon homolog 4 (Drosophila)
6840	SVIL	supervillin
23098	SARM1	sterile alpha and TIR motif containing 1
55145	THAP1	THAP domain containing, apoptosis associated protein 1
64411	ARAP3	ArfGAP with RhoGAP domain, ankyrin repeat and PH domain 3
8519	IFITM1	interferon induced transmembrane protein 1 (9-27)
159162	RBMY2FP	RNA binding motif protein, Y-linked, family 2, member F pseudogene
55615	PRR5	proline rich 5 (renal)
116372	LYPD1	LY6/PLAUR domain containing 1
53834	FGFRL1	fibroblast growth factor receptor-like 1
84957	RELT	RELT tumor necrosis factor receptor
10589	DRAP1	DR1-associated protein 1 (negative cofactor 2 alpha)
2192	FBLN1	fibulin 1
22821	RASA3	RAS p21 protein activator 3
51760	SYT17	synaptotagmin XVII
898	CCNE1	cyclin E1
55384	MEG3	maternally expressed 3 (non-protein coding)
57659	ZBTB4	zinc finger and BTB domain containing 4
3422	IDI1	isopentenyl-diphosphate delta isomerase 1
693159	MIR574	microRNA 574
3638	INSIG1	insulin induced gene 1
171586	ABHD3	abhydrolase domain containing 3
29979	UBQLN1	ubiquilin 1
4061	LY6E	lymphocyte antigen 6 complex, locus E
80139	ZNF703	zinc finger protein 703
79412	KREMEN2	kringle containing transmembrane protein 2
100130557	LOC100130557	hypothetical LOC100130557
7408	VASP	vasodilator-stimulated phosphoprotein
55143	CDCA8	cell division cycle associated 8
1488	CTBP2	C-terminal binding protein 2
84689	MS4A14	membrane-spanning 4-domains, subfamily A, member 14
1534	CYB561	cytochrome b-561
5585	PKN1	protein kinase N1
100128059	LOC100128059	hypothetical LOC100128059
57010	CABP4	calcium binding protein 4
4239	MFAP4	microfibrillar-associated protein 4
100132967	NCRNA00204	non-protein coding RNA 204
8520	HAT1	histone acetyltransferase 1
55723	ASF1B	ASF1 anti-silencing function 1 homolog B (S. cerevisiae)
125206	SLC5A10	solute carrier family 5 (sodium/glucose cotransporter), member 10
643418	LIPN	lipase, family member N
57182	ANKRD50	ankyrin repeat domain 50
6674	SPAG1	sperm associated antigen 1
5064	PALM	paralemmin
9213	XPR1	xenotropic and polytropic retrovirus receptor 1
23552	CDK20	cyclin-dependent kinase 20
7320	UBE2B	ubiquitin-conjugating enzyme E2B (RAD6 homolog)
57493	HEG1	HEG homolog 1 (zebrafish)
25771	TBC1D22A	TBC1 domain family, member 22A
4067	LYN	v-yes-1 Yamaguchi sarcoma viral related oncogene homolog
57804	POLD4	polymerase (DNA-directed), delta 4
7764	ZNF217	zinc finger protein 217
22808	MRAS	muscle RAS oncogene homolog
6904	TBCD	tubulin folding cofactor D

3688	ITGB1	integrin, beta 1 (fibronectin receptor, beta polypeptide, antigen CD29 includes MDF2, MSK12)
29919	C18orf8	chromosome 18 open reading frame 8
388	RHOB	ras homolog gene family, member B
55788	LMBRD1	LMBR1 domain containing 1
7367	UGT2B17	UDP glucuronosyltransferase 2 family, polypeptide B17
3759	KCNJ2	potassium inwardly-rectifying channel, subfamily J, member 2
79605	PGBD5	piggyBac transposable element derived 5
5606	MAP2K3	mitogen-activated protein kinase kinase 3
404636	FAM45A	family with sequence similarity 45, member A
50651	SLC45A1	solute carrier family 45, member 1
6813	STXBP2	syntaxin binding protein 2
116985	ARAP1	ArfGAP with RhoGAP domain, ankyrin repeat and PH domain 1
7099	TLR4	toll-like receptor 4
23508	TTC9	tetratricopeptide repeat domain 9
23046	KIF21B	kinesin family member 21B
9821	RB1CC1	RB1-inducible coiled-coil 1
3707	ITPKB	inositol 1,4,5-trisphosphate 3-kinase B
2001	ELF5	E74-like factor 5 (ets domain transcription factor)
10916	MAGED2	melanoma antigen family D, 2
84926	SPRYD3	SPRY domain containing 3
6253	RTN2	reticulon 2
162989	DEDD2	death effector domain containing 2
4772	NFATC1	nuclear factor of activated T-cells, cytoplasmic, calcineurin-dependent 1
90362	FAM110B	family with sequence similarity 110, member B
6576	SLC25A1	solute carrier family 25 (mitochondrial carrier; citrate transporter), member 1
221981	THSD7A	thrombospondin, type I, domain containing 7A
4651	MYO10	myosin X
197257	LDHD	lactate dehydrogenase D
100131607	LOC100131607	hypothetical LOC100131607
79065	ATG9A	ATG9 autophagy related 9 homolog A (S. cerevisiae)
51527	C14orf129	chromosome 14 open reading frame 129
23338	PHF15	PHD finger protein 15
80194	TMEM134	transmembrane protein 134
100507321	LOC100507321	hypothetical LOC100507321
408	ARRB1	arrestin, beta 1
51155	HN1	hematological and neurological expressed 1
2983	GUCY1B3	guanylate cyclase 1, soluble, beta 3
246330	PELI3	pellino homolog 3 (Drosophila)
157	ADRBK2	adrenergic, beta, receptor kinase 2
246175	CNOT6L	CCR4-NOT transcription complex, subunit 6-like
1906	EDN1	endothelin 1
27202	GPR77	G protein-coupled receptor 77
57826	RAP2C	RAP2C, member of RAS oncogene family
10581	IFITM2	interferon induced transmembrane protein 2 (1-8D)
717	C2	complement component 2
4673	NAP1L1	nucleosome assembly protein 1-like 1
1163	CKS1B	CDC28 protein kinase regulatory subunit 1B
6647	SOD1	superoxide dismutase 1, soluble
353514	LILRA5	leukocyte immunoglobulin-like receptor, subfamily A (with TM domain), member 5
93323	HAUS8	HAUS augmin-like complex, subunit 8
9537	TP53I11	tumor protein p53 inducible protein 11
151556	GPR155	G protein-coupled receptor 155
51280	GOLM1	golgi membrane protein 1
54453	RIN2	Ras and Rab interactor 2
585	BBS4	Bardet-Biedl syndrome 4
22919	MAPRE1	microtubule-associated protein, RP/EB family, member 1
833	CARS	cysteinyl-tRNA synthetase
2633	GBP1	guanylate binding protein 1, interferon-inducible
8622	PDE8B	phosphodiesterase 8B
9975	NR1D2	nuclear receptor subfamily 1, group D, member 2
56181	FAM54B	family with sequence similarity 54, member B
201627	FAM116A	family with sequence similarity 116, member A
6102	RP2	retinitis pigmentosa 2 (X-linked recessive)
4883	NPR3	natriuretic peptide receptor C/guanylate cyclase C (atrionatriuretic peptide receptor C)
28966	SNX24	sorting nexin 24
83693	HSDL1	hydroxysteroid dehydrogenase like 1
3455	IFNAR2	interferon (alpha, beta and omega) receptor 2
10461	MERTK	c-mer proto-oncogene tyrosine kinase
79154	DHRS11	dehydrogenase/reductase (SDR family) member 11
1486	CTBS	chitobiase, di-N-acetyl-
388182	FLJ42289	hypothetical LOC388182
527	ATP6V0C	ATPase, H+ transporting, lysosomal 16kDa, V0 subunit c
8915	BCL10	B-cell CLL/lymphoma 10
11182	SLC2A6	solute carrier family 2 (facilitated glucose transporter), member 6
54622	ARL15	ADP-ribosylation factor-like 15
56894	AGPAT3	1-acylglycerol-3-phosphate O-acyltransferase 3
79148	MMP28	matrix metallopeptidase 28
53	ACP2	acid phosphatase 2, lysosomal
29992	PILRA	paired immunoglobin-like type 2 receptor alpha
51311	TLR8	toll-like receptor 8
6398	SECTM1	secreted and transmembrane 1
79852	EPHX3	epoxide hydrolase 3
7752	ZNF200	zinc finger protein 200
4744	NEFH	neurofilament, heavy polypeptide
55055	ZWILCH	Zwilch, kinetochore associated, homolog (Drosophila)
23328	SASH1	SAM and SH3 domain containing 1
54458	PRR13	proline rich 13
5165	PDK3	pyruvate dehydrogenase kinase, isozyme 3
10154	PLXNC1	plexin C1
6655	SOS2	son of sevenless homolog 2 (Drosophila)
55344	PLCXD1	phosphatidylinositol-specific phospholipase C, X domain containing 1
57192	MCOLN1	mucolipin 1
10890	RAB10	RAB10, member RAS oncogene family
149951	COMMD7	COMM domain containing 7
2869	GRK5	G protein-coupled receptor kinase 5

23469	PHF3	PHD finger protein 3
26136	TES	testis derived transcript (3 LIM domains)
10312	TCIRG1	T-cell, immune regulator 1, ATPase, H+ transporting, lysosomal V0 subunit A3
90007	MIDN	midnolin
7464	CORO2A	coronin, actin binding protein, 2A
60682	SMAP1	small ArfGAP 1
5159	PDGFRB	platelet-derived growth factor receptor, beta polypeptide
11156	PTP4A3	protein tyrosine phosphatase type IVA, member 3
11275	KLHL2	kelch-like 2, Mayven (Drosophila)
3176	HNMT	histamine N-methyltransferase
51765	MST4	serine/threonine protein kinase MST4
8445	DYRK2	dual-specificity tyrosine-(Y)-phosphorylation regulated kinase 2
284422	C19orf77	chromosome 19 open reading frame 77
60492	CCDC90B	coiled-coil domain containing 90B
54464	XRN1	5'-3' exoribonuclease 1
23416	KCNH3	potassium voltage-gated channel, subfamily H (eag-related), member 3
56890	MDM1	Mdm1 nuclear protein homolog (mouse)
26118	WSB1	WD repeat and SOCS box containing 1
64072	CDH23	cadherin-related 23
23315	SLC9A8	solute carrier family 9 (sodium/hydrogen exchanger), member 8
27244	SESN1	sestrin 1
10435	CDC42EP2	CDC42 effector protein (Rho GTPase binding) 2
1050	CEBPA	CCAAT/enhancer binding protein (C/EBP), alpha
23585	TMEM50A	transmembrane protein 50A
5222	PGA5	pepsinogen 5, group I (pepsinogen A)
832	CAPZB	capping protein (actin filament) muscle Z-line, beta
83464	APH1B	anterior pharynx defective 1 homolog B (C. elegans)
81606	LBH	limb bud and heart development homolog (mouse)
6794	STK11	serine/threonine kinase 11
441455	LOC441455	makorin ring finger protein 1 pseudogene
60489	APOBEC3G	apolipoprotein B mRNA editing enzyme, catalytic polypeptide-like 3G
4117	MAK	male germ cell-associated kinase
4043	LRPAP1	low density lipoprotein receptor-related protein associated protein 1
3489	IGFBP6	insulin-like growth factor binding protein 6
25903	OLFML2B	olfactomedin-like 2B
4122	MAN2A2	mannosidase, alpha, class 2A, member 2
5732	PTGER2	prostaglandin E receptor 2 (subtype EP2), 53kDa
4012	LNPEP	leucyl/cystinyl aminopeptidase
282974	STK32C	serine/threonine kinase 32C
158358	KIAA2026	KIAA2026
55181	C17orf71	chromosome 17 open reading frame 71
80896	NPL	N-acetylneuraminate pyruvate lyase (dihydrodipicolinate synthase)
91351	DDX60L	DEAD (Asp-Glu-Ala-Asp) box polypeptide 60-like
3978	LIG1	ligase I, DNA, ATP-dependent
54965	PIGX	phosphatidylinositol glycan anchor biosynthesis, class X
88455	ANKRD13A	ankyrin repeat domain 13A
84668	FAM126A	family with sequence similarity 126, member A
9474	ATG5	ATG5 autophagy related 5 homolog (S. cerevisiae)
112616	CMTM7	CKLF-like MARVEL transmembrane domain containing 7
718	C3	complement component 3
60684	C4orf41	chromosome 4 open reading frame 41
51111	SUV420H1	suppressor of variegation 4-20 homolog 1 (Drosophila)
57498	KIDINS220	kinase D-interacting substrate, 220kDa
387	RHOA	ras homolog gene family, member A
6453	ITSN1	intersectin 1 (SH3 domain protein)
84173	ELMOD3	ELMO/CED-12 domain containing 3
27128	CYTH4	cytohesin 4
65018	PINK1	PTEN induced putative kinase 1
10288	LILRB2	leukocyte immunoglobulin-like receptor, subfamily B (with TM and ITIM domains), member 2
3305	HSPA1L	heat shock 70kDa protein 1-like
9780	FAM38A	family with sequence similarity 38, member A
8705	B3GALT4	UDP-Gal:betaGlcNAc beta 1,3-galactosyltransferase, polypeptide 4
79137	FAM134A	family with sequence similarity 134, member A
8477	GPR65	G protein-coupled receptor 65
54438	GFOD1	glucose-fructose oxidoreductase domain containing 1
79720	VPS37B	vacuolar protein sorting 37 homolog B (S. cerevisiae)
5265	SERPINA1	serpin peptidase inhibitor, clade A (alpha-1 antiproteinase, antitrypsin), member 1
200030	NBPF11	neuroblastoma breakpoint family, member 11
9119	KRT75	keratin 75
23167	EFR3A	EFR3 homolog A (S. cerevisiae)
283219	KCTD21	potassium channel tetramerisation domain containing 21
54507	ADAMTSL4	ADAMTS-like 4
1387	CREBBP	CREB binding protein
2550	GABBR1	gamma-aminobutyric acid (GABA) B receptor, 1
5800	PTPRO	protein tyrosine phosphatase, receptor type, O
490	ATP2B1	ATPase, Ca++ transporting, plasma membrane 1
1155	TBCB	tubulin folding cofactor B
11168	PSIP1	PC4 and SFRS1 interacting protein 1
3660	IRF2	interferon regulatory factor 2
712	C1QA	complement component 1, q subcomponent, A chain
11138	TBC1D8	TBC1 domain family, member 8 (with GRAM domain)
7739	ZNF185	zinc finger protein 185 (LIM domain)
619434	NCRNA00051	non-protein coding RNA 51
974	CD79B	CD79b molecule, immunoglobulin-associated beta
79020	C7orf25	chromosome 7 open reading frame 25
9262	STK17B	serine/threonine kinase 17b
139596	UPRT	uracil phosphoribosyltransferase (FUR1) homolog (S. cerevisiae)
1318	SLC31A2	solute carrier family 31 (copper transporters), member 2
25979	DHRS7B	dehydrogenase/reductase (SDR family) member 7B
80267	EDEM3	ER degradation enhancer, mannosidase alpha-like 3
203054	ADCK5	aarF domain containing kinase 5
10908	PNPLA6	patatin-like phospholipase domain containing 6
80256	KIAA1539	KIAA1539
994	CDC25B	cell division cycle 25 homolog B (S. pombe)
348180	CTU2	cytosolic thiouridylase subunit 2 homolog (S. pombe)

6778	STAT6	signal transducer and activator of transcription 6, interleukin-4 induced
79168	LILRA6	leukocyte immunoglobulin-like receptor, subfamily A (with TM domain), member 6
5912	RAP2B	RAP2B, member of RAS oncogene family
11213	IRAK3	interleukin-1 receptor-associated kinase 3
56895	AGPAT4	1-acylglycerol-3-phosphate O-acyltransferase 4 (lysophosphatidic acid acyltransferase, delta)
2771	GNAI2	guanine nucleotide binding protein (G protein), alpha inhibiting activity polypeptide 2
55763	EXOC1	exocyst complex component 1
25977	NECAP1	NECAP endocytosis associated 1
219855	SLC37A2	solute carrier family 37 (glycerol-3-phosphate transporter), member 2
1436	CSF1R	colony stimulating factor 1 receptor
2634	GBP2	guanylate binding protein 2, interferon-inducible
81844	TRIM56	tripartite motif containing 56
138151	NACC2	NACC family member 2, BEN and BTB (POZ) domain containing
25798	BRI3	brain protein I3
6051	RNPEP	arginyl aminopeptidase (aminopeptidase B)
23654	PLXNB2	plexin B2
8869	ST3GAL5	ST3 beta-galactoside alpha-2,3-sialyltransferase 5
5987	TRIM27	tripartite motif containing 27
6397	SEC14L1	SEC14-like 1 (S. cerevisiae)
134510	UBLCP1	ubiquitin-like domain containing CTD phosphatase 1
2885	GRB2	growth factor receptor-bound protein 2
55654	TMEM127	transmembrane protein 127
10410	IFITM3	interferon induced transmembrane protein 3 (1-8U)
7170	TPM3	tropomyosin 3
474338	SUMO1P3	SUMO1 pseudogene 3
643180	CCT6P3	chaperonin containing TCP1, subunit 6 (zeta) pseudogene 3
339448	C1orf174	chromosome 1 open reading frame 174
79132	DHX58	DEXH (Asp-Glu-X-His) box polypeptide 58
8462	KLF11	Kruppel-like factor 11
7060	THBS4	thrombospondin 4
4259	MGST3	microsomal glutathione S-transferase 3
5140	PDE3B	phosphodiesterase 3B, cGMP-inhibited
7732	RNF112	ring finger protein 112
55357	TBC1D2	TBC1 domain family, member 2
55008	HERC6	hect domain and RLD 6
80301	PLEKHO2	pleckstrin homology domain containing, family O member 2
23608	MKRN1	makorin ring finger protein 1
9046	DOK2	docking protein 2, 56kDa
3588	IL10RB	interleukin 10 receptor, beta
9826	ARHGEF11	Rho guanine nucleotide exchange factor (GEF) 11
10092	ARPC5	actin related protein 2/3 complex, subunit 5, 16kDa
4354	MPP1	membrane protein, palmitoylated 1, 55kDa
8650	NUMB	numb homolog (Drosophila)
4277	MICB	MHC class I polypeptide-related sequence B
27287	VENTX	VENT homeobox homolog (Xenopus laevis)
80727	TTYH3	tweety homolog 3 (Drosophila)
114769	CARD16	caspase recruitment domain family, member 16
54861	SNRK	SNF related kinase
57827	C6orf47	chromosome 6 open reading frame 47
100129550	LOC100129550	hypothetical LOC100129550
51592	TRIM33	tripartite motif containing 33
64114	TMBIM1	transmembrane BAX inhibitor motif containing 1
1138	CHRNA5	cholinergic receptor, nicotinic, alpha 5
115548	FCHO2	FCH domain only 2
55858	TMEM165	transmembrane protein 165
808	CALM3	calmodulin 3 (phosphorylase kinase, delta)
80025	PANK2	pantothenate kinase 2
196383	RILPL2	Rab interacting lysosomal protein-like 2
23303	KIF13B	kinesin family member 13B
2526	FUT4	fucosyltransferase 4 (alpha (1,3) fucosyltransferase, myeloid-specific)
5899	RALB	v-ral simian leukemia viral oncogene homolog B (ras related; GTP binding protein)
9470	EIF4E2	eukaryotic translation initiation factor 4E family member 2
5701	PSMC2	proteasome (prosome, macropain) 26S subunit, ATPase, 2
55454	CSGALNACT2	chondroitin sulfate N-acetylgalactosaminyltransferase 2
22863	ATG14	ATG14 autophagy related 14 homolog (S. cerevisiae)
79901	CYBRD1	cytochrome b reductase 1
2733	GLE1	GLE1 RNA export mediator homolog (yeast)
2799	GNS	glucosamine (N-acetyl)-6-sulfatase
51621	KLF13	Kruppel-like factor 13
9997	SCO2	SCO cytochrome oxidase deficient homolog 2 (yeast)
121457	IKBIP	IKBKB interacting protein
2665	GDI2	GDP dissociation inhibitor 2
476	ATP1A1	ATPase, Na+/K+ transporting, alpha 1 polypeptide
637	BID	BH3 interacting domain death agonist
352961	HCG26	HLA complex group 26 (non-protein coding)
54834	GDAP2	ganglioside induced differentiation associated protein 2
57590	WDFY1	WD repeat and FYVE domain containing 1
6992	PPP1R11	protein phosphatase 1, regulatory (inhibitor) subunit 11
146923	RUNDC1	RUN domain containing 1
1051	CEBPB	CCAAT/enhancer binding protein (C/EBP), beta
114826	SMYD4	SET and MYND domain containing 4
220929	ZNF438	zinc finger protein 438
55521	TRIM36	tripartite motif containing 36
341405	ANKRD33	ankyrin repeat domain 33
64422	ATG3	ATG3 autophagy related 3 homolog (S. cerevisiae)
4666	NACA	nascent polypeptide-associated complex alpha subunit
100287171	WASH1	WAS protein family homolog 1
84293	C10orf58	chromosome 10 open reading frame 58
6195	RPS6KA1	ribosomal protein S6 kinase, 90kDa, polypeptide 1
10970	CKAP4	cytoskeleton-associated protein 4
27348	TOR1B	torsin family 1, member B (torsin B)
5210	PFKFB4	6-phosphofructo-2-kinase/fructose-2,6-biphosphatase 4
83666	PARP9	poly (ADP-ribose) polymerase family, member 9
9061	PAPSS1	3'-phosphoadenosine 5'-phosphosulfate synthase 1
1102	RCBTB2	regulator of chromosome condensation (RCC1) and BTB (POZ) domain containing protein 2

55803	ADAP2	ArfGAP with dual PH domains 2
3098	HK1	hexokinase 1
968	CD68	CD68 molecule
256586	LYSMD2	LysM, putative peptidoglycan-binding, domain containing 2
10422	UBAC1	UBA domain containing 1
8564	KMO	kynurenine 3-monooxygenase (kynurenine 3-hydroxylase)
8621	CDK13	cyclin-dependent kinase 13
10096	ACTR3	ARP3 actin-related protein 3 homolog (yeast)
834	CASP1	caspase 1, apoptosis-related cysteine peptidase (interleukin 1, beta, convertase)
3987	LIMS1	LIM and senescent cell antigen-like domains 1
6277	S100A6	S100 calcium binding protein A6
9839	ZEB2	zinc finger E-box binding homeobox 2
23240	KIAA0922	KIAA0922
57532	NUFIP2	nuclear fragile X mental retardation protein interacting protein 2
1979	EIF4EBP2	eukaryotic translation initiation factor 4E binding protein 2
5351	PLOD1	procollagen-lysine 1, 2-oxoglutarate 5-dioxygenase 1
23208	SYT11	synaptotagmin XI
11033	ADAP1	ArfGAP with dual PH domains 1
837	CASP4	caspase 4, apoptosis-related cysteine peptidase
3916	LAMP1	lysosomal-associated membrane protein 1
9960	USP3	ubiquitin specific peptidase 3
11025	LILRB3	leukocyte immunoglobulin-like receptor, subfamily B (with TM and ITIM domains), member 3
7450	VWF	von Willebrand factor
126669	SHE	Src homology 2 domain containing E
116238	TLCD1	TLC domain containing 1

CD16+ monocytes (downregulated genes)

ENTREZ ID	Gene Symbol	Gene Name
2322	FLT3	fms-related tyrosine kinase 3
23151	GRAMD4	GRAM domain containing 4
2876	GPX1	glutathione peroxidase 1
9744	ACAP1	ArfGAP with coiled-coil, ankyrin repeat and PH domains 1
109	ADCY3	adenylate cyclase 3
23677	SH3BP4	SH3-domain binding protein 4
11270	NRM	nurim (nuclear envelope membrane protein)
4257	MGST1	microsomal glutathione S-transferase 1
3111	HLA-DOA	major histocompatibility complex, class II, DO alpha
29803	REPIN1	replication initiator 1
113675	SDSL	serine dehydratase-like
5445	PON2	paraoxonase 2
85012	TCEAL3	transcription elongation factor A (SII)-like 3
642	BLMH	bleomycin hydrolase
64784	CRTC3	CREB regulated transcription coactivator 3
3108	HLA-DMA	major histocompatibility complex, class II, DM alpha
125144	NCRNA00188	non-protein coding RNA 188
140467	ZNF358	zinc finger protein 358
2535	FZD2	frizzled homolog 2 (Drosophila)
80212	CCDC92	coiled-coil domain containing 92
29903	CCDC106	coiled-coil domain containing 106
1798	DPAGT1	dolichyl-phosphate (UDP-N-acetylglucosamine) N-acetylglucosaminephosphotransferase 1 (GlcNAc-1-P transferase)
64218	SEMA4A	sema domain, immunoglobulin domain(Ig), transmembrane domain(TM) and short cytoplasmic domain, (semaphorin) 4A
6209	RPS15	ribosomal protein S15
51060	TXNDC12	thioredoxin domain containing 12 (endoplasmic reticulum)
157285	SGK223	homolog of rat pragma of Rnd2
81932	HDHD3	haloacid dehalogenase-like hydrolase domain containing 3
6133	RPL9	ribosomal protein L9
5863	RGL2	ral guanine nucleotide dissociation stimulator-like 2
5993	RFX5	regulatory factor X, 5 (influences HLA class II expression)
7371	UCK2	uridine-cytidine kinase 2
51133	KCTD3	potassium channel tetramerisation domain containing 3
3092	HIP1	huntingtin interacting protein 1
4752	NEK3	NIMA (never in mitosis gene a)-related kinase 3
2788	GNG7	guanine nucleotide binding protein (G protein), gamma 7
6234	RPS28	ribosomal protein S28
90843	TCEAL8	transcription elongation factor A (SII)-like 8
51754	TMEM8B	transmembrane protein 8B
2531	KDSR	3-ketodihydrosphingosine reductase
80305	TRABD	TraB domain containing
2120	ETV6	ets variant 6
7570	ZNF22	zinc finger protein 22 (KOX 15)
6443	SGCB	sarcoglycan, beta (43kDa dystrophin-associated glycoprotein)
4171	MCM2	minichromosome maintenance complex component 2
9804	TOMM20	translocase of outer mitochondrial membrane 20 homolog (yeast)
6176	RPLP1	ribosomal protein, large, P1
286102	TMED10P1	transmembrane emp24-like trafficking protein 10 (yeast) pseudogene 1
84879	MFSD2A	major facilitator superfamily domain containing 2A
8407	TAGLN2	transgelin 2
51314	TXNDC3	thioredoxin domain containing 3 (spermatozoa)
2926	GRSF1	G-rich RNA sequence binding factor 1
8943	AP3D1	adaptor-related protein complex 3, delta 1 subunit
1200	TPP1	tripeptidyl peptidase I
6396	SEC13	SEC13 homolog (S. cerevisiae)
54849	DEF8	differentially expressed in FDCP 8 homolog (mouse)
3957	LGALS2	lectin, galactoside-binding, soluble, 2
130589	GALM	galactose mutarotase (aldose 1-epimerase)
157567	ANKRD46	ankyrin repeat domain 46
5296	PIK3R2	phosphoinositide-3-kinase, regulatory subunit 2 (beta)
80145	THOC7	THO complex 7 homolog (Drosophila)
6202	RPS8	ribosomal protein S8
283345	RPL13P5	ribosomal protein L13 pseudogene 5
3490	IGFBP7	insulin-like growth factor binding protein 7
6175	RPLP0	ribosomal protein, large, P0
9577	BRE	brain and reproductive organ-expressed (TNFRSF1A modulator)
8192	CLPP	ClpP caseinolytic peptidase, ATP-dependent, proteolytic subunit homolog (E. coli)
57533	TBC1D14	TBC1 domain family, member 14

30968	STOML2	stomatin (EPB72)-like 2
8575	PRKRA	protein kinase, interferon-inducible double stranded RNA dependent activator
7594	ZNF43	zinc finger protein 43
9466	IL27RA	interleukin 27 receptor, alpha
283102	FLJ46111	keratin 8 pseudogene
6168	RPL37A	ribosomal protein L37a
6205	RPS11	ribosomal protein S11
6626	SNRPA	small nuclear ribonucleoprotein polypeptide A
256355	RPS2P32	ribosomal protein S2 pseudogene 32
11070	TMEM115	transmembrane protein 115
2289	FKBP5	FK506 binding protein 5
29927	SEC61A1	Sec61 alpha 1 subunit (S. cerevisiae)
5176	SERPINF1	serpin peptidase inhibitor, clade F (alpha-2 antiplasmin, pigment epithelium derived factor), member 1
63875	MRPL17	mitochondrial ribosomal protein L17
79004	CUEDC2	CUE domain containing 2
51107	APH1A	anterior pharynx defective 1 homolog A (C. elegans)
26354	GNL3	guanine nucleotide binding protein-like 3 (nucleolar)
3074	HEXB	hexosaminidase B (beta polypeptide)
9117	SEC22C	SEC22 vesicle trafficking protein homolog C (S. cerevisiae)
4999	ORC2	origin recognition complex, subunit 2
1201	CLN3	ceroid-lipofuscinosis, neuronal 3
8669	EIF3J	eukaryotic translation initiation factor 3, subunit J
4723	NDUFV1	NADH dehydrogenase (ubiquinone) flavoprotein 1, 51kDa
51056	LAP3	leucine aminopeptidase 3
5747	PTK2	PTK2 protein tyrosine kinase 2
58505	OSTC	oligosaccharyltransferase complex subunit
3615	IMPDH2	IMP (inosine 5'-monophosphate) dehydrogenase 2
8991	SELENBP1	selenium binding protein 1
6892	TAPBP	TAP binding protein (tapasin)
51011	FAHD2A	fumarylacetoacetate hydrolase domain containing 2A
23231	SEL1L3	sel-1 suppressor of lin-12-like 3 (C. elegans)
90313	TP53I13	tumor protein p53 inducible protein 13
274	BIN1	bridging integrator 1
29844	TFPT	TCF3 (E2A) fusion partner (in childhood Leukemia)
9044	BTAF1	BTAF1 RNA polymerase II, B-TFIID transcription factor-associated, 170kDa (Mot1 homolog, S. cerevisiae)
148789	B3GALNT2	beta-1,3-N-acetylgalactosaminyltransferase 2
7263	TST	thiosulfate sulfurtransferase (rhodanese)
57212	KIAA0495	KIAA0495
7982	ST7	suppression of tumorigenicity 7
6161	RPL32	ribosomal protein L32
85440	DOCK7	dedicator of cytokinesis 7
689	BTF3	basic transcription factor 3
6143	RPL19	ribosomal protein L19
909	CD1A	CD1a molecule
151313	FAHD2B	fumarylacetoacetate hydrolase domain containing 2B
54985	HCFC1R1	host cell factor C1 regulator 1 (XPO1 dependent)
57026	PDXP	pyridoxal (pyridoxine, vitamin B6) phosphatase
653162	RPSAP9	ribosomal protein SA pseudogene 9
29994	BAZ2B	bromodomain adjacent to zinc finger domain, 2B
1632	DCI	dodecenoyl-CoA isomerase
4245	MGAT1	mannosyl (alpha-1,3-)-glycoprotein beta-1,2-N-acetylglucosaminyltransferase
6194	RPS6	ribosomal protein S6
1139	CHRNA7	cholinergic receptor, nicotinic, alpha 7
10959	TMED2	transmembrane emp24 domain trafficking protein 2
126755	LRRC38	leucine rich repeat containing 38
6158	RPL28	ribosomal protein L28
57176	VARS2	valyl-tRNA synthetase 2, mitochondrial (putative)
9552	SPAG7	sperm associated antigen 7
860	RUNX2	runt-related transcription factor 2
8975	USP13	ubiquitin specific peptidase 13 (isopeptidase T-3)
6125	RPL5	ribosomal protein L5
163732	CITED4	Cbp/p300-interacting transactivator, with Glu/Asp-rich carboxy-terminal domain, 4
79174	CRELD2	cysteine-rich with EGF-like domains 2
3682	ITGAE	integrin, alpha E (antigen CD103, human mucosal lymphocyte antigen 1; alpha polypeptide)
26996	GPR160	G protein-coupled receptor 160
27346	TMEM97	transmembrane protein 97
11188	NISCH	nischarin
285148	IAH1	isoamyl acetate-hydrolyzing esterase 1 homolog (S. cerevisiae)
199990	C1orf86	chromosome 1 open reading frame 86
402	ARL2	ADP-ribosylation factor-like 2
100289019	LOC100289019	hypothetical LOC100289019
5074	PAWR	PRKC, apoptosis, WT1, regulator
6160	RPL31	ribosomal protein L31
56623	INPP5E	inositol polyphosphate-5-phosphatase, 72 kDa
645683	RPL13AP3	ribosomal protein L13a pseudogene 3
493	ATP2B4	ATPase, Ca++ transporting, plasma membrane 4
359948	IRF2BP2	interferon regulatory factor 2 binding protein 2
6137	RPL13	ribosomal protein L13
255758	TCTEX1D2	Tctex1 domain containing 2
93663	ARHGAP18	Rho GTPase activating protein 18
6189	RPS3A	ribosomal protein S3A
6138	RPL15	ribosomal protein L15
22807	IKZF2	IKAROS family zinc finger 2 (Helios)
6187	RPS2	ribosomal protein S2
8766	RAB11A	RAB11A, member RAS oncogene family
10205	MPZL2	myelin protein zero-like 2
56922	MCCC1	methylcrotonoyl-CoA carboxylase 1 (alpha)
641638	SNHG6	small nucleolar RNA host gene 6 (non-protein coding)
26985	AP3M1	adaptor-related protein complex 3, mu 1 subunit
56271	BEX4	brain expressed, X-linked 4
6181	RPLP2	ribosomal protein, large, P2
9501	RPH3AL	rabphilin 3A-like (without C2 domains)
55911	APOBR	apolipoprotein B receptor
55959	SULF2	sulfatase 2
3551	IKBKB	inhibitor of kappa light polypeptide gene enhancer in B-cells, kinase beta

5425	POLD2	polymerase (DNA directed), delta 2, regulatory subunit 50kDa
10174	SORBS3	sorbin and SH3 domain containing 3
56924	PAK6	p21 protein (Cdc42/Rac)-activated kinase 6
3156	HMGCR	3-hydroxy-3-methylglutaryl-CoA reductase
118433	RPL23AP7	ribosomal protein L23a pseudogene 7
6207	RPS13	ribosomal protein S13
387066	SNHG5	small nucleolar RNA host gene 5 (non-protein coding)
23158	TBC1D9	TBC1 domain family, member 9 (with GRAM domain)
644511	RPL13AP6	ribosomal protein L13a pseudogene 6
9637	FEZ2	fasciculation and elongation protein zeta 2 (zygin II)
401321	LOC401321	hypothetical LOC401321
51727	CMPK1	cytidine monophosphate (UMP-CMP) kinase 1, cytosolic
7405	UVRAG	UV radiation resistance associated gene
6228	RPS23	ribosomal protein S23
79140	CCDC28B	coiled-coil domain containing 28B
54733	SLC35F2	solute carrier family 35, member F2
2529	FUT7	fucosyltransferase 7 (alpha (1,3) fucosyltransferase)
7307	U2AF1	U2 small nuclear RNA auxiliary factor 1
79921	TCEAL4	transcription elongation factor A (SII)-like 4
9631	NUP155	nucleoporin 155kDa
8425	LTBP4	latent transforming growth factor beta binding protein 4
80765	STARD5	StAR-related lipid transfer (START) domain containing 5
6128	RPL6	ribosomal protein L6
51559	NT5DC3	5'-nucleotidase domain containing 3
9692	KIAA0391	KIAA0391
8455	ATRN	attractin
9889	ZBED4	zinc finger, BED-type containing 4
971	CD72	CD72 molecule
1595	CYP51A1	cytochrome P450, family 51, subfamily A, polypeptide 1
6728	SRP19	signal recognition particle 19kDa
10576	CCT2	chaperonin containing TCP1, subunit 2 (beta)
203069	R3HCC1	R3H domain and coiled-coil containing 1
85453	TSPYL5	TSPY-like 5
6193	RPS5	ribosomal protein S5
2091	FBL	fibrillarin
113179	ADAT3	adenosine deaminase, tRNA-specific 3, TAD3 homolog (S. cerevisiae)
9855	FARP2	FERM, RhoGEF and pleckstrin domain protein 2
10455	PECI	peroxisomal D3,D2-enoyl-CoA isomerase
6201	RPS7	ribosomal protein S7
440	ASNS	asparagine synthetase (glutamine-hydrolyzing)
64764	CREB3L2	cAMP responsive element binding protein 3-like 2
23002	DAAM1	dishevelled associated activator of morphogenesis 1
129293	C2orf89	chromosome 2 open reading frame 89
6155	RPL27	ribosomal protein L27
8665	EIF3F	eukaryotic translation initiation factor 3, subunit F
286053	NSMCE2	non-SMC element 2, MMS21 homolog (S. cerevisiae)
587	BCAT2	branched chain amino-acid transaminase 2, mitochondrial
6622	SNCA	synuclein, alpha (non A4 component of amyloid precursor)
1066	CES1	carboxylesterase 1
79746	ECHDC3	enoyl CoA hydratase domain containing 3
729786	GOLGA8C	golgin A8 family, member C
23394	ADNP	activity-dependent neuroprotector homeobox
25845	LOC25845	hypothetical LOC25845
84953	MICALCL	MICAL C-terminal like
388564	LOC388564	hypothetical protein LOC388564
79713	TMEM149	transmembrane protein 149
646567	LOC646567	oligosaccharyltransferase complex subunit OSTC-like
55816	DOK5	docking protein 5
10360	NPM3	nucleophosmin/nucleoplasmin 3
64759	TNS3	tensin 3
84451	KIAA1804	mixed lineage kinase 4
10632	ATP5L	ATP synthase, H+ transporting, mitochondrial Fo complex, subunit G
8841	HDAC3	histone deacetylase 3
911	CD1C	CD1c molecule
79630	C1orf54	chromosome 1 open reading frame 54
10113	PREB	prolactin regulatory element binding
27130	INVS	inversin
9588	PRDX6	peroxiredoxin 6
253769	WDR27	WD repeat domain 27
64221	ROBO3	roundabout, axon guidance receptor, homolog 3 (Drosophila)
29100	TMEM208	transmembrane protein 208
64073	C19orf33	chromosome 19 open reading frame 33
4736	RPL10A	ribosomal protein L10a
4649	MYO9A	myosin IXA
79710	MORC4	MORC family CW-type zinc finger 4
84080	C16orf48	chromosome 16 open reading frame 48
9727	RAB11FIP3	RAB11 family interacting protein 3 (class II)
6141	RPL18	ribosomal protein L18
1933	EEF1B2	eukaryotic translation elongation factor 1 beta 2
84717	HDGFRP2	hepatoma-derived growth factor-related protein 2
4839	NOP2	NOP2 nucleolar protein homolog (yeast)
649946	RPL23AP64	ribosomal protein L23a pseudogene 64
134	ADORA1	adenosine A1 receptor
131474	CHCHD4	coiled-coil-helix-coiled-coil-helix domain containing 4
54454	ATAD2B	ATPase family, AAA domain containing 2B
1431	CS	citrate synthase
349196	LOC349196	hypothetical LOC349196
11164	NUDT5	nudix (nucleoside diphosphate linked moiety X)-type motif 5
7554	ZNF8	zinc finger protein 8
6721	SREBF2	sterol regulatory element binding transcription factor 2
6335	SCN9A	sodium channel, voltage-gated, type IX, alpha subunit
6188	RPS3	ribosomal protein S3
79469	DLEU2L	deleted in lymphocytic leukemia 2-like
64080	RBKS	ribokinase
10213	PSMD14	proteasome (prosome, macropain) 26S subunit, non-ATPase, 14

7226	TRPM2	transient receptor potential cation channel, subfamily M, member 2
55214	LEPREL1	leprecan-like 1
501	ALDH7A1	aldehyde dehydrogenase 7 family, member A1
81539	SLC38A1	solute carrier family 38, member 1
10330	CNPY2	canopy 2 homolog (zebrafish)
6152	RPL24	ribosomal protein L24
10106	CTDSP2	CTD (carboxy-terminal domain, RNA polymerase II, polypeptide A) small phosphatase 2
5150	PDE7A	phosphodiesterase 7A
2644	GCHFR	GTP cyclohydrolase I feedback regulator
23646	PLD3	phospholipase D family, member 3
5546	PRCC	papillary renal cell carcinoma (translocation-associated)
10462	CLEC10A	C-type lectin domain family 10, member A
51455	REV1	REV1 homolog (S. cerevisiae)
92610	TIFA	TRAF-interacting protein with forkhead-associated domain
25849	PARM1	prostate androgen-regulated mucin-like protein 1
85015	USP45	ubiquitin specific peptidase 45
6157	RPL27A	ribosomal protein L27a
10681	GNB5	guanine nucleotide binding protein (G protein), beta 5
8706	B3GALNT1	beta-1,3-N-acetylgalactosaminyltransferase 1 (globoside blood group)
9296	ATP6V1F	ATPase, H+ transporting, lysosomal 14kDa, V1 subunit F
23521	RPL13A	ribosomal protein L13a
55790	CSGALNACT1	chondroitin sulfate N-acetylgalactosaminyltransferase 1
55526	DHTKD1	dehydrogenase E1 and transketolase domain containing 1
3693	ITGB5	integrin, beta 5
100131211	TMEM194B	transmembrane protein 194B
1616	DAXX	death-domain associated protein
348110	C15orf38	chromosome 15 open reading frame 38
388574	FLJ43681	ribosomal protein L23a pseudogene
23234	DNAJC9	DnaJ (Hsp40) homolog, subfamily C, member 9
54869	EPS8L1	EPS8-like 1
10863	ADAM28	ADAM metallopeptidase domain 28
23474	ETHE1	ethylmalonic encephalopathy 1
2184	FAH	fumarylacetoacetate hydrolase (fumarylacetoacetase)
6136	RPL12	ribosomal protein L12
54806	AHI1	Abelson helper integration site 1
23193	GANAB	glucosidase, alpha; neutral AB
440567	UQCRHL	ubiquinol-cytochrome c reductase hinge protein-like
9404	LPXN	leupaxin
55227	LRRC1	leucine rich repeat containing 1
343477	HSP90B3P	heat shock protein 90kDa beta (Grp94), member 3 (pseudogene)
78999	LRFN4	leucine rich repeat and fibronectin type III domain containing 4
9725	TMEM63A	transmembrane protein 63A
6732	SRPK1	SRSF protein kinase 1
155370	SBDSP1	Shwachman-Bodian-Diamond syndrome pseudogene 1
145508	C14orf145	chromosome 14 open reading frame 145
6191	RPS4X	ribosomal protein S4, X-linked
64854	USP46	ubiquitin specific peptidase 46
3703	STT3A	STT3, subunit of the oligosaccharyltransferase complex, homolog A (S. cerevisiae)
9197	SLC33A1	solute carrier family 33 (acetyl-CoA transporter), member 1
10947	AP3M2	adaptor-related protein complex 3, mu 2 subunit
5738	PTGFRN	prostaglandin F2 receptor negative regulator
51477	ISYNA1	inositol-3-phosphate synthase 1
56926	NCLN	nicalin
2205	FCER1A	Fc fragment of IgE, high affinity I, receptor for; alpha polypeptide
26031	OSBPL3	oxysterol binding protein-like 3
29121	CLEC2D	C-type lectin domain family 2, member D
84617	TUBB6	tubulin, beta 6
1394	CRHR1	corticotropin releasing hormone receptor 1
1235	CCR6	chemokine (C-C motif) receptor 6
80347	COASY	CoA synthase
9051	PSTPIP1	proline-serine-threonine phosphatase interacting protein 1
11006	LILRB4	leukocyte immunoglobulin-like receptor, subfamily B (with TM and ITIM domains), member 4
3921	RPSA	ribosomal protein SA
11279	KLF8	Kruppel-like factor 8
6233	RPS27A	ribosomal protein S27a
5364	PLXNB1	plexin B1
6235	RPS29	ribosomal protein S29
84447	SYVN1	synovial apoptosis inhibitor 1, synoviolin
1793	DOCK1	dedicator of cytokinesis 1
8481	OFD1	oral-facial-digital syndrome 1
252969	NEIL2	nei endonuclease VIII-like 2 (E. coli)
150946	FAM59B	family with sequence similarity 59, member B
2194	FASN	fatty acid synthase
60481	ELOVL5	ELOVL family member 5, elongation of long chain fatty acids (FEN1/Elo2, SUR4/Elo3-like, yeast)
22872	SEC31A	SEC31 homolog A (S. cerevisiae)
283248	RCOR2	REST corepressor 2
84705	GTPBP3	GTP binding protein 3 (mitochondrial)
29956	LASS2	LAG1 homolog, ceramide synthase 2
286367	LOC286367	FP944
84572	GNPTG	N-acetylglucosamine-1-phosphate transferase, gamma subunit
6540	SLC6A13	solute carrier family 6 (neurotransmitter transporter, GABA), member 13
729739	LOC729739	hypothetical LOC729739
23333	DPY19L1	dpy-19-like 1 (C. elegans)
55813	UTP6	UTP6, small subunit (SSU) processome component, homolog (yeast)
343413	FCRL6	Fc receptor-like 6
1803	DPP4	dipeptidyl-peptidase 4
196996	GRAMD2	GRAM domain containing 2
1601	DAB2	disabled homolog 2, mitogen-responsive phosphoprotein (Drosophila)
374882	TMEM205	transmembrane protein 205
57576	KIF17	kinesin family member 17
10755	GIPC1	GIPC PDZ domain containing family, member 1
80867	HCG2P7	HLA complex group 2 pseudogene 7
4358	MPV17	MpV17 mitochondrial inner membrane protein
257364	SNX33	sorting nexin 33
144100	PLEKHA7	pleckstrin homology domain containing, family A member 7

1994	ELAVL1	ELAV (embryonic lethal, abnormal vision, Drosophila)-like 1 (Hu antigen R)
7528	YY1	YY1 transcription factor
4638	MYLK	myosin light chain kinase
10606	PAICS	phosphoribosylaminoimidazole carboxylase, phosphoribosylaminoimidazole succinocarboxamide synthetase
23649	POLA2	polymerase (DNA directed), alpha 2 (70kD subunit)
151648	SGOL1	shugoshin-like 1 (S. pombe)
55353	LAPTM4B	lysosomal protein transmembrane 4 beta
166929	SGMS2	sphingomyelin synthase 2
54893	MTMR10	myotubularin related protein 10
6146	RPL22	ribosomal protein L22
56954	NIT2	nitrilase family, member 2
27018	NGFRAP1	nerve growth factor receptor (TNFRSF16) associated protein 1
3159	HMGA1	high mobility group AT-hook 1
6920	TCEA3	transcription elongation factor A (SII), 3
26249	KLHL3	kelch-like 3 (Drosophila)
27143	KIAA1274	KIAA1274
26003	GORASP2	golgi reassembly stacking protein 2, 55kDa
317781	DDX51	DEAD (Asp-Glu-Ala-Asp) box polypeptide 51
440563	LOC440563	heterogeneous nuclear ribonucleoprotein C-like
55756	INTS9	integrator complex subunit 9
142678	MIB2	mindbomb homolog 2 (Drosophila)
57475	PLEKHH1	pleckstrin homology domain containing, family H (with MyTH4 domain) member 1
55614	KIF16B	kinesin family member 16B
6165	RPL35A	ribosomal protein L35a
11320	MGAT4A	mannosyl (alpha-1,3-)-glycoprotein beta-1,4-N-acetylglucosaminyltransferase, isozyme A
81544	GDPD5	glycerophosphodiester phosphodiesterase domain containing 5
131566	DCBLD2	discoidin, CUB and LCCL domain containing 2
9319	TRIP13	thyroid hormone receptor interactor 13
3925	STMN1	stathmin 1
1718	DHCR24	24-dehydrocholesterol reductase
50854	C6orf48	chromosome 6 open reading frame 48
51222	ZNF219	zinc finger protein 219
65985	AACS	acetoacetyl-CoA synthetase
2162	F13A1	coagulation factor XIII, A1 polypeptide
80223	RAB11FIP1	RAB11 family interacting protein 1 (class I)
4642	MYO1D	myosin ID
4283	CXCL9	chemokine (C-X-C motif) ligand 9
53947	A4GALT	alpha 1,4-galactosyltransferase
9651	PLCH2	phospholipase C, eta 2
3338	DNAJC4	DnaJ (Hsp40) homolog, subfamily C, member 4
653125	LOC653125	Golgin subfamily A member 8-like protein 1-like
51134	CCDC41	coiled-coil domain containing 41
26227	PHGDH	phosphoglycerate dehydrogenase
116842	LEAP2	liver expressed antimicrobial peptide 2
2859	GPR35	G protein-coupled receptor 35
6122	RPL3	ribosomal protein L3
80183	C13orf18	chromosome 13 open reading frame 18
9901	SRGAP3	SLIT-ROBO Rho GTPase activating protein 3
28969	BZW2	basic leucine zipper and W2 domains 2
9962	SLC23A2	solute carrier family 23 (nucleobase transporters), member 2
53826	FXYD6	FXYD domain containing ion transport regulator 6
9249	DHRS3	dehydrogenase/reductase (SDR family) member 3
84707	BEX2	brain expressed X-linked 2
9601	PDIA4	protein disulfide isomerase family A, member 4
221188	GPR114	G protein-coupled receptor 114
11339	OIP5	Opa interacting protein 5
55897	MESP1	mesoderm posterior 1 homolog (mouse)
11224	RPL35	ribosomal protein L35
9332	CD163	CD163 molecule
9394	HS6ST1	heparan sulfate 6-O-sulfotransferase 1
29760	BLNK	B-cell linker
9709	HERPUD1	homocysteine-inducible, endoplasmic reticulum stress-inducible, ubiquitin-like domain member 1
910	CD1B	CD1b molecule
7976	FZD3	frizzled homolog 3 (Drosophila)
10009	ZBTB33	zinc finger and BTB domain containing 33
60673	C12orf44	chromosome 12 open reading frame 44
158056	MAMDC4	MAM domain containing 4
2944	GSTM1	glutathione S-transferase mu 1
2948	GSTM4	glutathione S-transferase mu 4
147372	CCBE1	collagen and calcium binding EGF domains 1
79805	VASH2	vasohibin 2
64798	DEPTOR	DEP domain containing MTOR-interacting protein
6484	ST3GAL4	ST3 beta-galactoside alpha-2,3-sialyltransferase 4
6608	SMO	smoothened homolog (Drosophila)
9973	CCS	copper chaperone for superoxide dismutase
160518	DENND5B	DENN/MADD domain containing 5B
57728	WDR19	WD repeat domain 19
7915	ALDH5A1	aldehyde dehydrogenase 5 family, member A1
390539	OR4N3P	olfactory receptor, family 4, subfamily N, member 3 pseudogene
23390	ZDHHC17	zinc finger, DHHC-type containing 17
7292	TNFSF4	tumor necrosis factor (ligand) superfamily, member 4
27240	SIT1	signaling threshold regulating transmembrane adaptor 1
201725	C4orf46	chromosome 4 open reading frame 46
730101	LOC730101	hypothetical LOC730101
121053	C12orf45	chromosome 12 open reading frame 45
3815	KIT	v-kit Hardy-Zuckerman 4 feline sarcoma viral oncogene homolog
55300	PI4K2B	phosphatidylinositol 4-kinase type 2 beta
100506033	LOC100506033	hypothetical LOC100506033
23428	SLC7A8	solute carrier family 7 (amino acid transporter, L-type), member 8
60468	BACH2	BTB and CNC homology 1, basic leucine zipper transcription factor 2
117144	CATSPER1	cation channel, sperm associated 1
169611	OLFML2A	olfactomedin-like 2A
28978	TMEM14A	transmembrane protein 14A
4041	LRP5	low density lipoprotein receptor-related protein 5
221692	PHACTR1	phosphatase and actin regulator 1

5475	PPEF1	protein phosphatase, EF-hand calcium binding domain 1
57447	NDRG2	NDRG family member 2
83787	ARMC10	armadillo repeat containing 10
5184	PEPD	peptidase D
57185	NIPAL3	NIPA-like domain containing 3
25873	RPL36	ribosomal protein L36
7169	TPM2	tropomyosin 2 (beta)
286527	TMSB15B	thymosin beta 15B
148	ADRA1A	adrenergic, alpha-1A-, receptor
10184	LHFPL2	lipoma HMGIC fusion partner-like 2
10579	TACC2	transforming, acidic coiled-coil containing protein 2
913	CD1E	CD1e molecule
399665	FAM102A	family with sequence similarity 102, member A
51205	ACP6	acid phosphatase 6, lysophosphatidic
5478	PPIA	peptidylprolyl isomerase A (cyclophilin A)
9783	RIMS3	regulating synaptic membrane exocytosis 3
2621	GAS6	growth arrest-specific 6
84875	PARP10	poly (ADP-ribose) polymerase family, member 10
728743	LOC728743	similar to GLI-Kruppel family member HKR1
90060	CCDC120	coiled-coil domain containing 120
80115	BAIAP2L2	BAI1-associated protein 2-like 2
4882	NPR2	natriuretic peptide receptor B/guanylate cyclase B (atrionatriuretic peptide receptor B)
152926	PPM1K	protein phosphatase, Mg2+/Mn2+ dependent, 1K
27090	ST6GALNAC4	ST6 (alpha-N-acetyl-neuraminyl-2,3-beta-galactosyl-1,3)-N-acetylgalactosaminide alpha-2,6-sialyltransferase 4
11149	BVES	blood vessel epicardial substance
51474	LIMA1	LIM domain and actin binding 1
999	CDH1	cadherin 1, type 1, E-cadherin (epithelial)
89932	PAPLN	papilin, proteoglycan-like sulfated glycoprotein
8724	SNX3	sorting nexin 3
1969	EPHA2	EPH receptor A2
3009	HIST1H1B	histone cluster 1, H1b
143941	TTC36	tetratricopeptide repeat domain 36
921	CD5	CD5 molecule
2273	FHL1	four and a half LIM domains 1
494513	DFNB59	deafness, autosomal recessive 59
84174	SLA2	Src-like-adaptor 2
23189	KANK1	KN motif and ankyrin repeat domains 1
638	BIK	BCL2-interacting killer (apoptosis-inducing)
81571	NCRNA00287	non-protein coding RNA 287
28951	TRIB2	tribbles homolog 2 (Drosophila)
6242	RTKN	rhotekin
23166	STAB1	stabilin 1
9333	TGM5	transglutaminase 5
158405	KIAA1958	KIAA1958
79801	SHCBP1	SHC SH2-domain binding protein 1
10810	WASF3	WAS protein family, member 3
151888	BTLA	B and T lymphocyte associated
25894	PLEKHG4	pleckstrin homology domain containing, family G (with RhoGef domain) member 4
374650	GOLGA6L5	golgin A6 family-like 5 (pseudogene)
152137	CCDC50	coiled-coil domain containing 50
10253	SPRY2	sprouty homolog 2 (Drosophila)
79656	BEND5	BEN domain containing 5
639	PRDM1	PR domain containing 1, with ZNF domain
641649	TMEM91	transmembrane protein 91
5624	PROC	protein C (inactivator of coagulation factors Va and VIIIa)
80237	ELL3	elongation factor RNA polymerase II-like 3
339390	CLEC4G	C-type lectin domain family 4, member G
55088	C10orf118	chromosome 10 open reading frame 118
25854	FAM149A	family with sequence similarity 149, member A
57211	GPR126	G protein-coupled receptor 126
51268	PIPOX	pipecolic acid oxidase
4925	NUCB2	nucleobindin 2
389634	LOC389634	hypothetical LOC389634
53831	GPR84	G protein-coupled receptor 84
344148	NCKAP5	NCK-associated protein 5
147495	APCDD1	adenomatosis polyposis coli down-regulated 1
5251	PHEX	phosphate regulating endopeptidase homolog, X-linked
79762	C1orf115	chromosome 1 open reading frame 115
2199	FBLN2	fibulin 2
653509	SFTPA1	surfactant protein A1
7098	TLR3	toll-like receptor 3
4175	MCM6	minichromosome maintenance complex component 6
653567	FAM23A	family with sequence similarity 23, member A
79007	DBNDD1	dysbindin (dystrobrevin binding protein 1) domain containing 1
5168	ENPP2	ectonucleotide pyrophosphatase/phosphodiesterase 2
81552	VOPP1	vesicular, overexpressed in cancer, prosurvival protein 1
2833	CXCR3	chemokine (C-X-C motif) receptor 3
55964	SEPT3	septin 3
8506	CNTNAP1	contactin associated protein 1
23122	CLASP2	cytoplasmic linker associated protein 2
1573	CYP2J2	cytochrome P450, family 2, subfamily J, polypeptide 2
3486	IGFBP3	insulin-like growth factor binding protein 3
11326	VSIG4	V-set and immunoglobulin domain containing 4
165	AEBP1	AE binding protein 1
9892	SNAP91	synaptosomal-associated protein, 91kDa homolog (mouse)
55728	N4BP2	NEDD4 binding protein 2
946	SIGLEC6	sialic acid binding Ig-like lectin 6
10911	UTS2	urotensin 2
282618	IL29	interleukin 29 (interferon, lambda 1)
2781	GNAZ	guanine nucleotide binding protein (G protein), alpha z polypeptide
222161	DKFZP586I1420	hypothetical protein DKFZp586I1420
81620	CDT1	chromatin licensing and DNA replication factor 1
6689	SPIB	Spi-B transcription factor (Spi-1/PU.1 related)
116028	C16orf75	chromosome 16 open reading frame 75
152485	ZNF827	zinc finger protein 827

10572	SIVA1	SIVA1, apoptosis-inducing factor
1501	CTNND2	catenin (cadherin-associated protein), delta 2 (neural plakophilin-related arm-repeat protein)
1593	CYP27A1	cytochrome P450, family 27, subfamily A, polypeptide 1
257144	GCET2	germinal center expressed transcript 2
84433	CARD11	caspase recruitment domain family, member 11
1428	CRYM	crystallin, mu
132884	EVC2	Ellis van Creveld syndrome 2
55691	FRMD4A	FERM domain containing 4A
5358	PLS3	plastin 3
6347	CCL2	chemokine (C-C motif) ligand 2
4130	MAP1A	microtubule-associated protein 1A
387882	C12orf75	chromosome 12 open reading frame 75
8322	FZD4	frizzled homolog 4 (Drosophila)
55266	TMEM19	transmembrane protein 19
79705	LRRK1	leucine-rich repeat kinase 1
89765	RSPH1	radial spoke head 1 homolog (Chlamydomonas)
3512	IGJ	immunoglobulin J polypeptide, linker protein for immunoglobulin alpha and mu polypeptides
1373	CPS1	carbamoyl-phosphate synthase 1, mitochondrial
4320	MMP11	matrix metallopeptidase 11 (stromelysin 3)
50489	CD207	CD207 molecule, langerin
55068	ENOX1	ecto-NOX disulfide-thiol exchanger 1
64084	CLSTN2	calsyntenin 2
25925	ZNF521	zinc finger protein 521
22915	MMRN1	multimerin 1
9957	HS3ST1	heparan sulfate (glucosamine) 3-O-sulfotransferase 1
5129	CDK18	cyclin-dependent kinase 18
116159	CYYR1	cysteine/tyrosine-rich 1
728715	LOC728715	ovostatin homolog 2-like
7710	ZNF154	zinc finger protein 154
79971	WLS	wntless homolog (Drosophila)
84057	MND1	meiotic nuclear divisions 1 homolog (S. cerevisiae)
4923	NTSR1	neurotensin receptor 1 (high affinity)
165140	OXER1	oxoeicosanoid (OXE) receptor 1
170482	CLEC4C	C-type lectin domain family 4, member C
23705	CADM1	cell adhesion molecule 1
5754	PTK7	PTK7 protein tyrosine kinase 7
8836	GGH	gamma-glutamyl hydrolase (conjugase, folylpolygammaglutamyl hydrolase)
30835	CD209	CD209 molecule
3581	IL9R	interleukin 9 receptor
163702	IL28RA	interleukin 28 receptor, alpha (interferon, lambda receptor)
199731	CADM4	cell adhesion molecule 4
55655	NLRP2	NLR family, pyrin domain containing 2
26022	TMEM98	transmembrane protein 98
3741	KCNA5	potassium voltage-gated channel, shaker-related subfamily, member 5
116449	CLNK	cytokine-dependent hematopoietic cell linker
100505650	LOC100505650	hypothetical LOC100505650
58499	ZNF462	zinc finger protein 462
219902	TMEM136	transmembrane protein 136
3852	KRT5	keratin 5
22837	COBLL1	COBL-like 1
83988	NCALD	neurocalcin delta
2047	EPHB1	EPH receptor B1
55653	BCAS4	breast carcinoma amplified sequence 4
8406	SRPX	sushi-repeat containing protein, X-linked
113451	ADC	arginine decarboxylase
8115	TCL1A	T-cell leukemia/lymphoma 1A
1000	CDH2	cadherin 2, type 1, N-cadherin (neuronal)
2213	FCGR2B	Fc fragment of IgG, low affinity IIb, receptor (CD32)
56961	SHD	Src homology 2 domain containing transforming protein D
91319	DERL3	Der1-like domain family, member 3
29993	PACSIN1	protein kinase C and casein kinase substrate in neurons 1
2882	GPX7	glutathione peroxidase 7
83716	CRISPLD2	cysteine-rich secretory protein LCCL domain containing 2
445328	ARHGEF35	Rho guanine nucleotide exchange factor (GEF) 35
22874	PLEKHA6	pleckstrin homology domain containing, family A member 6
343990	C2orf55	chromosome 2 open reading frame 55
23316	CUX2	cut-like homeobox 2
389816	LRRC26	leucine rich repeat containing 26
153769	SH3RF2	SH3 domain containing ring finger 2
1815	DRD4	dopamine receptor D4
23205	ACSBG1	acyl-CoA synthetase bubblegum family member 1
54836	BSPRY	B-box and SPRY domain containing
5368	PNOC	prepronociceptin
5730	PTGDS	prostaglandin D2 synthase 21kDa (brain)
1903	S1PR3	sphingosine-1-phosphate receptor 3
434	ASIP	agouti signaling protein

pDC (upregulated genes)

ENTREZ ID	Gene Symbol	Gene Name
3002	GZMB	granzyme B (granzyme 2, cytotoxic T-lymphocyte-associated serine esterase 1)
29993	PACSIN1	protein kinase C and casein kinase substrate in neurons 1
2317	FLNB	filamin B, beta
89932	PAPLN	papilin, proteoglycan-like sulfated glycoprotein
57026	PDXP	pyridoxal (pyridoxine, vitamin B6) phosphatase
2788	GNG7	guanine nucleotide binding protein (G protein), gamma 7
5730	PTGDS	prostaglandin D2 synthase 21kDa (brain)
23231	SEL1L3	sel-1 suppressor of lin-12-like 3 (C. elegans)
22837	COBLL1	COBL-like 1
163702	IL28RA	interleukin 28 receptor, alpha (interferon, lambda receptor)
221188	GPR114	G protein-coupled receptor 114
5176	SERPINF1	serpin peptidase inhibitor, clade F (alpha-2 antiplasmin, pigment epithelium derived factor), member 1
165	AEBP1	AE binding protein 1
29919	C18orf8	chromosome 18 open reading frame 8
2833	CXCR3	chemokine (C-X-C motif) receptor 3
55214	LEPREL1	leprecan-like 1

51754	TMEM8B	transmembrane protein 8B
8644	AKR1C3	aldo-keto reductase family 1, member C3 (3-alpha hydroxysteroid dehydrogenase, type II)
10184	LHFPL2	lipoma HMGIC fusion partner-like 2
3665	IRF7	interferon regulatory factor 7
3741	KCNA5	potassium voltage-gated channel, shaker-related subfamily, member 5
7169	TPM2	tropomyosin 2 (beta)
8408	ULK1	unc-51-like kinase 1 (C. elegans)
23390	ZDHHC17	zinc finger, DHHC-type containing 17
51237	MZB1	marginal zone B and B1 cell-specific protein
9783	RIMS3	regulating synaptic membrane exocytosis 3
170482	CLEC4C	C-type lectin domain family 4, member C
84433	CARD11	caspase recruitment domain family, member 11
1428	CRYM	crystallin, mu
1601	DAB2	disabled homolog 2, mitogen-responsive phosphoprotein (Drosophila)
2047	EPHB1	EPH receptor B1
387882	C12orf75	chromosome 12 open reading frame 75
434	ASIP	agouti signaling protein
23316	CUX2	cut-like homeobox 2
5624	PROC	protein C (inactivator of coagulation factors Va and VIIIa)
9637	FEZ2	fasciculation and elongation protein zeta 2 (zygin II)
4320	MMP11	matrix metallopeptidase 11 (stromelysin 3)
57559	STAMBPL1	STAM binding protein-like 1
4925	NUCB2	nucleobindin 2
23677	SH3BP4	SH3-domain binding protein 4
1743	DLST	dihydrolipoamide S-succinyltransferase (E2 component of 2-oxo-glutarate complex)
51655	RASD1	RAS, dexamethasone-induced 1
999	CDH1	cadherin 1, type 1, E-cadherin (epithelial)
5519	PPP2R1B	protein phosphatase 2, regulatory subunit A, beta
389816	LRRC26	leucine rich repeat containing 26
7915	ALDH5A1	aldehyde dehydrogenase 5 family, member A1
90313	TP53I13	tumor protein p53 inducible protein 13
54806	AHI1	Abelson helper integration site 1
4130	MAP1A	microtubule-associated protein 1A
3925	STMN1	stathmin 1
54836	BSPRY	B-box and SPRY domain containing
113179	ADAT3	adenosine deaminase, tRNA-specific 3, TAD3 homolog (S. cerevisiae)
84875	PARP10	poly (ADP-ribose) polymerase family, member 10
8115	TCL1A	T-cell leukemia/lymphoma 1A
115294	PCMTD1	protein-L-isoaspartate (D-aspartate) O-methyltransferase domain containing 1
55086	CXorf57	chromosome X open reading frame 57
1602	DACH1	dachshund homolog 1 (Drosophila)
7976	FZD3	frizzled homolog 3 (Drosophila)
2882	GPX7	glutathione peroxidase 7
6484	ST3GAL4	ST3 beta-galactoside alpha-2,3-sialyltransferase 4
23205	ACSBG1	acyl-CoA synthetase bubblegum family member 1
9623	TCL1B	T-cell leukemia/lymphoma 1B
8481	OFD1	oral-facial-digital syndrome 1
3512	IGJ	immunoglobulin J polypeptide, linker protein for immunoglobulin alpha and mu polypeptides
860	RUNX2	runt-related transcription factor 2
56961	SHD	Src homology 2 domain containing transforming protein D
116159	CYYR1	cysteine/tyrosine-rich 1
26003	GORASP2	golgi reassembly stacking protein 2, 55kDa
79690	GAL3ST4	galactose-3-O-sulfotransferase 4
1633	DCK	deoxycytidine kinase
79713	TMEM149	transmembrane protein 149
4311	MME	membrane metallo-endopeptidase
27143	KIAA1274	KIAA1274
55770	EXOC2	exocyst complex component 2
4209	MEF2D	myocyte enhancer factor 2D
113451	ADC	arginine decarboxylase
81571	NCRNA00287	non-protein coding RNA 287
26002	MOXD1	monooxygenase, DBH-like 1
587	BCAT2	branched chain amino-acid transaminase 2, mitochondrial
10113	PREB	prolactin regulatory element binding
7226	TRPM2	transient receptor potential cation channel, subfamily M, member 2
27352	SGSM3	small G protein signaling modulator 3
84707	BEX2	brain expressed X-linked 2
54207	KCNK10	potassium channel, subfamily K, member 10
23592	LEMD3	LEM domain containing 3
2273	FHL1	four and a half LIM domains 1
29115	SAP30BP	SAP30 binding protein
10572	SIVA1	SIVA1, apoptosis-inducing factor
64764	CREB3L2	cAMP responsive element binding protein 3-like 2
283673	NCRNA00277	non-protein coding RNA 277
57576	KIF17	kinesin family member 17
22915	MMRN1	multimerin 1
221322	C6orf170	chromosome 6 open reading frame 170
121053	C12orf45	chromosome 12 open reading frame 45
2529	FUT7	fucosyltransferase 7 (alpha (1,3) fucosyltransferase)
4926	NUMA1	nuclear mitotic apparatus protein 1
7434	VIPR2	vasoactive intestinal peptide receptor 2
92922	CCDC102A	coiled-coil domain containing 102A
199731	CADM4	cell adhesion molecule 4
27090	ST6GALNAC4	ST6 (alpha-N-acetyl-neuraminyl-2,3-beta-galactosyl-1,3)-N-acetylgalactosaminide alpha-2,6-sialyltransferase 4
23234	DNAJC9	DnaJ (Hsp40) homolog, subfamily C, member 9
145508	C14orf145	chromosome 14 open reading frame 145
55624	POMGNT1	protein O-linked mannose beta1,2-N-acetylglucosaminyltransferase
25994	HIGD1A	HIG1 hypoxia inducible domain family, member 1A
57185	NIPAL3	NIPA-like domain containing 3
84969	TOX2	TOX high mobility group box family member 2
29760	BLNK	B-cell linker
29927	SEC61A1	Sec61 alpha 1 subunit (S. cerevisiae)
222166	C7orf41	chromosome 7 open reading frame 41
157285	SGK223	homolog of rat pragma of Rnd2
501	ALDH7A1	aldehyde dehydrogenase 7 family, member A1

54442	KCTD5	potassium channel tetramerisation domain containing 5
4953	ODC1	ornithine decarboxylase 1
9588	PRDX6	peroxiredoxin 6
5168	ENPP2	ectonucleotide pyrophosphatase/phosphodiesterase 2
3682	ITGAE	integrin, alpha E (antigen CD103, human mucosal lymphocyte antigen 1; alpha polypeptide)
7710	ZNF154	zinc finger protein 154
55830	GLT8D1	glycosyltransferase 8 domain containing 1
91319	DERL3	Der1-like domain family, member 3
23242	COBL	cordon-bleu homolog (mouse)
152137	CCDC50	coiled-coil domain containing 50
84171	LOXL4	lysyl oxidase-like 4
80326	WNT10A	wingless-type MMTV integration site family, member 10A
9855	FARP2	FERM, RhoGEF and pleckstrin domain protein 2
10858	CYP46A1	cytochrome P450, family 46, subfamily A, polypeptide 1
9973	CCS	copper chaperone for superoxide dismutase
9806	SPOCK2	sparc/osteonectin, cwcv and kazal-like domains proteoglycan (testican) 2
725	C4BPB	complement component 4 binding protein, beta
11339	OIP5	Opa interacting protein 5
29801	ZDHHC8	zinc finger, DHHC-type containing 8
80183	C13orf18	chromosome 13 open reading frame 18
64766	S100PBP	S100P binding protein
339983	NAT8L	N-acetyltransferase 8-like (GCN5-related, putative)
79567	FAM65A	family with sequence similarity 65, member A
84705	GTPBP3	GTP binding protein 3 (mitochondrial)
54749	EPDR1	ependymin related protein 1 (zebrafish)
78999	LRFN4	leucine rich repeat and fibronectin type III domain containing 4
5297	PI4KA	phosphatidylinositol 4-kinase, catalytic, alpha
6925	TCF4	transcription factor 4
9709	HERPUD1	homocysteine-inducible, endoplasmic reticulum stress-inducible, ubiquitin-like domain member 1
4041	LRP5	low density lipoprotein receptor-related protein 5
55083	KIF26B	kinesin family member 26B
8473	OGT	O-linked N-acetylglucosamine (GlcNAc) transferase (UDP-N-acetylglucosamine:polypeptide-N-acetylglucosaminyl transferase)
550112	LOC550112	hypothetical LOC550112
2931	GSK3A	glycogen synthase kinase 3 alpha
255631	COL24A1	collagen, type XXIV, alpha 1
90060	CCDC120	coiled-coil domain containing 120
8237	USP11	ubiquitin specific peptidase 11
493	ATP2B4	ATPase, Ca++ transporting, plasma membrane 4
23189	KANK1	KN motif and ankyrin repeat domains 1
6196	RPS6KA2	ribosomal protein S6 kinase, 90kDa, polypeptide 2
4799	NFX1	nuclear transcription factor, X-box binding 1
9725	TMEM63A	transmembrane protein 63A
9537	TP53I11	tumor protein p53 inducible protein 11
3852	KRT5	keratin 5
728715	LOC728715	ovostatin homolog 2-like
116238	TLCD1	TLC domain containing 1
4175	MCM6	minichromosome maintenance complex component 6
27004	TCL6	T-cell leukemia/lymphoma 6 (non-protein coding)
91010	FMNL3	formin-like 3
25894	PLEKHG4	pleckstrin homology domain containing, family G (with RhoGef domain) member 4
924	CD7	CD7 molecule
28392	IGHV4-59	immunoglobulin heavy variable 4-59
729786	GOLGA8C	golgin A8 family, member C
11320	MGAT4A	mannosyl (alpha-1,3-)-glycoprotein beta-1,4-N-acetylglucosaminyltransferase, isozyme A
6000	RGS7	regulator of G-protein signaling 7
728743	LOC728743	similar to GLI-Kruppel family member HKR1
81539	SLC38A1	solute carrier family 38, member 1
56478	EIF4ENIF1	eukaryotic translation initiation factor 4E nuclear import factor 1
6335	SCN9A	sodium channel, voltage-gated, type IX, alpha subunit
5457	POU4F1	POU class 4 homeobox 1
1815	DRD4	dopamine receptor D4
81606	LBH	limb bud and heart development homolog (mouse)
375	ARF1	ADP-ribosylation factor 1
79805	VASH2	vasohibin 2
2055	CLN8	ceroid-lipofuscinosis, neuronal 8 (epilepsy, progressive with mental retardation)
56926	NCLN	nicalin
55526	DHTKD1	dehydrogenase E1 and transketolase domain containing 1
55966	AJAP1	adherens junctions associated protein 1
127262	TPRG1L	tumor protein p63 regulated 1-like
1271	CNTFR	ciliary neurotrophic factor receptor
347918	EP400NL	EP400 N-terminal like
22872	SEC31A	SEC31 homolog A (S. cerevisiae)
221692	PHACTR1	phosphatase and actin regulator 1
286256	LCN12	lipocalin 12
9601	PDIA4	protein disulfide isomerase family A, member 4
60673	C12orf44	chromosome 12 open reading frame 44
8575	PRKRA	protein kinase, interferon-inducible double stranded RNA dependent activator
8506	CNTNAP1	contactin associated protein 1
10009	ZBTB33	zinc finger and BTB domain containing 33
23394	ADNP	activity-dependent neuroprotector homeobox
285268	ZNF621	zinc finger protein 621
89781	HPS4	Hermansky-Pudlak syndrome 4
4047	LSS	lanosterol synthase (2,3-oxidosqualene-lanosterol cyclase)
51104	FAM108B1	family with sequence similarity 108, member B1
23002	DAAM1	dishevelled associated activator of morphogenesis 1
222161	DKFZP586I1420	hypothetical protein DKFZp586I1420
55342	STRBP	spermatid perinuclear RNA binding protein
51339	DACT1	dapper, antagonist of beta-catenin, homolog 1 (Xenopus laevis)
9197	SLC33A1	solute carrier family 33 (acetyl-CoA transporter), member 1
51257	02-Mar	membrane-associated ring finger (C3HC4) 2
7728	ZNF175	zinc finger protein 175
64207	IRF2BPL	interferon regulatory factor 2 binding protein-like
23435	TARDBP	TAR DNA binding protein
100128242	LOC100128242	hypothetical LOC100128242
51635	DHRS7	dehydrogenase/reductase (SDR family) member 7

55266	TMEM19	transmembrane protein 19
3775	KCNK1	potassium channel, subfamily K, member 1
4121	MAN1A1	mannosidase, alpha, class 1A, member 1
2819	GPD1	glycerol-3-phosphate dehydrogenase 1 (soluble)
51239	ANKRD39	ankyrin repeat domain 39
5754	PTK7	PTK7 protein tyrosine kinase 7
7371	UCK2	uridine-cytidine kinase 2
8406	SRPX	sushi-repeat containing protein, X-linked
7392	USF2	upstream transcription factor 2, c-fos interacting
8836	GGH	gamma-glutamyl hydrolase (conjugase, folylpolygammaglutamyl hydrolase)
11006	LILRB4	leukocyte immunoglobulin-like receptor, subfamily B (with TM and ITIM domains), member 4
29903	CCDC106	coiled-coil domain containing 106
56061	UBFD1	ubiquitin family domain containing 1
3338	DNAJC4	DnaJ (Hsp40) homolog, subfamily C, member 4
5251	PHEX	phosphate regulating endopeptidase homolog, X-linked
26249	KLHL3	kelch-like 3 (Drosophila)
9366	RAB9BP1	RAB9B, member RAS oncogene family pseudogene 1
9991	ROD1	ROD1 regulator of differentiation 1 (S. pombe)
150159	NHEDC1	Na+/H+ exchanger domain containing 1
22929	SEPHS1	selenophosphate synthetase 1
8392	OR3A3	olfactory receptor, family 3, subfamily A, member 3
922	CD5L	CD5 molecule-like
9963	SLC23A1	solute carrier family 23 (nucleobase transporters), member 1
6734	SRPR	signal recognition particle receptor (docking protein)
284459	HKR1	HKR1, GLI-Kruppel zinc finger family member
54106	TLR9	toll-like receptor 9
283932	NCRNA00095	non-protein coding RNA 95
286527	TMSB15B	thymosin beta 15B
9044	BTAF1	BTAF1 RNA polymerase II, B-TFIID transcription factor-associated, 170kDa (Mot1 homolog, S. cerevisiae)
80765	STARD5	StAR-related lipid transfer (START) domain containing 5
9501	RPH3AL	rabphilin 3A-like (without C2 domains)
54843	SYTL2	synaptotagmin-like 2
6574	SLC20A1	solute carrier family 20 (phosphate transporter), member 1
140686	WFDC3	WAP four-disulfide core domain 3
8425	LTBP4	latent transforming growth factor beta binding protein 4
54872	PIGG	phosphatidylinositol glycan anchor biosynthesis, class G
440423	SUZ12P	suppressor of zeste 12 homolog pseudogene
5425	POLD2	polymerase (DNA directed), delta 2, regulatory subunit 50kDa
79791	FBXO31	F-box protein 31
165055	CCDC138	coiled-coil domain containing 138
10263	CDK2AP2	cyclin-dependent kinase 2 associated protein 2
223082	ZNRF2	zinc and ring finger 2
53841	CDHR5	cadherin-related family member 5
51678	MPP6	membrane protein, palmitoylated 6 (MAGUK p55 subfamily member 6)
9727	RAB11FIP3	RAB11 family interacting protein 3 (class II)
22955	SCMH1	sex comb on midleg homolog 1 (Drosophila)
196394	AMN1	antagonist of mitotic exit network 1 homolog (S. cerevisiae)
286102	TMED10P1	transmembrane emp24-like trafficking protein 10 (yeast) pseudogene 1
54869	EPS8L1	EPS8-like 1
51303	FKBP11	FK506 binding protein 11, 19 kDa
6689	SPIB	Spi-B transcription factor (Spi-1/PU.1 related)
65985	AACS	acetoacetyl-CoA synthetase
22807	IKZF2	IKAROS family zinc finger 2 (Helios)
171558	PTCRA	pre T-cell antigen receptor alpha
10681	GNB5	guanine nucleotide binding protein (G protein), beta 5
6242	RTKN	rhotekin
5208	PFKFB2	6-phosphofructo-2-kinase/fructose-2,6-biphosphatase 2
66035	SLC2A11	solute carrier family 2 (facilitated glucose transporter), member 11
6117	RPA1	replication protein A1, 70kDa
23157	SEPT6	septin 6
359948	IRF2BP2	interferon regulatory factor 2 binding protein 2
142678	MIB2	mindbomb homolog 2 (Drosophila)
80305	TRABD	TraB domain containing
80063	ATF7IP2	activating transcription factor 7 interacting protein 2
23111	SPG20	spastic paraplegia 20 (Troyer syndrome)
642938	FAM196A	family with sequence similarity 196, member A
124590	USH1G	Usher syndrome 1G (autosomal recessive)
157567	ANKRD46	ankyrin repeat domain 46
10959	TMED2	transmembrane emp24 domain trafficking protein 2
152926	PPM1K	protein phosphatase, Mg2+/Mn2+ dependent, 1K
10985	GCN1L1	GCN1 general control of amino-acid synthesis 1-like 1 (yeast)
441024	MTHFD2L	methylenetetrahydrofolate dehydrogenase (NADP+ dependent) 2-like
54760	PCSK4	proprotein convertase subtilisin/kexin type 4
85026	C9orf37	chromosome 9 open reading frame 37
7570	ZNF22	zinc finger protein 22 (KOX 15)
79710	MORC4	MORC family CW-type zinc finger 4
81608	FIP1L1	FIP1 like 1 (S. cerevisiae)
55829	SELS	selenoprotein S
115825	WDFY2	WD repeat and FYVE domain containing 2
26472	PPP1R14B	protein phosphatase 1, regulatory (inhibitor) subunit 14B
57577	KIAA1407	KIAA1407
2205	FCER1A	Fc fragment of IgE, high affinity I, receptor for; alpha polypeptide
134	ADORA1	adenosine A1 receptor
3703	STT3A	STT3, subunit of the oligosaccharyltransferase complex, homolog A (S. cerevisiae)
7283	TUBG1	tubulin, gamma 1
197335	WDR90	WD repeat domain 90
84174	SLA2	Src-like-adaptor 2
9375	TM9SF2	transmembrane 9 superfamily member 2
55082	ARGLU1	arginine and glutamate rich 1
118	ADD1	adducin 1 (alpha)
29803	REPIN1	replication initiator 1
84617	TUBB6	tubulin, beta 6
6302	TSPAN31	tetraspanin 31
100133224	LOC100133224	hypothetical protein LOC100133224
23061	TBC1D9B	TBC1 domain family, member 9B (with GRAM domain)

93663	ARHGAP18	Rho GTPase activating protein 18
11149	BVES	blood vessel epicardial substance
27175	TUBG2	tubulin, gamma 2
51559	NT5DC3	5'-nucleotidase domain containing 3
51060	TXNDC12	thioredoxin domain containing 12 (endoplasmic reticulum)
57117	INTS12	integrator complex subunit 12
23165	NUP205	nucleoporin 205kDa
27128	CYTH4	cytohesin 4
710	SERPING1	serpin peptidase inhibitor, clade G (C1 inhibitor), member 1
11164	NUDT5	nudix (nucleoside diphosphate linked moiety X)-type motif 5
203197	C9orf91	chromosome 9 open reading frame 91
84080	C16orf48	chromosome 16 open reading frame 48
85451	UNK	unkempt homolog (Drosophila)
5436	POLR2G	polymerase (RNA) II (DNA directed) polypeptide G
8459	TPST2	tyrosylprotein sulfotransferase 2
81932	HDHD3	haloacid dehalogenase-like hydrolase domain containing 3
100288273	LOC100288273	hypothetical LOC100288273
9744	ACAP1	ArfGAP with coiled-coil, ankyrin repeat and PH domains 1
23030	KDM4B	lysine (K)-specific demethylase 4B
25915	NDUFAF3	NADH dehydrogenase (ubiquinone) 1 alpha subcomplex, assembly factor 3
29100	TMEM208	transmembrane protein 208
1798	DPAGT1	dolichyl-phosphate (UDP-N-acetylglucosamine) N-acetylglucosaminephosphotransferase 1 (GlcNAc-1-P transferase)
946	SIGLEC6	sialic acid binding Ig-like lectin 6
440	ASNS	asparagine synthetase (glutamine-hydrolyzing)
83941	TM2D1	TM2 domain containing 1
5368	PNOC	prepronociceptin
100506144	LOC100506144	hypothetical protein LOC100506144
122509	IFI27L1	interferon, alpha-inducible protein 27-like 1
92973	LOC92973	hypothetical LOC92973
286367	LOC286367	FP944
25800	SLC39A6	solute carrier family 39 (zinc transporter), member 6
57823	SLAMF7	SLAM family member 7
55353	LAPTM4B	lysosomal protein transmembrane 4 beta
11340	EXOSC8	exosome component 8
5424	POLD1	polymerase (DNA directed), delta 1, catalytic subunit 125kDa
442421	LOC442421	hypothetical LOC442421
57533	TBC1D14	TBC1 domain family, member 14
64784	CRTC3	CREB regulated transcription coactivator 3
1803	DPP4	dipeptidyl-peptidase 4
51011	FAHD2A	fumarylacetoacetate hydrolase domain containing 2A
5496	PPM1G	protein phosphatase, Mg2+/Mn2+ dependent, 1G
5800	PTPRO	protein tyrosine phosphatase, receptor type, O
60343	FAM3A	family with sequence similarity 3, member A
26354	GNL3	guanine nucleotide binding protein-like 3 (nucleolar)
5274	SERPINI1	serpin peptidase inhibitor, clade I (neuroserpin), member 1
1163	CKS1B	CDC28 protein kinase regulatory subunit 1B
5332	PLCB4	phospholipase C, beta 4
57176	VARS2	valyl-tRNA synthetase 2, mitochondrial (putative)
26512	INTS6	integrator complex subunit 6
6558	SLC12A2	solute carrier family 12 (sodium/potassium/chloride transporters), member 2
3551	IKBKB	inhibitor of kappa light polypeptide gene enhancer in B-cells, kinase beta
642	BLMH	bleomycin hydrolase
1394	CRHR1	corticotropin releasing hormone receptor 1
113878	DTX2	deltex homolog 2 (Drosophila)
10090	UST	uronyl-2-sulfotransferase
80758	PRR7	proline rich 7 (synaptic)
56271	BEX4	brain expressed, X-linked 4
755	C21orf2	chromosome 21 open reading frame 2
55748	CNDP2	CNDP dipeptidase 2 (metallopeptidase M20 family)
11332	ACOT7	acyl-CoA thioesterase 7
9692	KIAA0391	KIAA0391
343477	HSP90B3P	heat shock protein 90kDa beta (Grp94), member 3 (pseudogene)
1969	EPHA2	EPH receptor A2
6297	SALL2	sal-like 2 (Drosophila)
5358	PLS3	plastin 3
4649	MYO9A	myosin IXA
151888	BTLA	B and T lymphocyte associated
282763	OR51B5	olfactory receptor, family 51, subfamily B, member 5
56922	MCCC1	methylcrotonoyl-CoA carboxylase 1 (alpha)
728975	LOC728975	hypothetical LOC728975
2021	ENDOG	endonuclease G
23158	TBC1D9	TBC1 domain family, member 9 (with GRAM domain)
5747	PTK2	PTK2 protein tyrosine kinase 2
151313	FAHD2B	fumarylacetoacetate hydrolase domain containing 2B
22820	COPG	coatomer protein complex, subunit gamma
79085	SLC25A23	solute carrier family 25 (mitochondrial carrier; phosphate carrier), member 23
80212	CCDC92	coiled-coil domain containing 92
54897	CASZ1	castor zinc finger 1
6622	SNCA	synuclein, alpha (non A4 component of amyloid precursor)
131474	CHCHD4	coiled-coil-helix-coiled-coil-helix domain containing 4
6202	RPS8	ribosomal protein S8
60436	TGIF2	TGFB-induced factor homeobox 2
402	ARL2	ADP-ribosylation factor-like 2
109	ADCY3	adenylate cyclase 3
9404	LPXN	leupaxin
196051	PPAPDC1A	phosphatidic acid phosphatase type 2 domain containing 1A
3006	HIST1H1C	histone cluster 1, H1c
2926	GRSF1	G-rich RNA sequence binding factor 1
79705	LRRK1	leucine-rich repeat kinase 1
9978	RBX1	ring-box 1, E3 ubiquitin protein ligase
58505	OSTC	oligosaccharyltransferase complex subunit
147495	APCDD1	adenomatosis polyposis coli down-regulated 1
8883	NAE1	NEDD8 activating enzyme E1 subunit 1
5341	PLEK	pleckstrin
51114	ZDHHC9	zinc finger, DHHC-type containing 9

2322	FLT3	fms-related tyrosine kinase 3
54414	SIAE	sialic acid acetylesterase
56851	C15orf24	chromosome 15 open reading frame 24
7045	TGFBI	transforming growth factor, beta-induced, 68kDa
1603	DAD1	defender against cell death 1
2876	GPX1	glutathione peroxidase 1
170692	ADAMTS18	ADAM metallopeptidase with thrombospondin type 1 motif, 18
2287	FKBP3	FK506 binding protein 3, 25kDa
201895	C4orf34	chromosome 4 open reading frame 34
84293	C10orf58	chromosome 10 open reading frame 58
27240	SIT1	signaling threshold regulating transmembrane adaptor 1
641649	TMEM91	transmembrane protein 91
166614	DCLK2	doublecortin-like kinase 2
79443	FYCO1	FYVE and coiled-coil domain containing 1
6208	RPS14	ribosomal protein S14
7411	VBP1	von Hippel-Lindau binding protein 1
9117	SEC22C	SEC22 vesicle trafficking protein homolog C (S. cerevisiae)
27130	INVS	inversin
2030	SLC29A1	solute carrier family 29 (nucleoside transporters), member 1
170960	ZNF721	zinc finger protein 721
3111	HLA-DOA	major histocompatibility complex, class II, DO alpha
5134	PDCD2	programmed cell death 2
23303	KIF13B	kinesin family member 13B
5037	PEBP1	phosphatidylethanolamine binding protein 1
2194	FASN	fatty acid synthase
1019	CDK4	cyclin-dependent kinase 4
9804	TOMM20	translocase of outer mitochondrial membrane 20 homolog (yeast)
79174	CRELD2	cysteine-rich with EGF-like domains 2
30968	STOML2	stomatin (EPB72)-like 2
641638	SNHG6	small nucleolar RNA host gene 6 (non-protein coding)
10576	CCT2	chaperonin containing TCP1, subunit 2 (beta)
8564	KMO	kynurenine 3-monooxygenase (kynurenine 3-hydroxylase)
256586	LYSMD2	LysM, putative peptidoglycan-binding, domain containing 2
51602	NOP58	NOP58 ribonucleoprotein homolog (yeast)
6235	RPS29	ribosomal protein S29
5445	PON2	paraoxonase 2
57037	ANKMY2	ankyrin repeat and MYND domain containing 2
6640	SNTA1	syntrophin, alpha 1 (dystrophin-associated protein A1, 59kDa, acidic component)
6206	RPS12	ribosomal protein S12
81853	TMEM14B	transmembrane protein 14B
26060	APPL1	adaptor protein, phosphotyrosine interaction, PH domain and leucine zipper containing 1
10455	PECI	peroxisomal D3,D2-enoyl-CoA isomerase
23244	PDS5A	PDS5, regulator of cohesion maintenance, homolog A (S. cerevisiae)
3916	LAMP1	lysosomal-associated membrane protein 1

pDC (downregulated genes)

ENTREZ ID	Gene Symbol	Gene Name
1318	SLC31A2	solute carrier family 31 (copper transporters), member 2
126014	OSCAR	osteoclast associated, immunoglobulin-like receptor
6252	RTN1	reticulon 1
408	ARRB1	arrestin, beta 1
10410	IFITM3	interferon induced transmembrane protein 3 (1-8U)
5265	SERPINA1	serpin peptidase inhibitor, clade A (alpha-1 antiproteinase, antitrypsin), member 1
51311	TLR8	toll-like receptor 8
9780	FAM38A	family with sequence similarity 38, member A
54438	GFOD1	glucose-fructose oxidoreductase domain containing 1
80727	TTYH3	tweety homolog 3 (Drosophila)
55509	BATF3	basic leucine zipper transcription factor, ATF-like 3
7791	ZYX	zyxin
1465	CSRP1	cysteine and glycine-rich protein 1
7764	ZNF217	zinc finger protein 217
55803	ADAP2	ArfGAP with dual PH domains 2
2720	GLB1	galactosidase, beta 1
3311	HSPA7	heat shock 70kDa protein 7 (HSP70B)
114769	CARD16	caspase recruitment domain family, member 16
11025	LILRB3	leukocyte immunoglobulin-like receptor, subfamily B (with TM and ITIM domains), member 3
9046	DOK2	docking protein 2, 56kDa
1102	RCBTB2	regulator of chromosome condensation (RCC1) and BTB (POZ) domain containing protein 2
2040	STOM	stomatin
27287	VENTX	VENT homeobox homolog (Xenopus laevis)
5732	PTGER2	prostaglandin E receptor 2 (subtype EP2), 53kDa
10981	RAB32	RAB32, member RAS oncogene family
10970	CKAP4	cytoskeleton-associated protein 4
79168	LILRA6	leukocyte immunoglobulin-like receptor, subfamily A (with TM domain), member 6
1051	CEBPB	CCAAT/enhancer binding protein (C/EBP), beta
7462	LAT2	linker for activation of T cells family, member 2
121551	BTBD11	BTB (POZ) domain containing 11
79901	CYBRD1	cytochrome b reductase 1
3098	HK1	hexokinase 1
10154	PLXNC1	plexin C1
5899	RALB	v-ral simian leukemia viral oncogene homolog B (ras related; GTP binding protein)
59	ACTA2	actin, alpha 2, smooth muscle, aorta
3176	HNMT	histamine N-methyltransferase
10439	OLFM1	olfactomedin 1
79720	VPS37B	vacuolar protein sorting 37 homolog B (S. cerevisiae)
4627	MYH9	myosin, heavy chain 9, non-muscle
10422	UBAC1	UBA domain containing 1
706	TSPO	translocator protein (18kDa)
64135	IFIH1	interferon induced with helicase C domain 1
6237	RRAS	related RAS viral (r-ras) oncogene homolog
2869	GRK5	G protein-coupled receptor kinase 5
2665	GDI2	GDP dissociation inhibitor 2
837	CASP4	caspase 4, apoptosis-related cysteine peptidase
137835	TMEM71	transmembrane protein 71
64411	ARAP3	ArfGAP with RhoGAP domain, ankyrin repeat and PH domain 3

3660	IRF2	interferon regulatory factor 2
375387	LRRC33	leucine rich repeat containing 33
55365	TMEM176A	transmembrane protein 176A
490	ATP2B1	ATPase, Ca++ transporting, plasma membrane 1
8462	KLF11	Kruppel-like factor 11
3490	IGFBP7	insulin-like growth factor binding protein 7
11275	KLHL2	kelch-like 2, Mayven (Drosophila)
11213	IRAK3	interleukin-1 receptor-associated kinase 3
91663	MYADM	myeloid-associated differentiation marker
53	ACP2	acid phosphatase 2, lysosomal
2634	GBP2	guanylate binding protein 2, interferon-inducible
23208	SYT11	synaptotagmin XI
7408	VASP	vasodilator-stimulated phosphoprotein
8495	PPFIBP2	PTPRF interacting protein, binding protein 2 (liprin beta 2)
1462	VCAN	versican
5719	PSMD13	proteasome (prosome, macropain) 26S subunit, non-ATPase, 13
3628	INPP1	inositol polyphosphate-1-phosphatase
80301	PLEKHO2	pleckstrin homology domain containing, family O member 2
2852	GPER	G protein-coupled estrogen receptor 1
5210	PFKFB4	6-phosphofructo-2-kinase/fructose-2,6-biphosphatase 4
4673	NAP1L1	nucleosome assembly protein 1-like 1
55129	ANO10	anoctamin 10
23240	KIAA0922	KIAA0922
57761	TRIB3	tribbles homolog 3 (Drosophila)
463	ZFHX3	zinc finger homeobox 3
64127	NOD2	nucleotide-binding oligomerization domain containing 2
54453	RIN2	Ras and Rab interactor 2
2534	FYN	FYN oncogene related to SRC, FGR, YES
5724	PTAFR	platelet-activating factor receptor
83666	PARP9	poly (ADP-ribose) polymerase family, member 9
2037	EPB41L2	erythrocyte membrane protein band 4.1-like 2
80256	KIAA1539	KIAA1539
79026	AHNAK	AHNAK nucleoprotein
6050	RNH1	ribonuclease/angiogenin inhibitor 1
7168	TPM1	tropomyosin 1 (alpha)
57182	ANKRD50	ankyrin repeat domain 50
6453	ITSN1	intersectin 1 (SH3 domain protein)
10096	ACTR3	ARP3 actin-related protein 3 homolog (yeast)
58475	MS4A7	membrane-spanning 4-domains, subfamily A, member 7
11033	ADAP1	ArfGAP with dual PH domains 1
146223	CMTM4	CKLF-like MARVEL transmembrane domain containing 4
55841	WWC3	WWC family member 3
51155	HN1	hematological and neurological expressed 1
834	CASP1	caspase 1, apoptosis-related cysteine peptidase (interleukin 1, beta, convertase)
3636	INPPL1	inositol polyphosphate phosphatase-like 1
57498	KIDINS220	kinase D-interacting substrate, 220kDa
2633	GBP1	guanylate binding protein 1, interferon-inducible
10908	PNPLA6	patatin-like phospholipase domain containing 6
929	CD14	CD14 molecule
9051	PSTPIP1	proline-serine-threonine phosphatase interacting protein 1
138151	NACC2	NACC family member 2, BEN and BTB (POZ) domain containing
10811	NOXA1	NADPH oxidase activator 1
54930	HAUS4	HAUS augmin-like complex, subunit 4
255919	TMEM188	transmembrane protein 188
8795	TNFRSF10B	tumor necrosis factor receptor superfamily, member 10b
64218	SEMA4A	sema domain, immunoglobulin domain(Ig), transmembrane domain(TM) and short cytoplasmic domain, (semaphorin) 4A
6821	SUOX	sulfite oxidase
3459	IFNGR1	interferon gamma receptor 1
2537	IFI6	interferon, alpha-inducible protein 6
6102	RP2	retinitis pigmentosa 2 (X-linked recessive)
84689	MS4A14	membrane-spanning 4-domains, subfamily A, member 14
404636	FAM45A	family with sequence similarity 45, member A
29992	PILRA	paired immunoglobin-like type 2 receptor alpha
3687	ITGAX	integrin, alpha X (complement component 3 receptor 4 subunit)
9960	USP3	ubiquitin specific peptidase 3
116985	ARAP1	ArfGAP with RhoGAP domain, ankyrin repeat and PH domain 1
284611	FAM102B	family with sequence similarity 102, member B
441455	LOC441455	makorin ring finger protein 1 pseudogene
824	CAPN2	calpain 2, (m/II) large subunit
55654	TMEM127	transmembrane protein 127
219855	SLC37A2	solute carrier family 37 (glycerol-3-phosphate transporter), member 2
4303	FOXO4	forkhead box O4
7384	UQCRC1	ubiquinol-cytochrome c reductase core protein I
84926	SPRYD3	SPRY domain containing 3
1050	CEBPA	CCAAT/enhancer binding protein (C/EBP), alpha
57190	SEPN1	selenoprotein N, 1
54103	PION	pigeon homolog (Drosophila)
9061	PAPSS1	3'-phosphoadenosine 5'-phosphosulfate synthase 1
147040	KCTD11	potassium channel tetramerisation domain containing 11
54625	PARP14	poly (ADP-ribose) polymerase family, member 14
25911	DPCD	deleted in primary ciliary dyskinesia homolog (mouse)
26118	WSB1	WD repeat and SOCS box containing 1
6993	DYNLT1	dynein, light chain, Tctex-type 1
6132	RPL8	ribosomal protein L8
79154	DHRS11	dehydrogenase/reductase (SDR family) member 11
8519	IFITM1	interferon induced transmembrane protein 1 (9-27)
8099	CDK2AP1	cyclin-dependent kinase 2 associated protein 1
64422	ATG3	ATG3 autophagy related 3 homolog (S. cerevisiae)
5862	RAB2A	RAB2A, member RAS oncogene family
26750	RPS6KC1	ribosomal protein S6 kinase, 52kDa, polypeptide 1
29948	OSGIN1	oxidative stress induced growth inhibitor 1
10170	DHRS9	dehydrogenase/reductase (SDR family) member 9
2014	EMP3	epithelial membrane protein 3
7456	WIPF1	WAS/WASL interacting protein family, member 1
3148	HMGB2	high-mobility group box 2

9997	SCO2	SCO cytochrome oxidase deficient homolog 2 (yeast)
7263	TST	thiosulfate sulfurtransferase (rhodanese)
10205	MPZL2	myelin protein zero-like 2
196383	RILPL2	Rab interacting lysosomal protein-like 2
293	SLC25A6	solute carrier family 25 (mitochondrial carrier; adenine nucleotide translocator), member 6
1871	E2F3	E2F transcription factor 3
29097	CNIH4	cornichon homolog 4 (Drosophila)
280655	C14orf19	immunoglobulin (CD79A) binding protein 1 pseudogene
5351	PLOD1	procollagen-lysine 1, 2-oxoglutarate 5-dioxygenase 1
11046	SLC35D2	solute carrier family 35, member D2
53346	TM6SF1	transmembrane 6 superfamily member 1
808	CALM3	calmodulin 3 (phosphorylase kinase, delta)
64326	RFWD2	ring finger and WD repeat domain 2
57118	CAMK1D	calcium/calmodulin-dependent protein kinase ID
55093	WDYHV1	WDYHV motif containing 1
3784	KCNQ1	potassium voltage-gated channel, KQT-like subfamily, member 1
22918	CD93	CD93 molecule
943	TNFRSF8	tumor necrosis factor receptor superfamily, member 8
1979	EIF4EBP2	eukaryotic translation initiation factor 4E binding protein 2
829	CAPZA1	capping protein (actin filament) muscle Z-line, alpha 1
83541	FAM110A	family with sequence similarity 110, member A
57616	TSHZ3	teashirt zinc finger homeobox 3
26136	TES	testis derived transcript (3 LIM domains)
8650	NUMB	numb homolog (Drosophila)
5912	RAP2B	RAP2B, member of RAS oncogene family
10581	IFITM2	interferon induced transmembrane protein 2 (1-8D)
9470	EIF4E2	eukaryotic translation initiation factor 4E family member 2
3759	KCNJ2	potassium inwardly-rectifying channel, subfamily J, member 2
88455	ANKRD13A	ankyrin repeat domain 13A
23608	MKRN1	makorin ring finger protein 1
23167	EFR3A	EFR3 homolog A (S. cerevisiae)
7170	TPM3	tropomyosin 3
4126	MANBA	mannosidase, beta A, lysosomal
8445	DYRK2	dual-specificity tyrosine-(Y)-phosphorylation regulated kinase 2
5165	PDK3	pyruvate dehydrogenase kinase, isozyme 3
3936	LCP1	lymphocyte cytosolic protein 1 (L-plastin)
91351	DDX60L	DEAD (Asp-Glu-Ala-Asp) box polypeptide 60-like
8772	FADD	Fas (TNFRSF6)-associated via death domain
51734	SEPX1	selenoprotein X, 1
115948	CCDC151	coiled-coil domain containing 151
5768	QSOX1	quiescin Q6 sulfhydryl oxidase 1
80896	NPL	N-acetylneuraminate pyruvate lyase (dihydrodipicolinate synthase)
1876	E2F6	E2F transcription factor 6
25979	DHRS7B	dehydrogenase/reductase (SDR family) member 7B
64114	TMBIM1	transmembrane BAX inhibitor motif containing 1
54507	ADAMTSL4	ADAMTS-like 4
845	CASQ2	calsequestrin 2 (cardiac muscle)
1534	CYB561	cytochrome b-561
23526	HMHA1	histocompatibility (minor) HA-1
2950	GSTP1	glutathione S-transferase pi 1
6778	STAT6	signal transducer and activator of transcription 6, interleukin-4 induced
909	CD1A	CD1a molecule
51092	SIDT2	SID1 transmembrane family, member 2
10924	SMPDL3A	sphingomyelin phosphodiesterase, acid-like 3A
5223	PGAM1	phosphoglycerate mutase 1 (brain)
3516	RBPJ	recombination signal binding protein for immunoglobulin kappa J region
80025	PANK2	pantothenate kinase 2
7099	TLR4	toll-like receptor 4
9213	XPR1	xenotropic and polytropic retrovirus receptor 1
85440	DOCK7	dedicator of cytokinesis 7
90007	MIDN	midnolin
6277	S100A6	S100 calcium binding protein A6
57142	RTN4	reticulon 4
8819	SAP30	Sin3A-associated protein, 30kDa
9446	GSTO1	glutathione S-transferase omega 1
56894	AGPAT3	1-acylglycerol-3-phosphate O-acyltransferase 3
144402	CPNE8	copine VIII
5689	PSMB1	proteasome (prosome, macropain) subunit, beta type, 1
246175	CNOT6L	CCR4-NOT transcription complex, subunit 6-like
60682	SMAP1	small ArfGAP 1
5728	PTEN	phosphatase and tensin homolog
4061	LY6E	lymphocyte antigen 6 complex, locus E
11261	CHP	calcium binding protein P22
115548	FCHO2	FCH domain only 2
2783	GNB2	guanine nucleotide binding protein (G protein), beta polypeptide 2
158747	MOSPD2	motile sperm domain containing 2
81556	C15orf44	chromosome 15 open reading frame 44
11018	TMED1	transmembrane emp24 protein transport domain containing 1
1139	CHRNA7	cholinergic receptor, nicotinic, alpha 7
10890	RAB10	RAB10, member RAS oncogene family
57590	WDFY1	WD repeat and FYVE domain containing 1
3688	ITGB1	integrin, beta 1 (fibronectin receptor, beta polypeptide, antigen CD29 includes MDF2, MSK12)
4609	MYC	v-myc myelocytomatosis viral oncogene homolog (avian)
3949	LDLR	low density lipoprotein receptor
3707	ITPKB	inositol 1,4,5-trisphosphate 3-kinase B
114548	NLRP3	NLR family, pyrin domain containing 3
84173	ELMOD3	ELMO/CED-12 domain containing 3
867	CBL	Cas-Br-M (murine) ecotropic retroviral transforming sequence
5595	MAPK3	mitogen-activated protein kinase 3
284	ANGPT1	angiopoietin 1
5664	PSEN2	presenilin 2 (Alzheimer disease 4)
4173	MCM4	minichromosome maintenance complex component 4
646214	LOC646214	p21 protein (Cdc42/Rac)-activated kinase 2 pseudogene
83693	HSDL1	hydroxysteroid dehydrogenase like 1
10092	ARPC5	actin related protein 2/3 complex, subunit 5, 16kDa

474338	SUMO1P3	SUMO1 pseudogene 3
2675	GFRA2	GDNF family receptor alpha 2
3957	LGALS2	lectin, galactoside-binding, soluble, 2
55022	PID1	phosphotyrosine interaction domain containing 1
157	ADRBK2	adrenergic, beta, receptor kinase 2
55839	CENPN	centromere protein N
283219	KCTD21	potassium channel tetramerisation domain containing 21
2995	GYPC	glycophorin C (Gerbich blood group)
2885	GRB2	growth factor receptor-bound protein 2
8761	PABPC4	poly(A) binding protein, cytoplasmic 4 (inducible form)
112812	FDX1L	ferredoxin 1-like
262	AMD1	adenosylmethionine decarboxylase 1
55911	APOBR	apolipoprotein B receptor
26184	OR1F2P	olfactory receptor, family 1, subfamily F, member 2
116988	AGAP3	ArfGAP with GTPase domain, ankyrin repeat and PH domain 3
6261	RYR1	ryanodine receptor 1 (skeletal)
9262	STK17B	serine/threonine kinase 17b
9935	MAFB	v-maf musculoaponeurotic fibrosarcoma oncogene homolog B (avian)
57600	FNIP2	folliculin interacting protein 2
161742	SPRED1	sprouty-related, EVH1 domain containing 1
130589	GALM	galactose mutarotase (aldose 1-epimerase)
3418	IDH2	isocitrate dehydrogenase 2 (NADP+), mitochondrial
4744	NEFH	neurofilament, heavy polypeptide
4682	NUBP1	nucleotide binding protein 1 (MinD homolog, E. coli)
5291	PIK3CB	phosphoinositide-3-kinase, catalytic, beta polypeptide
7132	TNFRSF1A	tumor necrosis factor receptor superfamily, member 1A
5523	PPP2R3A	protein phosphatase 2, regulatory subunit B'', alpha
5326	PLAGL2	pleiomorphic adenoma gene-like 2
63910	SLC17A9	solute carrier family 17, member 9
6397	SEC14L1	SEC14-like 1 (S. cerevisiae)
51304	ZDHHC3	zinc finger, DHHC-type containing 3
29116	MYLIP	myosin regulatory light chain interacting protein
8520	HAT1	histone acetyltransferase 1
1105	CHD1	chromodomain helicase DNA binding protein 1
51312	SLC25A37	solute carrier family 25, member 37
166929	SGMS2	sphingomyelin synthase 2
171586	ABHD3	abhydrolase domain containing 3
55276	PGM2	phosphoglucomutase 2
9826	ARHGEF11	Rho guanine nucleotide exchange factor (GEF) 11
2859	GPR35	G protein-coupled receptor 35
6814	STXBP3	syntaxin binding protein 3
10866	HCP5	HLA complex P5
4354	MPP1	membrane protein, palmitoylated 1, 55kDa
5705	PSMC5	proteasome (prosome, macropain) 26S subunit, ATPase, 5
25966	C2CD2	C2 calcium-dependent domain containing 2
441212	RP9P	retinitis pigmentosa 9 pseudogene
2526	FUT4	fucosyltransferase 4 (alpha (1,3) fucosyltransferase, myeloid-specific)
6051	RNPEP	arginyl aminopeptidase (aminopeptidase B)
10062	NR1H3	nuclear receptor subfamily 1, group H, member 3
10542	HBXIP	hepatitis B virus x interacting protein
1436	CSF1R	colony stimulating factor 1 receptor
54861	SNRK	SNF related kinase
5606	MAP2K3	mitogen-activated protein kinase kinase 3
51280	GOLM1	golgi membrane protein 1
689	BTF3	basic transcription factor 3
8932	MBD2	methyl-CpG binding domain protein 2
5184	PEPD	peptidase D
637	BID	BH3 interacting domain death agonist
246330	PELI3	pellino homolog 3 (Drosophila)
59269	HIVEP3	human immunodeficiency virus type I enhancer binding protein 3
23416	KCNH3	potassium voltage-gated channel, subfamily H (eag-related), member 3
9592	IER2	immediate early response 2
387	RHOA	ras homolog gene family, member A
201627	FAM116A	family with sequence similarity 116, member A
27106	ARRDC2	arrestin domain containing 2
79801	SHCBP1	SHC SH2-domain binding protein 1
476	ATP1A1	ATPase, Na+/K+ transporting, alpha 1 polypeptide
151636	DTX3L	deltex 3-like (Drosophila)
3110	MNX1	motor neuron and pancreas homeobox 1
8869	ST3GAL5	ST3 beta-galactoside alpha-2,3-sialyltransferase 5
80867	HCG2P7	HLA complex group 2 pseudogene 7
619434	NCRNA00051	non-protein coding RNA 51
6813	STXBP2	syntaxin binding protein 2
55625	ZDHHC7	zinc finger, DHHC-type containing 7
3344	FOXN2	forkhead box N2
123	PLIN2	perilipin 2
64795	RMND5A	required for meiotic nuclear division 5 homolog A (S. cerevisiae)
8714	ABCC3	ATP-binding cassette, sub-family C (CFTR/MRP), member 3
10004	NAALADL1	N-acetylated alpha-linked acidic dipeptidase-like 1
1298	COL9A2	collagen, type IX, alpha 2
60481	ELOVL5	ELOVL family member 5, elongation of long chain fatty acids (FEN1/Elo2, SUR4/Elo3-like, yeast)
10673	TNFSF13B	tumor necrosis factor (ligand) superfamily, member 13b
126272	EID2B	EP300 interacting inhibitor of differentiation 2B
25946	ZNF385A	zinc finger protein 385A
23646	PLD3	phospholipase D family, member 3
9025	RNF8	ring finger protein 8
57826	RAP2C	RAP2C, member of RAS oncogene family
643418	LIPN	lipase, family member N
142679	DUSP19	dual specificity phosphatase 19
6556	SLC11A1	solute carrier family 11 (proton-coupled divalent metal ion transporters), member 1
55357	TBC1D2	TBC1 domain family, member 2
25977	NECAP1	NECAP endocytosis associated 1
57669	EPB41L5	erythrocyte membrane protein band 4.1 like 5
8718	TNFRSF25	tumor necrosis factor receptor superfamily, member 25
747	DAGLA	diacylglycerol lipase, alpha

79847	TMEM180	transmembrane protein 180
55619	DOCK10	dedicator of cytokinesis 10
1991	ELANE	elastase, neutrophil expressed
25849	PARM1	prostate androgen-regulated mucin-like protein 1
833	CARS	cysteinyl-tRNA synthetase
7528	YY1	YY1 transcription factor
317781	DDX51	DEAD (Asp-Glu-Ala-Asp) box polypeptide 51
29979	UBQLN1	ubiquilin 1
4117	MAK	male germ cell-associated kinase
10288	LILRB2	leukocyte immunoglobulin-like receptor, subfamily B (with TM and ITIM domains), member 2
5783	PTPN13	protein tyrosine phosphatase, non-receptor type 13 (APO-1/CD95 (Fas)-associated phosphatase)
6948	TCN2	transcobalamin II
10634	GAS2L1	growth arrest-specific 2 like 1
84255	SLC37A3	solute carrier family 37 (glycerol-3-phosphate transporter), member 3
23474	ETHE1	ethylmalonic encephalopathy 1
116372	LYPD1	LY6/PLAUR domain containing 1
91543	RSAD2	radical S-adenosyl methionine domain containing 2
5585	PKN1	protein kinase N1
911	CD1C	CD1c molecule
84067	FAM160A2	family with sequence similarity 160, member A2
54210	TREM1	triggering receptor expressed on myeloid cells 1
26030	PLEKHG3	pleckstrin homology domain containing, family G (with RhoGef domain) member 3
23623	RUSC1	RUN and SH3 domain containing 1
7106	TSPAN4	tetraspanin 4
286077	FAM83H	family with sequence similarity 83, member H
57493	HEG1	HEG homolog 1 (zebrafish)
285533	RNF175	ring finger protein 175
54458	PRR13	proline rich 13
57486	NLN	neurolysin (metallopeptidase M3 family)
113791	PIK3IP1	phosphoinositide-3-kinase interacting protein 1
157769	FAM91A1	family with sequence similarity 91, member A1
8843	GPR109B	G protein-coupled receptor 109B
6674	SPAG1	sperm associated antigen 1
79888	LPCAT1	lysophosphatidylcholine acyltransferase 1
338773	TMEM119	transmembrane protein 119
53834	FGFRL1	fibroblast growth factor receptor-like 1
4651	MYO10	myosin X
9673	SLC25A44	solute carrier family 25, member 44
224	ALDH3A2	aldehyde dehydrogenase 3 family, member A2
388692	LOC388692	hypothetical LOC388692
1066	CES1	carboxylesterase 1
55723	ASF1B	ASF1 anti-silencing function 1 homolog B (S. cerevisiae)
6583	SLC22A4	solute carrier family 22 (organic cation/ergothioneine transporter), member 4
6840	SVIL	supervillin
117155	CATSPER2	cation channel, sperm associated 2
80139	ZNF703	zinc finger protein 703
719	C3AR1	complement component 3a receptor 1
1958	EGR1	early growth response 1
8685	MARCO	macrophage receptor with collagenous structure
5621	PRNP	prion protein
55711	FAR2	fatty acyl CoA reductase 2
2857	GPR34	G protein-coupled receptor 34
8622	PDE8B	phosphodiesterase 8B
360132	FKBP9L	FK506 binding protein 9-like
4283	CXCL9	chemokine (C-X-C motif) ligand 9
256435	ST6GALNAC3	ST6 (alpha-N-acetyl-neuraminyl-2,3-beta-galactosyl-1,3)-N-acetylgalactosaminide alpha-2,6-sialyltransferase 3
10312	TCIRG1	T-cell, immune regulator 1, ATPase, H+ transporting, lysosomal V0 subunit A3
319085	ITPK1-AS1	ITPK1 antisense RNA 1 (non-protein coding)
8634	RTCD1	RNA terminal phosphate cyclase domain 1
100132417	FCGR1C	Fc fragment of IgG, high affinity Ic, receptor (CD64)
9119	KRT75	keratin 75
28959	TMEM176B	transmembrane protein 176B
199675	C19orf59	chromosome 19 open reading frame 59
80129	C6orf97	chromosome 6 open reading frame 97
147372	CCBE1	collagen and calcium binding EGF domains 1
55760	DHX32	DEAH (Asp-Glu-Ala-His) box polypeptide 32
23491	CES3	carboxylesterase 3
5222	PGA5	pepsinogen 5, group I (pepsinogen A)
54463	FAM134B	family with sequence similarity 134, member B
90362	FAM110B	family with sequence similarity 110, member B
23328	SASH1	SAM and SH3 domain containing 1
26798	SNORD51	small nucleolar RNA, C/D box 51
9397	NMT2	N-myristoyltransferase 2
3382	ICA1	islet cell autoantigen 1, 69kDa
2983	GUCY1B3	guanylate cyclase 1, soluble, beta 3
23555	TSPAN15	tetraspanin 15
60489	APOBEC3G	apolipoprotein B mRNA editing enzyme, catalytic polypeptide-like 3G
838	CASP5	caspase 5, apoptosis-related cysteine peptidase
3422	IDI1	isopentenyl-diphosphate delta isomerase 1
84953	MICALCL	MICAL C-terminal like
5140	PDE3B	phosphodiesterase 3B, cGMP-inhibited
388	RHOB	ras homolog gene family, member B
7704	ZBTB16	zinc finger and BTB domain containing 16
5333	PLCD1	phospholipase C, delta 1
84179	MFSD7	major facilitator superfamily domain containing 7
817	CAMK2D	calcium/calmodulin-dependent protein kinase II delta
26031	OSBPL3	oxysterol binding protein-like 3
84446	BRSK1	BR serine/threonine kinase 1
147699	PPM1N	protein phosphatase, Mg2+/Mn2+ dependent, 1N (putative)
55181	C17orf71	chromosome 17 open reading frame 71
657	BMPR1A	bone morphogenetic protein receptor, type IA
57579	FAM135A	family with sequence similarity 135, member A
55897	MESP1	mesoderm posterior 1 homolog (mouse)
89958	C9orf140	chromosome 9 open reading frame 140
58526	MID1IP1	MID1 interacting protein 1 (gastrulation specific G12 homolog (zebrafish))

441251	SPDYE7P	speedy homolog E7 (Xenopus laevis), pseudogene
23639	LRRC6	leucine rich repeat containing 6
3426	CFI	complement factor I
23046	KIF21B	kinesin family member 21B
165530	CLEC4F	C-type lectin domain family 4, member F
6389	SDHA	succinate dehydrogenase complex, subunit A, flavoprotein (Fp)
9821	RB1CC1	RB1-inducible coiled-coil 1
83787	ARMC10	armadillo repeat containing 10
10461	MERTK	c-mer proto-oncogene tyrosine kinase
399761	BMS1P5	BMS1 pseudogene 5
910	CD1B	CD1b molecule
10544	PROCR	protein C receptor, endothelial
53826	FXYD6	FXYD domain containing ion transport regulator 6
57591	MKL1	megakaryoblastic leukemia (translocation) 1
4810	NHS	Nance-Horan syndrome (congenital cataracts and dental anomalies)
286333	NCRNA00256A	non-protein coding RNA 256A
348110	C15orf38	chromosome 15 open reading frame 38
2001	ELF5	E74-like factor 5 (ets domain transcription factor)
10435	CDC42EP2	CDC42 effector protein (Rho GTPase binding) 2
712	C1QA	complement component 1, q subcomponent, A chain
5239	PGM5	phosphoglucomutase 5
717	C2	complement component 2
25903	OLFML2B	olfactomedin-like 2B
2213	FCGR2B	Fc fragment of IgG, low affinity IIb, receptor (CD32)
79762	C1orf115	chromosome 1 open reading frame 115
196996	GRAMD2	GRAM domain containing 2
65124	ANKRD57	ankyrin repeat domain 57
913	CD1E	CD1e molecule
10855	HPSE	heparanase
1959	EGR2	early growth response 2
113277	TMEM106A	transmembrane protein 106A
57121	LPAR5	lysophosphatidic acid receptor 5
10863	ADAM28	ADAM metallopeptidase domain 28
9249	DHRS3	dehydrogenase/reductase (SDR family) member 3
118932	ANKRD22	ankyrin repeat domain 22
339390	CLEC4G	C-type lectin domain family 4, member G
222962	SLC29A4	solute carrier family 29 (nucleoside transporters), member 4
11279	KLF8	Kruppel-like factor 8
81552	VOPP1	vesicular, overexpressed in cancer, prosurvival protein 1
151011	SEPT10	septin 10
57010	CABP4	calcium binding protein 4
3386	ICAM4	intercellular adhesion molecule 4 (Landsteiner-Wiener blood group)
9332	CD163	CD163 molecule
9454	HOMER3	homer homolog 3 (Drosophila)
2350	FOLR2	folate receptor 2 (fetal)
29956	LASS2	LAG1 homolog, ceramide synthase 2
389634	LOC389634	hypothetical LOC389634
11199	ANXA10	annexin A10
5738	PTGFRN	prostaglandin F2 receptor negative regulator
6934	TCF7L2	transcription factor 7-like 2 (T-cell specific, HMG-box)
3815	KIT	v-kit Hardy-Zuckerman 4 feline sarcoma viral oncogene homolog
83716	CRISPLD2	cysteine-rich secretory protein LCCL domain containing 2
84668	FAM126A	family with sequence similarity 126, member A
9034	CCRL2	chemokine (C-C motif) receptor-like 2
23166	STAB1	stabilin 1
6355	CCL8	chemokine (C-C motif) ligand 8
4145	MATK	megakaryocyte-associated tyrosine kinase
150365	MEI1	meiosis inhibitor 1
11326	VSIG4	V-set and immunoglobulin domain containing 4
3581	IL9R	interleukin 9 receptor
115290	FBXO17	F-box protein 17
4640	MYO1A	myosin IA
117144	CATSPER1	cation channel, sperm associated 1
5159	PDGFRB	platelet-derived growth factor receptor, beta polypeptide
344148	NCKAP5	NCK-associated protein 5
338440	ANO9	anoctamin 9
10666	CD226	CD226 molecule
51474	LIMA1	LIM domain and actin binding 1
4023	LPL	lipoprotein lipase
4923	NTSR1	neurotensin receptor 1 (high affinity)
5337	PLD1	phospholipase D1, phosphatidylcholine-specific
84057	MND1	meiotic nuclear divisions 1 homolog (S. cerevisiae)
79630	C1orf54	chromosome 1 open reading frame 54
79412	KREMEN2	kringle containing transmembrane protein 2
79971	WLS	wntless homolog (Drosophila)
115123	03-Mar	membrane-associated ring finger (C3HC4) 3
83988	NCALD	neurocalcin delta
153769	SH3RF2	SH3 domain containing ring finger 2
3753	KCNE1	potassium voltage-gated channel, Isk-related family, member 1
4897	NRCAM	neuronal cell adhesion molecule
326342	EMR4P	egf-like module containing, mucin-like, hormone receptor-like 4 pseudogene
55615	PRR5	proline rich 5 (renal)
2048	EPHB2	EPH receptor B2
100505501	LOC100505501	hypothetical LOC100505501
4856	NOV	nephroblastoma overexpressed gene
1593	CYP27A1	cytochrome P450, family 27, subfamily A, polypeptide 1
126669	SHE	Src homology 2 domain containing E
57447	NDRG2	NDRG family member 2
1903	S1PR3	sphingosine-1-phosphate receptor 3
7450	VWF	von Willebrand factor
51760	SYT17	synaptotagmin XVII
5539	PPY	pancreatic polypeptide
30835	CD209	CD209 molecule
100288092	LOC100288092	hypothetical LOC100288092
55384	MEG3	maternally expressed 3 (non-protein coding)

2199	FBLN2	fibulin 2
